# Genetics of a diverse soft winter wheat population for pre-harvest sprouting, agronomic, and flour quality traits

**DOI:** 10.3389/fpls.2023.1137808

**Published:** 2023-06-06

**Authors:** Nisha Patwa, Bryan W. Penning

**Affiliations:** United States Department of Agriculture – Agricultural Research Service (USDA-ARS) Corn, Soybean and Wheat Quality Research Unit, Wooster, OH, United States

**Keywords:** wheat, pre-harvest spouting, flour quality, grain quality, genetics, genome-wide association

## Abstract

Soft winter wheat has been adapted to the north-central, north-western, and south-central United States over hundreds of years for optimal yield, height, heading date, and pathogen and pest resistance. Environmental factors like weather affect abiotic traits such as pre-harvest sprouting resistance. However, pre-harvest sprouting has rarely been a target for breeding. Owing to changing weather patterns from climate change, pre-harvest sprouting resistance is needed to prevent significant crop losses not only in the United States, but worldwide. Twenty-two traits including age of breeding line as well as agronomic, flour quality, and pre-harvest sprouting traits were studied in a population of 188 lines representing genetic diversity over 200 years of soft winter wheat breeding. Some traits were correlated with one another by principal components analysis and Pearson’s correlations. A genome-wide association study using 1,978 markers uncovered a total of 102 regions encompassing 226 quantitative trait nucleotides. Twenty-six regions overlapped multiple traits with common significant markers. Many of these traits were also found to be correlated by Pearson’s correlation and principal components analyses. Most pre-harvest sprouting regions were not co-located with agronomic traits and thus useful for crop improvement against climate change without affecting crop performance. Six different genome-wide association statistical models (GLM, MLM, MLMM, FarmCPU, BLINK, and SUPER) were utilized to search for reasonable models to analyze soft winter wheat populations with increased markers and/or breeding lines going forward. Some flour quality and agronomic traits seem to have been selected over time, but not pre-harvest sprouting. It appears possible to select for pre-harvest sprouting resistance without impacting flour quality or the agronomic value of soft winter wheat.

## 1 Introduction

Soft winter wheat has been improved primarily for agronomic traits such as yield, heading date (HD), plant height (Hght), and resistance to pathogens and pests for hundreds of years (Hedden, 2003; Cavanagh et al., 2013; Ward et al., 2019). However, other trait improvements could increase long-term product success, such as improved grain or flour quality traits and resistance to pre-harvest sprouting (PHS). This is a weather-dependent abiotic stress affecting quality and marketability of grains around the world (Patwa and Penning, 2020). Alleles to improve traits not selected in modern varieties may exist in older breeding lines and await discovery. Marker-assisted selection could be used to improve wheat varieties. However, with increases in population and food demand requiring doubling of production, quality improvement cannot come at the expense of agronomic traits (Ray et al., 2013).

PHS, measured as grain soundness, occurs throughout the world and can damage wheat, rice, barley, maize, sorghum, and rye leading to yield losses of 10%–50% and up to 30% reduction in sale value (Patwa and Penning, 2020). It has become more prevalent in Africa, Australia, Canada, China, Europe, India, Japan, and the United States from climate change (Singh et al., 2021). PHS is caused by complex physiological and biochemical mechanisms including seed dormancy, abscisic acid concentration and sensitivity, alpha amylase activity (AA), differential water imbibition due to seed coat and spike morphology, humidity, and temperature (King and Richards, 1984; DePauw and McCaig, 1991; Yanagisawa et al., 2005; Chono et al., 2006; Patwa and Penning, 2020). However, less is known about the genetics of PHS resistance needed to produce markers meaning that marker development is difficult. Current knowledge of the genetic factors affecting PHS is limited and includes signaling genes such as *TaPHS1/MOTHER OF FLOWERING TIME (MFT), MAP KINASE KINASE 3 (MKK3)*, and *VIVIPAROUS 1 (VP1)*; genes involved in abscisic acid synthesis and degradation like *9-cis-epoxycarotenoid dioxygenase (HvNCED1)* and *ABA 8’-hydroxylase (HvCYP707A1)*; and the mutant *ENHANCED RESPONSE TO ABA8 (ERA8)* in white spring wheat (Nakamura and Toyama, 2001; Chono et al., 2006; Nakamura et al., 2011; Lin et al., 2016; Martinez et al., 2016; Torada et al., 2016; Vetch et al., 2019).

Flour quality traits are used to describe the complex physical changes that occur during baking. The impact of these traits can differ depending on the baked product. Water absorption from damaged starch, measured by sodium carbonate solvent retention capacity (NaSRC), and arabinoxylans, measured by sucrose solvent retention capacity (SucSRC), can lead to brittle products that take longer to bake (Souza et al., 2012). Higher gluten strength as measured by lactic acid solvent retention capacity (LASRC) or adjusted lactic acid solvent retention capacity (LAAdSRC) binds water during leavening to better hold dough together (dough strength) and is useful for rising pound cake or biscuits (Wilderjans et al., 2008; Ma and Baik, 2018). Increased flour protein content (FlProt) also increases dough strength although lower protein is desired by the baking industry for most soft winter wheat baked products besides crackers (Deng et al., 2021). All above-mentioned solvent retention capacity (SRC) measurements interact to affect water absorption in flour measured by water absorption solvent retention capacity (WatSRC) (Souza et al., 2012). Water absorption along with flour yield (FlYld) and softness equivalence (SftEqv) can impact milling and baking performance as well. SftEqv, SucSRC, and WatSRC have been previously correlated with whole wheat cookie diameter (CkDia). Measurement of CkDia after baking of cookie dough made from flour of different wheat varieties but of the same weight and shape estimates cookie spread during baking. Texture measured by cookie top grade (CkTpGr) and consistent spread are important to the baking industry for uniform products (Souza et al., 2011).

Several previous studies have defined PHS resistance or flour quality traits on nearly every chromosome, but most are of small effect and used modern breeding lines (Gao et al., 2013; Cabral et al., 2014; Lin et al., 2016). PHS and flour quality traits have infrequently been bred into soft winter wheat due to labor, cost, and the need for time-consuming analyses. One study not only improved PHS resistance by breeding a variety related to Tom Thumb into a more modern variety but also affected height (Bhatt et al., 1977). With the advent of marker-assisted selection in plant breeding, providing reliable trait-improving markers could allow flour quality and PHS resistance improvement while maintaining agronomically important qualities.

To study the effects of many traits, a population encompassing diversity of breeding lines from the early 1800s to the early 2000s was developed based on previous milling and baking performance using a long-flow Allis-Chalmers milling system by the USDA-Agricultural Research Service Soft Wheat Quality Lab. Included varieties represent variation in important crop traits when grown in eastern North America and were available for study (Souza et al., 2012). The population displayed similar structure and more variation than a much larger modern elite set of breeding lines (Cabrera et al., 2014). One factor apparent in the population structure of the diversity breeding lines was age of the release (Cabrera et al., 2014). Few studies in soft winter wheat have investigated the impact of selective breeding to genetic changes in such a diverse set of traits available in this population. The selection of yield in elite breeding lines may have inadvertently reduced overall diversity of important but untested quality traits (Cabrera et al., 2014). Wide values for many diverse traits in this population allowed discovery of chromosome regions for improvement and overlap with other traits. A completed sequence and sets of markers in wheat allowed for the performance of a genome-wide association study (GWAS) for trait mapping (IWGSC, 2014). The GAPIT3 platform contains several GWAS models with slightly varied assumptions and regression models that could impact the association of significant markers with a phenotype (Wang and Zhang, 2021). The population size of 188 with 1,978 markers provided an opportunity to test how different GWAS models would perform in a real population of soft winter wheat rather than a simulated population without a burdensome amount of computation.

The goal of this research was to find regions of the genome that could provide resistance to PHS, an important abiotic threat to wheat crops. Also, this study sought to determine if resistance to PHS may overlap with other important flour quality or agronomic traits. This would indicate if breeding efforts over the past ~200 years impacted previously unmeasured PHS and flour quality traits in pursuit of other traits such as yield, pests, and disease resistance. With the pressures of an increasing population requiring more food of higher quality at a time when climate change can cause disruptions in the food supply, it has become more important to protect against abiotic stresses such as PHS without impacting agronomic traits or flour quality in wheat.

## 2 Materials and methods

### 2.1 Plant material

Sixty grams of seed per plot for each of the 188 members of the historic diversity population described in Souza et al. (2012) and available individually (https://www.ars-grin.gov or http://wheatpedigree.net) as listed in **Supplemental File S1** were planted in 3 m × 3 m plots of six rows across a plot to achieve a full block of plants with 0.6-m alleys between each 3 m × 3 m plot at Schaffter, Snyder, and/or King farms at Ohio State University. Two sets of plots were grown each year, one for artificial irrigation of the field and one left in the field and harvested after a natural rain event. Fields were grown in successive years until at least 3 years of data could be obtained as not every year featured a natural rain event within the harvest window. Fields were tilled and pre-dressed with 28 kg/hectare of nitrogen prior to planting in October of each year. Nitrogen was reapplied at 101 kg/hectare in April of each year followed by 1 L/hectare of Huskie broadleaf herbicide. A border of >6 m of wheat or rye was used as a buffer.

For irrigated PHS tests, plots were treated in the field with overhead sprinklers simulating 2.5–5.0 cm of rain for 2 days (Irr). A second set of plots was left in the field until a natural rain event (Nat) occurred (Sneller et al., 2010). Each whole plot was harvested by a Wintersteiger Classic combine (www.wintersteiger.com). For the artificial spike wetting test (Art), ~30 spikes were hand harvested from the non-irrigated plot at maturity (no green on the spikes) in brown paper bags. Spikes were dried in the bag at ~29˚C for 5 days, and then placed upright ~2.5 cm apart in Styrofoam blocks by their stems and subjected to 95% humidity in a growth chamber with half sodium/half metal halide lights at 200–300 μmol m^−2^ s^−1^ and 20˚C for 16 h and no lights and 15˚C for eight h. Spikes were soaked with water from a spray bottle every 12 h. After 3 days, spikes were dried in the greenhouse for 1–2 days, hand threshed, placed in heavy manila envelopes, and stored at −20˚C. All collected seeds were cleaned of debris by blowing air to remove the lighter chaff. All PHS treatments were performed for 3–4 years (harvested 2018–2021 for Art, 2019–2021 for Irr, and 2018, 2019, and 2022 for Nat).

### 2.2 Chemical and physical tests

Grain and flour quality tests for FlProt, test weight (TstWt), WatSRC, NaSRC, SucSRC, LASRC, LAAdSRC, SftEqv, FlYld, CkDia, and CkTpGr were performed on sound grain (not exposed to PHS) and calculated as described previously (Souza et al., 2012; Cabrera et al., 2015). Briefly, FlProt was determined by near-infrared reflectance on a Unity Spectra-Star 2200 (Columbia, MD), TstWt was determined by weight of 1,000 grains, all five SRCs were determined by American Association of Cereal Chemists (AACC) method 56-11.02 (AACC, 2010a), CkDia and CkTpGr were determined by AACC method 10-52.02 (AACC, 2010b), and FlYld and SftEqv were determined based on milling grain tempered to 15% moisture through a modified Brabender Quadramat Junior flour mill (Cabrera et al., 2015).

HD was recorded visually walking the field daily and recording the first day 50% of wheat spikes had fully emerged from the boot jack. Values were calculated by assigning the first date a variety emerged as day 1 and adding the appropriate number of days for each variety that emerged afterward. Hght was recorded in centimeters at maturity, except the first year collected in inches and converted to centimeters.

Soft winter wheat has been primarily bred for improvement of traits such as yield, height, heading days, or pathogen and pest resistance over ~200 years. Other traits may have been altered because they were located near genes for selected traits. To observe if PHS or flour quality traits may have been impacted, a comparative measure for how long ago the variety was released by breeders was developed. If this measure co-located with PHS or flour quality traits, it would indicate their inadvertent selection, especially if they also co-locate with agronomic traits. Age of breeding line (Age) was calculated by subtracting the release year of the line from 2020. Awnless or bearded (Awns) and red or white seed color (SdClr) was determined by visual observation and accession reports.

PHS treatments were tested for grain soundness by Falling Number (FN) and AA tests. Threshed and cleaned seeds were ground in a UDY Mill model 3010-014 with a 1-mm screen (www.udyone.com). FN was measured using a Perten Falling Number machine 100 or 1000 (www.perkinelmer.com) in 20 ml of water with grams of ground seeds based on moisture content per manufacturer’s manual Table III (Perten Instruments AB, 2016). Moisture content was measured using AACC method 44-16.01 (1999). The paste was vortexed in a Perten Shakematic (www.perkinelmer.com) for 15 s before placing in the Falling Number machine following AACC method 56-81.03 (1992). Alpha amylase activity was calculated following AACC method 22-02.01 (2002) with an alpha amylase assay kit (Ceralpha Method) by Megazyme (www.megazyme.com) with the following modification. Instead of 7 g of ground seeds in 40 ml of extraction solution in 50-ml tubes, 0.3 g of ground seeds was extracted in 2 ml of extraction solution in a 48-well format (Corning #P-5ML-48-C, www.corning.com). Absorbance was determined colorimetrically in a 96-well plate format (Fisherbrand #21377203, www.fishersci.com) using a BioTek plate reader (www.biotek.com) at 405 nm. Activity was calculated using the manufacturer’s standard method for wheat (www.megazyme.com).

### 2.3 Genetics and statistical analysis

Multiple years or locations of trait data, three for irrigated alpha amylase activity (IrrAA), irrigated Falling Number (IrrFN), natural alpha amylase activity (NatAA), natural Falling Number (NatFN), TstWt, CkDia, and CkTpGr, and four for artificial alpha amylase activity (ArtAA), artificial Falling Number (ArtFN), FlProt, FlYld, HD, Hght, LAAdSRC, LASRC, NaSRC, SftEqv, SucSRC, and WatSRC were combined into Best Linear Unbiased Predictors (BLUP) using R version 4.0.4 and metafor package version 2.4-0 for samples using means and standard deviations calculated in Microsoft Excel (Viechtbauer, 2010; R Core Team, 2022). Histograms for the BLUP of each trait were plotted using the Histogram data analysis tool in Microsoft Excel and a Shapiro–Wilk normality test was performed using the R command shapiro.test(*trait*) (R Core Team, 2022).

Principal components analysis (PCA) was performed using pcaMethods version 1.78.0 from Bioconducter version 3.10.1 and factoextra version 1.0.7 in R version 3.6.3 necessary to run the combination of tools (Stacklies et al., 2007; Huber et al., 2015; Kassambara and Mundt, 2020; R Core Team, 2022). Microsoft Excel was used to graph combinations of principal components (PCs) 1, 2, and 3. Pearson’s correlation coefficients and their associated *p*-values were calculated using the rcorr command from the Hmisc library in R version 3.63, copied into comma delimited files using the write.csv command and merged into one table in Microsoft Excel.

A GWAS was performed with GAPIT3 (GitHub version on 10/14/22 from www.zzlab.net) under R version 4.0.4 running in RStudio version 4.2.1 using six models: general linear model (GLM), mixed linear model (MLM), multiple loci mixed model (MLMM), fixed and random model circulating probability unification (FarmCPU), Bayesian-information and linkage-disequilibrium iteratively nested keyway (BLINK), and settlement of MLM under progressively exclusive relationship (SUPER) to discover any differences model type might play in locating significant markers for different phenotypes in a soft winter wheat population (Wang et al., 2014a; Tang et al., 2016; Wang and Zhang, 2021; R Core Team, 2022; RStudio Team, 2022). The markers were derived from the 9K iSelect single-nucleotide polymorphism (SNP) array by removing markers that could not be uniquely mapped to a single chromosome using five recombinant inbred line populations with parents in the GWAS population (Cavanagh et al., 2013; Cabrera et al., 2015). In addition to the stacked Manhattan plots and quartile–quartile (QQ) plots generated by GAPIT3, significant results from GAPIT3 for each trait region were reported using Bonferroni significance cutoffs calculated using an alpha of 0.05 divided by the number of markers per chromosome (Wang and Zhang, 2021; Hill et al., 2022). Locations of significant quantitative trait nucleotides (QTNs) were visualized using chromPlot in R with R script in **File S2** (Orostica and Verdugo, 2016; R Core Team, 2022).

## 3 Results

### 3.1 Statistical analysis

Alpha amylase activity calculated in three separate years for a subset of 20 samples had a linear correlation of 0.98, 0.95, and 0.93 between the standard and modified protocol (data not shown). Means, standard deviations, and number of samples for each trait are in **File S1** and histograms of each trait by year and BLUP are in **File S5**. Shapiro–Wilk test statistics and *p*-values for normality of all traits are listed in **Supplemental Table S1**. Relatedness of 22 agronomic, flour quality (on sound grain), and PHS traits was measured by Pearson’s correlation and PCA combining years or locations using BLUPs. R scripts are in **File S2**. Pearson’s correlations with a *p*-value < 0.001 were considered significant. FN traits negatively correlated with AA traits. Seed color and all PHS-related tests were significantly correlated with each other. All but IrrAA were correlated with TstWt. Some PHS measures such as ArtFN appeared slightly correlated with flour quality traits including WatSRC, LASRC, LAAdSRC, and SucSRC or CkTpGr. Only ArtFN appeared correlated with HD or Hght. Age (years since release) was positively correlated with Hght, FlProt, and HD, and negatively correlated with CkDia, CkTpGr, and SftEqv. SdClr was positively correlated with TstWt and most SRC and negatively correlated with HD, Hght, CkTpGr, and CkDia. Awns were uncorrelated with other traits. Flour protein was positively correlated with Hght and most SRCs and negatively correlated with other flour quality traits while most SRCs were positively correlated with each other and negatively correlated with other flour quality traits with significant correlations (*p*-value <0.001) of −0.24 to 0.94 (**Table 1**).

**Table 1:** Pearson’s correlations of 22 soft winter wheat traits with significance across top and right correlations with *p*-values <0.001 in bold.

	Age	SdClr	Awns	ArtAA	IrrAA	NatAA	ArtFN	IrrFN	NatFN	TstWt	FlProt	WatSRC	NaSRC	SucSRC	LASRC	LAAdSRC	SftEqv	FlYld	CkDia	CkTpGr	HD	Hght
**Age**		7.E−02	1.E−01	2.E−01	5.E−01	7.E−01	3.E−01	7.E−01	1.E+00	4.E−01	**7.E−15**	3.E−02	3.E−01	4.E−02	1.E−01	2.E−01	**3.E−05**	3.E−02	**2.E−07**	**2.E−05**	**3.E−10**	**0**
**SdClr**	−0.13		5.E−01	**4.E−07**	**9.E−11**	**2.E−11**	**3.E−15**	**3.E−06**	**3.E−11**	**2.E−11**	9.E−02	**9.E−05**	8.E−03	**1.E−08**	**2.E−09**	**1.E−09**	7.E−01	3.E−01	**4.E−04**	**9.E−05**	**3.E−10**	**6.E−08**
**Awns**	0.11	−0.05		2.E−01	5.E−02	3.E−02	2.E−02	3.E−02	4.E−02	2.E−02	6.E−01	6.E−01	3.E−02	6.E−01	6.E−01	5.E−01	3.E−02	6.E−01	2.E−01	3.E−01	1.E−01	8.E−01
**ArtAA**	0.10	**−0.36**	0.10		**6.E−12**	**1.E−08**	**0**	**4.E−11**	**4.E−10**	**4.E−07**	9.E−01	2.E−03	1.E−03	**3.E−04**	1.E−02	6.E−03	8.E−01	3.E−01	9.E−03	3.E−03	3.E−02	6.E−03
**IrrAA**	0.05	**−0.45**	0.15	**0.47**		**0**	**1.E−12**	**0**	**0**	**3.E−05**	6.E−01	5.E−02	1.E−01	3.E−02	**6.E−04**	**2.E−04**	6.E−01	6.E−01	2.E−02	5.E−02	4.E−02	3.E−02
**NatAA**	0.03	**−0.46**	0.16	**0.40**	**0.67**		**3.E−12**	**0**	**0**	**2.E−08**	2.E−01	1.E−01	8.E−01	1.E−02	**8.E−04**	1.E−03	8.E−01	6.E−01	1.E−02	6.E−02	1.E−01	2.E−01
**ArtFN**	−0.08	**0.53**	−0.17	**−0.69**	**−0.49**	**−0.48**		**7.E−13**	**7.E−13**	**1.E−08**	6.E−01	**8.E−05**	4.E−03	**6.E−06**	**2.E−04**	**9.E−05**	1.E+00	8.E−02	3.E−03	**8.E−04**	**3.E−04**	**9.E−04**
**IrrFN**	0.03	**0.33**	−0.15	**−0.46**	**−0.81**	**−0.57**	**0.49**		**0**	1.E−03	3.E−01	8.E−02	4.E−01	6.E−02	5.E−03	6.E−03	9.E−01	6.E−01	2.E−02	7.E−02	1.E+00	7.E−01
**NatFN**	0.00	**0.46**	−0.15	**−0.44**	**−0.76**	**−0.80**	**0.49**	**0.74**		**6.E−06**	2.E−01	9.E−02	5.E−01	1.E−02	1.E−03	1.E−03	1.E+00	5.E−01	7.E−03	5.E−02	3.E−01	8.E−02
**TstWt**	−0.07	**0.47**	−0.17	**−0.36**	**−0.30**	**−0.39**	**0.40**	0.24	**0.32**		**1.E−05**	**1.E−05**	7.E−02	**4.E−06**	**2.E−09**	**9.E−07**	**8.E−04**	9.E−01	**4.E−06**	**9.E−05**	**8.E−07**	2.E−02
**FlProt**	**0.53**	0.12	−0.04	−0.01	−0.03	−0.09	0.04	0.07	0.09	**0.31**		**8.E−05**	3.E−01	**4.E−07**	**1.E−10**	1.E−01	**0**	**1.E−05**	**0**	**0**	1.E−01	**5.E−09**
**WatSRC**	0.16	**0.28**	0.04	−0.23	−0.14	−0.11	**0.28**	0.13	0.13	**0.31**	**0.28**		**0**	**0**	**1.E−10**	**4.E−08**	**5.E−08**	**9.E−14**	**0**	**3.E−12**	4.E−03	5.E−02
**NaSRC**	−0.08	0.19	0.16	−0.23	−0.12	−0.02	0.21	0.06	0.05	0.13	−0.07	**0.68**		**0**	**5.E−07**	**1.E−09**	**4.E−04**	**3.E−13**	**1.E−09**	**3.E−06**	2.E−03	**7.E−04**
**SucSRC**	0.15	**0.40**	0.04	**−0.26**	−0.16	−0.18	**0.32**	0.14	0.19	**0.33**	**0.36**	**0.76**	**0.74**		**0**	**0**	4.E−01	**0**	**0**	**0**	2.E−03	1.E−01
**LASRC**	0.11	**0.42**	0.03	−0.18	**−0.25**	**−0.24**	**0.27**	0.20	0.24	**0.42**	**0.45**	**0.45**	**0.36**	**0.70**		**0**	2.E−01	**6.E−07**	**0**	**4.E−16**	**1.E−05**	3.E−01
**LAAdSRC**	−0.10	**0.42**	0.05	−0.20	**−0.27**	−0.23	**0.28**	0.20	0.23	**0.35**	0.12	**0.39**	**0.43**	**0.64**	**0.94**		1.E−01	**1.E−04**	**2.E−08**	**6.E−08**	**3.E−08**	**9.E−04**
**SftEqv**	**−0.30**	−0.03	0.16	−0.02	−0.04	0.02	0.00	−0.01	0.00	**−0.24**	**−0.59**	**−0.39**	**0.26**	−0.07	−0.09	0.12		1.E−01	**1.E−09**	**1.E−04**	7.E−01	1.E−03
**FlYld**	−0.16	−0.08	−0.04	0.08	0.03	−0.03	−0.13	−0.04	−0.05	0.01	**−0.31**	**−0.51**	**−0.50**	**−0.59**	**−0.35**	**−0.28**	0.12		**6.E−15**	**4.E−13**	7.E−01	5.E−01
**CkDia**	**−0.37**	**−0.26**	0.09	0.19	0.17	0.18	−0.22	−0.17	−0.20	**−0.33**	**−0.64**	**−0.68**	**−0.43**	**−0.69**	**−0.57**	**−0.40**	**0.42**	**0.53**		**0**	5.E−01	9.E−03
**CkTpGr**	**−0.31**	**−0.28**	0.08	0.21	0.14	0.14	**−0.24**	−0.13	−0.15	**−0.28**	**−0.59**	**−0.48**	**−0.33**	**−0.64**	**−0.55**	**−0.38**	**0.28**	**0.50**	**0.83**		3.E−01	4.E−02
**HD**	**0.44**	**−0.44**	0.11	0.16	0.15	0.12	**−0.26**	0.00	−0.07	**−0.35**	0.12	−0.21	−0.22	−0.22	**−0.31**	**−0.39**	−0.03	−0.03	0.05	0.08		**0**
**Hght**	**0.62**	**−0.38**	0.02	0.20	0.16	0.10	**−0.24**	−0.03	−0.13	−0.17	**0.41**	−0.14	**−0.24**	−0.11	−0.08	**−0.24**	−0.24	−0.05	−0.19	−0.15	**0.68**	

Age, number of years since line released; SdClr, seed color; Awns, presence of awns, ArtAA, wetted spikes alpha amylase test, IrrAA, field irrigated alpha amylase test, NatAA, naturally occurring alpha amylase test, ArtFN, wetted spikes falling number test, IrrFN, field irrigated falling number test, NatFN, naturally occurring falling number test, TstWt, test weight, FlProt, flour protein, WatSRC, water solvent retention capacity, NaSRC, sodium carbonate solvent retention capacity, SucSRC, sucrose solvent retention capacity, LASRC, lactic acid solvent retention capacity, LAAdSRC, adjusted lactic acid solvent retention capacity, SftEqv, softness equivalence, FlYld, flour yield, CkDia, cookie diameter, CkTpGr, cookie top grade, HD, heading days after first day, Hght, plant height.

The first three PCs of all traits represented 31%, 18%, and 12% of variation, respectively, and were considered significant based on a plot of Eigenvalues and cumulative variance (**Supplemental Figure S1**).

**Figure 1:**
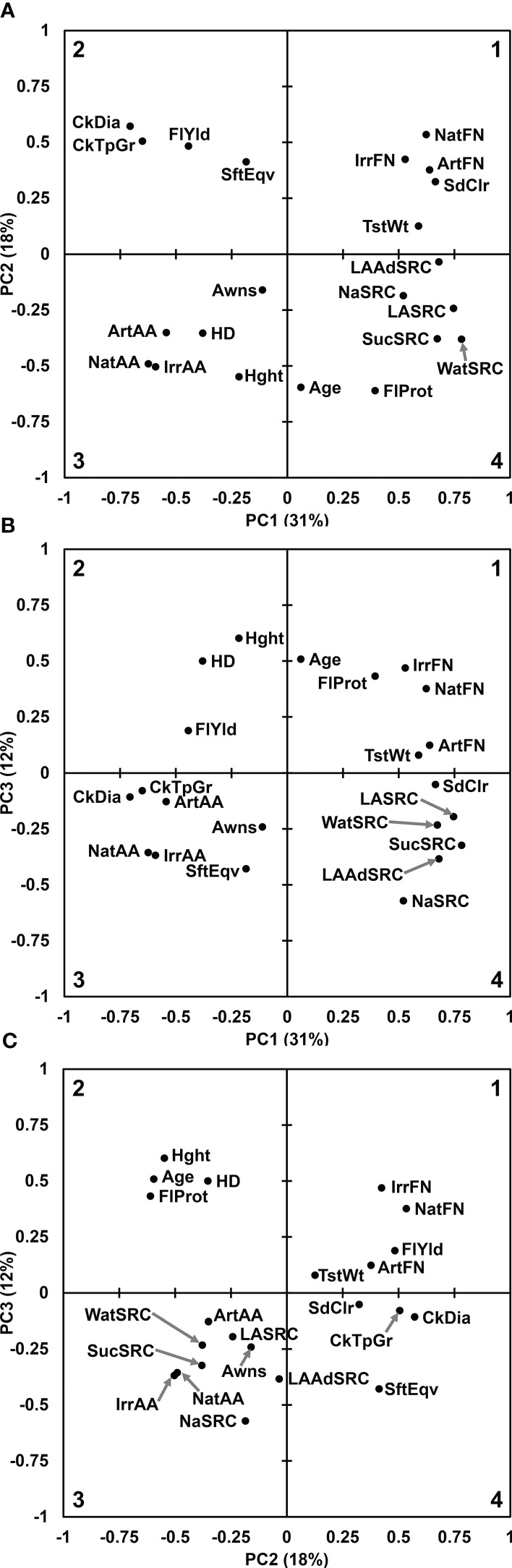
Principal components analysis for all 22 trait contributions. **(A)** Principal Component 1 *vs*. Principal Component 2. **(B)** Principal Component 1 *vs*. Principal Component 3. **(C)** Principal Component 2 *vs*. Principal Component 3. Quadrant numbers shown in corners. Gray arrows point to circles where labels would be too close to read. Age of breeding line (Age), Artificial alpha amylase activity (ArtAA), Artificial falling number (ArtFN), Awnless or bearded (Awns), Cookie Diameter (CkDia), Cookie top grade (CkTpGrd), Flour Protein (FlProt), Flour Yield (FlYld), Heading date (HD), Plant height (Hght), Irrigated alpha amylase activity (IrrAA), Irrigated falling number (IrrFN), Adjusted Lactic Acid SRC (LAAdSRC), Lactic Acid SRC (LASRC), Sodium Carbonate SRC (NaSRC), Natural alpha amylase activity (NatAA), Natural falling number (NatFN), red or white seed color (SdClr), Softness Equivalence (SftEqv), Sucrose SRC (SucSRC), test weight (TstWt), and Water SRC (WatSRC).

The trait contribution biplot of PC1 *vs*. PC2 mirrored many of the Pearson’s correlation comparisons, accounting for the positive or negative correlation as grouped in quadrant or in opposite quadrant by PC1, PC2, or both axes. FN and SdClr were grouped together tightly and loosely with TstWt in quadrant 1. Alpha amylase activity measures were tightly grouped in quadrant 3 with HD loosely associated. Non-SRC flour quality traits except FlProt grouped loosely in quadrant 2. The SRCs grouped tightly in quadrant 4. Awns, FlProt, Hght, and Age were not well captured by the first two principal components and appeared separated from the main groups (**Figure 1A**). The trait contribution biplot of PC1 *vs*. PC3 showed similar groupings to PC1 *vs*. PC2 with the exception of CkDia and CkTpGr quality traits being more closely grouped with AA measures and many agronomic factors flipping to the positive PC3 axis (**Figures 1A, B**). The PC2 *vs*. PC3 trait contribution biplot revealed a strong grouping of Age, Hght, HD, and FlProt in quadrant 2, agreeing with Pearson’s correlations and the SRC grouping in quadrant 3 between AA measures (**Figure 1C**, **Table 1**).

### 3.2 Genome-wide association study

**Figure 2:**
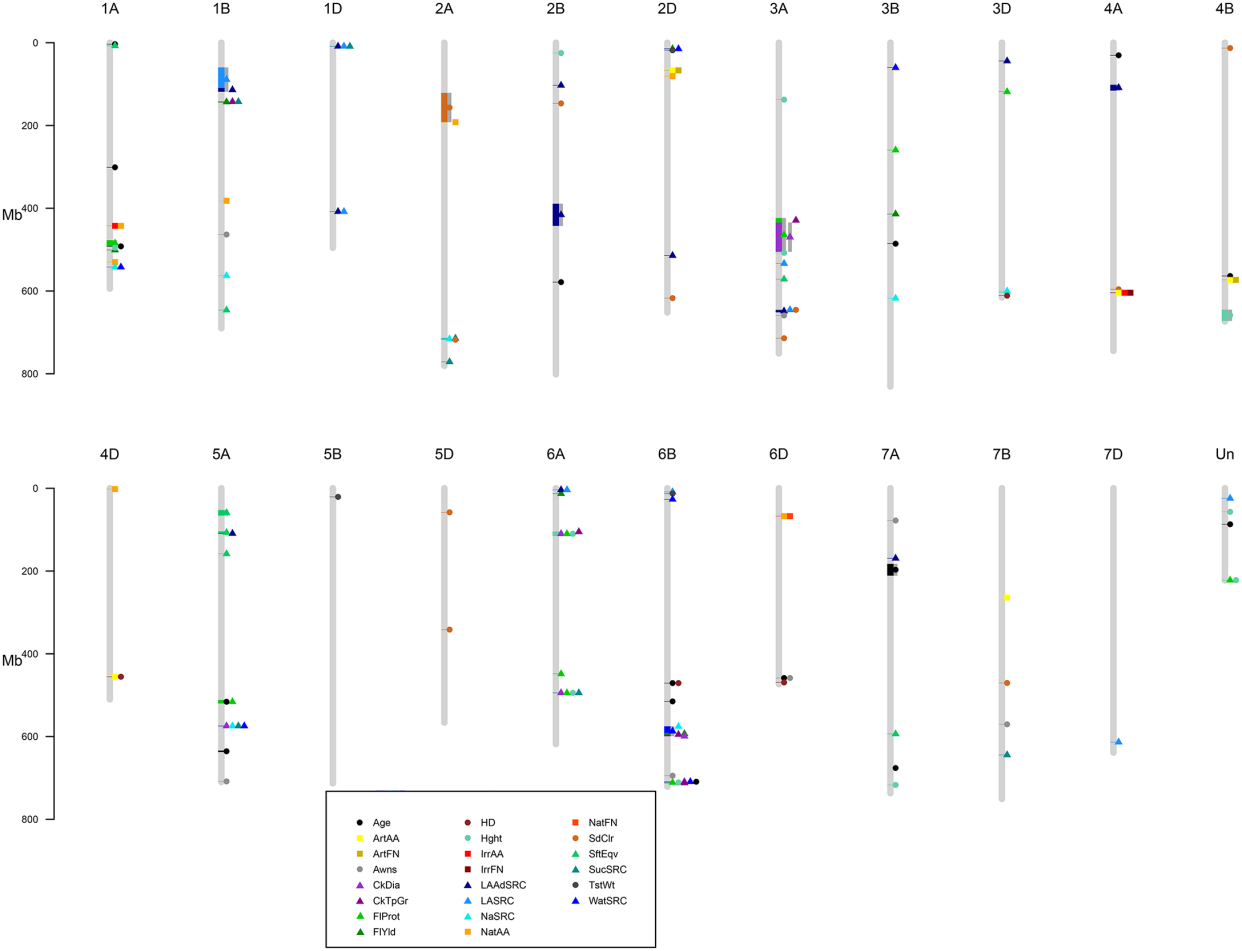
Regions of significant QTN for 22 traits. Circles are agronomic and physical traits, squares are pre-harvest sprouting, and triangles are flour quality. Gray bars surrounding symbols indicate a range of significant markers. Legend describes specific traits. Age of breeding line (Age), Artificial alpha amylase activity (ArtAA), Artificial falling number (ArtFN), Awnless or bearded (Awns), Cookie Diameter (CkDia), Cookie top grade (CkTpGrd), Flour Protein (FlProt), Flour Yield (FlYld), Heading date (HD), Plant height (Hght), Irrigated alpha amylase activity (IrrAA), Irrigated falling number (IrrFN), Adjusted Lactic Acid SRC (LAAdSRC), Lactic Acid SRC (LASRC), Sodium Carbonate SRC (NaSRC), Natural alpha amylase activity (NatAA), Natural falling number (NatFN), red or white seed color (SdClr), Softness Equivalence (SftEqv), Sucrose SRC (SucSRC), test weight (TstWt), and Water SRC (WatSRC).

**Figure 3:**
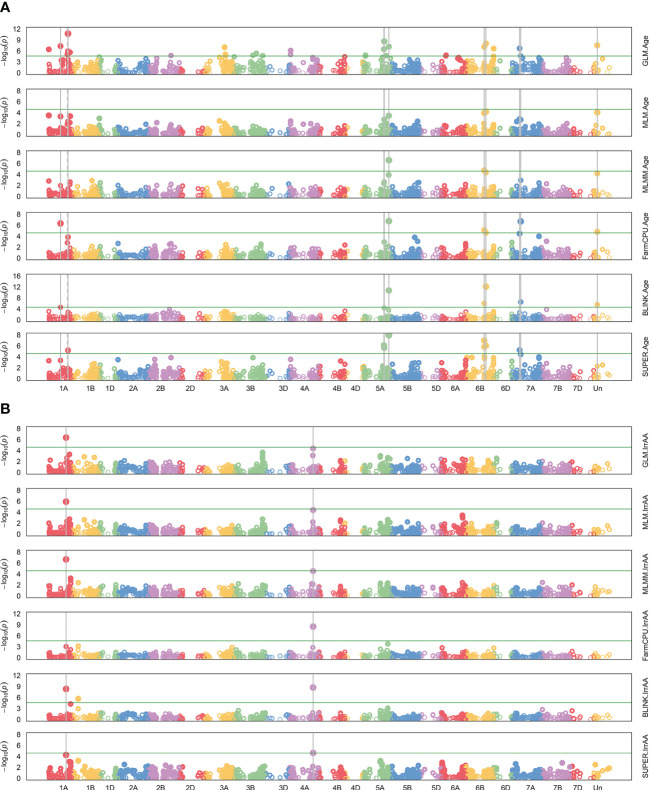
Manhattan plots for genome-wide association of all six models, GLM, MLM, MLMM, FarmCPU, BLINK, and SUPER. Model names are shown on the right. Negative log 10 of *p*-value for each marker on a chromosome are colored dots. Red horizontal line is the default, more stringent experiment-wise Bonferroni significance threshold in GAPIT3 of alpha = 0.01. Dashed gray vertical lines indicate two models significant for the same marker, and solid gray vertical lines, three or more. **(A)** Age of breeding line (Age) shown with many very significant markers across several models. **(B)** Irrigated alpha amylase activity (IrrAA) shown with markers on chromosome 1A and 4A with multiple models showing strong significance.

**Figure 4:**
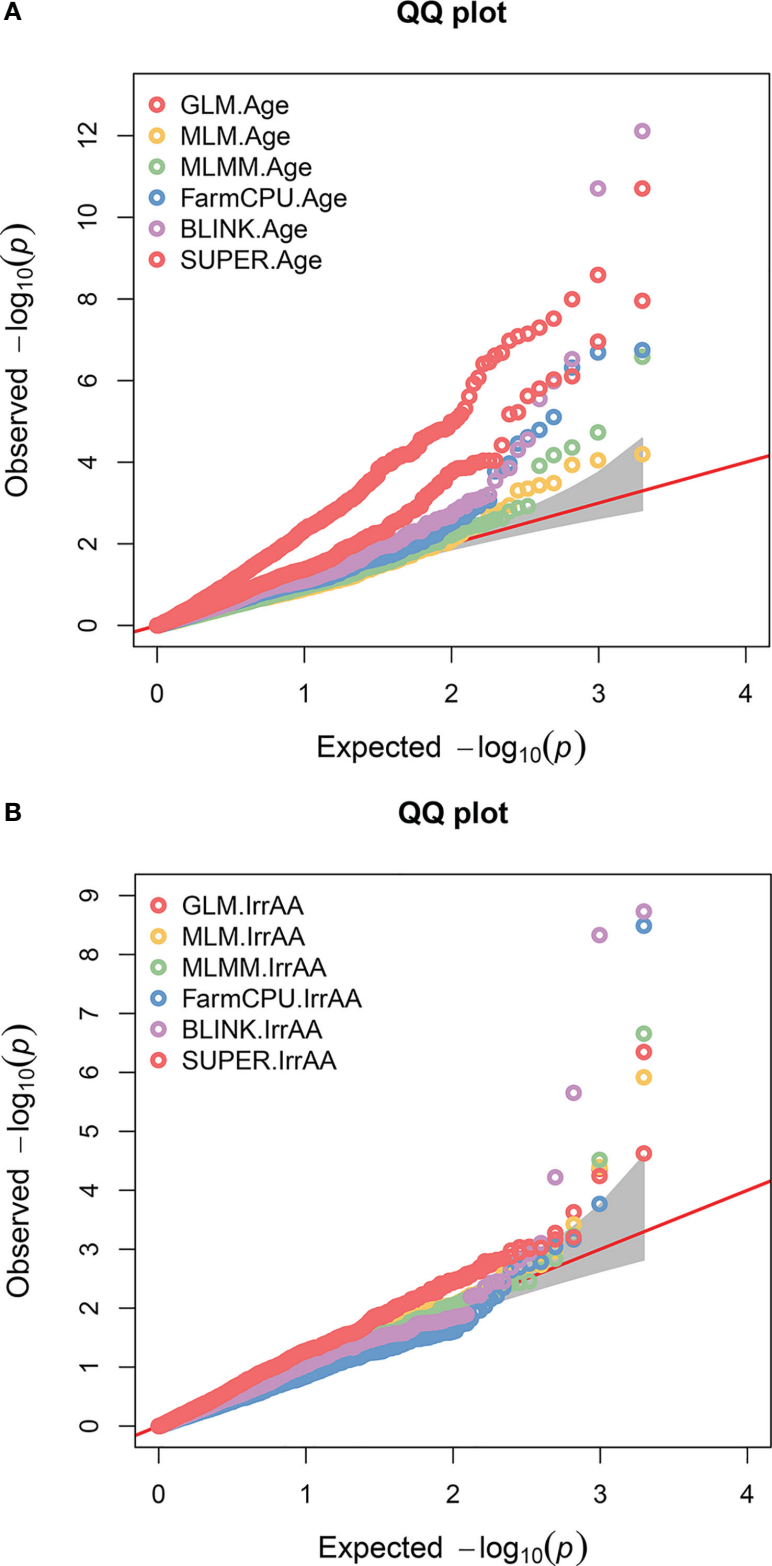
QQ plots for two genome-wide association traits. Colored circles represent the six different models (GLM, MLM, MLMM, FarmCPU, BLINK, and SUPER) tested for each trait. The red diagonal line indicates where observed and expected results would match. Gray shaded region is confidence interval and colored circles significantly above the line represent deviations that may be significantly associated with phenotype. **(A)** Age of breeding line (Age) shown with many potentially significant phenotype associations across several models but the majority of GLM appear too far above the line. **(B)** Irrigated alpha amylase activity (IrrAA) shown with fewer potentially strong significant associations to phenotype.

A GWAS for 22 agronomic, flour quality, and PHS traits with combined locations/years by BLUP using six models (Blink, FarmCPU, GLM, MLM, MLMM, and SUPER) was performed in GAPIT3 to test for model performance over diverse traits in a moderate-sized soft winter wheat population. See the R script in **File S2**. **File S3** contains BLUP estimated values. **File S4** contains HAPMAP markers with 1,978 markers mapped uniquely to a single genome (A, B, or D) for the 188 genotypes. The analysis uncovered 226 significant QTNs at 102 genomic regions across all 21 chromosomes and four unassigned regions and included 40 markers with multiple significant traits at 26 regions. **Figure 2** provides a visual representation of the QTN regions for all 22 traits while **Table 2** provides detailed information of the QTN including approximate regions in centiMorgans (cM), largest effect on the trait, smallest *p*-value for significance, and which markers and models were significant (**Supplemental Table S2**). **Figure 3** shows the stacked Manhattan plots for Age and IrrAA as examples of important traits showing only experiment-wise significant markers. **Figure 3** shows several locations where multiple models have the same marker significant at a more stringent experiment-wise Bonferroni cutoff of *p*-value <0.01 for Age and locations on chromosomes 1A and 4A for IrrAA. The QQ plot for Age showed some deviation from expected values for the GLM beyond the points at the end associated with phenotypic differences while the QQ plot for IrrAA shows all models within expected values except potential markers associated with phenotypic differences (**Figure 4**). Manhattan and QQ plots generated by GAPIT3 for each trait not in the main article are available along with histograms of the BLUP trait values as **File S5**. Measured traits IrrFN, FlProt, WatSRC, SucSRC, LASRC, LAADSRC, SftEqv, FlYld, and CkDia were all normally distributed by the Shapiro–Wilk normality test. However, the majority of test statistics fit well into their quartile values, *W* > 0.9, except ArtAA and NatAA with a limited range of values; Age, which is heavily skewed to newer lines; and Awns and SdClr, which are binary by classification (**Supplemental Table S1**). For inclusion as a QTN, the marker for the trait had to be significant by a 0.05 Bonferroni cutoff for at least two of the six models (**Supplemental Table S2**).

**Table 2:** All significant QTN regions including GWAS models and markers.

		Pos. (MBP^1^)		App. Loc (cM)^2^		Significant Models^4^		Smallest *p*-value^5^	Largest effect^6^	
QTN	Chrom	Start	Stop		Trait^3^	<0.05	<0.1			Marker
1A-1	1A	3	3	3	Age	G, M	S	**3.90E−07**	−22.74	wsnp_Ex_c12254_19575022
1A-2	1A	8	8	10	SftEqv	B, G, S		**1.07E−05**	−2.81	wsnp_CAP11_c710_458019
1A-3	1A	301	301	68	Age	B, F, G	M, S	**5.04E−08**	−29.97	wsnp_BE497361A_Ta_1_1
1A-4	1A	443	443	76	IrrAA	B, G, M, MM, S		**4.68E−09**	0.27	wsnp_JD_c2638_3555755
					NatAA	B, F, G, M, MM, S		**8.27E−10**	0.26	wsnp_JD_c2638_3555755
1A-5	1A	477	495	80–81	FlProt	G, S		**1.81E−05**	0.20	wsnp_Ex_c16592_25117132
					Age	F, G, S		**1.96E−11**	23.19	wsnp_Ex_c21336_30465618
					FlProt	G, S		**2.97E−06**	0.21	wsnp_Ex_c21336_30465618
					Hght	B, F, G, S		**4.28E−06**	−5.01	wsnp_Ra_c4664_8410628
1A-6	1A	501	501	90	FlYld	B, G		2.16E−04	−0.45	wsnp_Ra_c3270_6136601
1A-7	1A	530	530	104	NatAA	G, M		2.22E−04	0.10	wsnp_Ex_c43228_49605281
1A-8	1A	542	542	117	WatSRC	G, S		**4.45E−05**	−0.67	wsnp_Ex_c28900_37982485
					WatSRC	G, S		**4.45E−05**	0.67	wsnp_BE443588A_Ta_2_1
					NaSRC	F, G	S	3.01E−04	−0.82	wsnp_Ex_c26688_35918122
					WatSRC	G, S		**2.83E−05**	−0.68	wsnp_Ex_c26688_35918122
1B-1	1B	60	119	24–27	LASRC	G, S		1.93E−04	−3.74	wsnp_Ku_c4911_8795151
					LAAdSRC	B, F, G, M, MM, S		**1.75E−10**	6.91	wsnp_Ku_c16938_25916279
					LASRC	G, M, MM, S		**8.54E−09**	8.36	wsnp_Ku_c16938_25916279
					LAAdSRC	G, M, MM, S		**9.52E−07**	−6.90	wsnp_Ex_c20975_30093113
					LASRC	B, F, G, M, MM, S		**6.99E−12**	−8.36	wsnp_Ex_c20975_30093113a
1B-2	1B	143	144	30	CkTpGr	G, S		1.32E−04	−0.49	wsnp_BE399980B_Ta_2_1
					FlYld	G, MM, S	M	**1.41E−05**	−0.55	wsnp_BE399980B_Ta_2_1
					SucSRC	B, G	F	**1.25E−05**	2.00	wsnp_BE399980B_Ta_2_1
					FlYld	B, F, G, MM, S	M	**6.92E−07**	0.45	wsnp_Ku_c230_460618
1B-3	1B	382	382	39	NatAA	G, M		5.67E−05	−0.07	wsnp_Ex_c30805_39678077
1B-4	1B	464	464	48	Awns	B, F		**4.65E−06**	−0.22	wsnp_Ex_rep_c67584_66224379
1B-5	1B	563	563	77	NaSRC	B, F		**4.50E−06**	−0.59	wsnp_JD_c2636_3554874
1B-6	1B	646	646	111	SftEqv	B, S		**6.63E−08**	−1.22	wsnp_CAP11_c1902_1022408
1D-1	1D	8	9	14–21	LAAdSRC	G, S		1.14E−04	−4.72	wsnp_Ex_c1085_2078944
					LAAdSRC	G, S		1.55E−04	−4.90	wsnp_Ex_c1358_2600929
					LASRC	G, S		1.21E−04	−5.57	wsnp_Ex_c1358_2600929
					SucSRC	G, S		7.43E−05	−2.54	wsnp_Ex_c1358_2600929
					LASRC	G, S		7.03E−05	6.84	wsnp_Ex_c1358_2601510
					LAAdSRC	G, S		7.68E−05	5.17	wsnp_Ex_c1358_2602235
					LASRC	G, S		6.08E−05	5.86	wsnp_Ex_c1358_2602235
					SucSRC	G, S		**3.96E−05**	2.66	wsnp_Ex_c1358_2602235
1D-2	1D	408	408	89	LAAdSRC	B, S		**4.80E−11**	−5.16	wsnp_Ex_c1318_2520916
					LASRC	B, S		**1.33E−06**	3.99	wsnp_Ex_c6920_11929171
					LASRC	F, S		**1.78E−06**	−3.56	wsnp_Ex_c57601_59245380
					LAAdSRC	F, S		**1.24E−07**	4.37	wsnp_Ku_c5560_9853214
2A-1	2A	121	192	83–115	SdClr	F, M, MM	G	**3.50E−07**	0.51	wsnp_CAP12_c948_496702
2A-2	2A	192	192	115	NatAA	B, F		**1.67E−06**	0.08	wsnp_Ex_c2536_4728768
					SdClr	B, F, G, S	M, MM	**1.09E−05**	−0.24	wsnp_Ex_c2536_4728768
2A-3	2A	714	714	170	NaSRC	B, F, MM	M	**5.91E−06**	−1.58	wsnp_JD_rep_c64440_41093162
					SucSRC	G, M, MM, S		7.54E−05	−3.64	wsnp_JD_rep_c64440_41093162
2A-4	2A	718	718	174	SdClr	G, M, MM		**4.30E−06**	0.15	wsnp_BE445431A_Td_2_2
					NaSRC	B, F		**2.11E−06**	1.03	wsnp_BE445431A_Td_2_1
2A-5	2A	771	771	214	SucSRC	B, F		**2.67E−06**	1.23	wsnp_Ex_rep_c108004_91402649
2B-1	2B	25	25	20	Hght	B, F		**4.67E−07**	−2.82	wsnp_Ex_c2388_4476302
2B-2	2B	103	103	117	LAAdSRC	B, S	G	**1.05E−07**	−3.04	wsnp_Ex_c5239_9272511
2B-3	2B	147	147	106	SdClr	B, M, MM, S	G	**2.74E−05**	−0.45	wsnp_CAP12_rep_c4678_2134259
2B-4	2B	389	443	147–147	LAAdSRC	F, G, S		**2.87E−06**	−5.71	wsnp_Ex_c22693_31898036
					LAAdSRC	G, S		**7.62E−06**	−5.48	wsnp_Ex_c9133_15199135
					LAAdSRC	G, S		**7.62E−06**	−5.48	wsnp_Ex_c21532_30680512
					LAAdSRC	G, S		**7.62E−06**	−5.48	wsnp_Ex_c36002_44045355
					LAAdSRC	G, S		**7.31E−06**	5.59	wsnp_Ex_c28243_37383894
2B-5	2B	579	579	163	Age	G, S		**1.89E−05**	15.15	wsnp_JD_rep_c67103_42432235
2D-1	2D	15	15	12	FlYld	G, S		1.30E−03	0.38	wsnp_CAP12_c455_248396
					WatSRC	G, S		1.23E−03	−0.50	wsnp_CAP12_c455_248396
2D-2	2D	18	18	12	TstWt	G, M, MM, S	B, F	7.26E−04	0.76	wsnp_Ex_c1408_2704736
2D-3	2D	67	67	90	ArtAA	B, F, G, M, MM, S		5.89E−04	−0.04	wsnp_BM140538D_Ta_2_1
					ArtFN	B, F	G, M, MM	1.91E−03	41.18	wsnp_BM140538D_Ta_2_1
2D-4	2D	81	81	101	NatAA	B, F, G, M, MM, S		**3.38E−05**	0.03	wsnp_BE444144D_Ta_1_1
2D-5	2D	514	514	165	LAAdSRC	G, S		6.02E−05	−5.24	wsnp_Ex_c26281_35525999
2D-6	2D	617	617	172	SdClr	B, S		7.50E−04	NA	wsnp_Ex_c21593_30744815
3A-1	3A	138	138	57	Hght	G, S		2.25E−04	3.22	wsnp_Ex_c25653_34914467
3A-2	3A	424	505	63–64	CkTpGr	G, S	F	9.81E−05	0.38	wsnp_Ra_c19979_29215858
					FlProt	G, S		**4.75E−05**	−0.20	wsnp_Ra_c19979_29215858
					CkDia	G, S		1.67E−04	−0.18	wsnp_Ex_rep_c67349_65914945
					CkTpGr	B, G, S		**7.57E−06**	−0.35	wsnp_Ex_rep_c67349_65914945
					FlProt	B, F, G, S		**1.31E−06**	0.23	wsnp_Ex_rep_c67349_65914945
					CkDia	B, G, S		**4.41E−06**	−0.25	wsnp_Ex_c3571_6530739
					CkDia	G, S		**1.62E−05**	−0.28	wsnp_BG263758A_Ta_1_1
					FlProt	G, S		1.73E−04	0.32	wsnp_BG263758A_Ta_1_1
3A-3	3A	508	508	76	Hght	B, G, S		**5.23E−06**	5.05	wsnp_Ex_c12850_20377830
3A-4	3A	534	534	83	LASRC	G, S		**1.95E−05**	4.20	wsnp_Ex_c15269_23491104
3A-5	3A	571	571	109	SftEqv	B, S	F, G	**1.49E−05**	−0.82	wsnp_Ex_rep_c66685_65003625
3A-6	3A	646	652	118–123	LAAdSRC	B, F, G, S		**5.35E−09**	−5.11	wsnp_Ex_c18747_27625264
					LASRC	B, F, G, S	M, MM	**9.15E−08**	−5.70	wsnp_Ex_c18747_27625264
					SdClr	F, G		**5.11E−07**	−0.12	wsnp_Ex_c18747_27625264
					LAAdSRC	G, S		1.45E−04	3.82	wsnp_Ra_c16053_24607526
3A-7	3A	659	659	123	Awns	B, F, G, S		**2.21E−07**	0.12	wsnp_Ex_c14202_22145805
					Awns	G, S		1.09E−04	−0.11	wsnp_Ex_c14202_22145136
3A-8	3A	714	714	146	SdClr	B, F, G, M, MM, S		**5.13E−10**	−0.17	wsnp_CAP8_c6939_3242530
3B-1	3B	60	60	61	WatSRC	B, F, S		**2.38E−05**	0.42	wsnp_Ex_c2820_5215394
3B-2	3B	259	259	72	FlProt	G, S		1.31E−04	−0.21	wsnp_BE591466B_Ta_2_1
3B-3	3B	414	414	74	FlYld	G, S		**2.17E−06**	−12.32	wsnp_BE489326B_Ta_2_2
3B-4	3B	486	486	84	Age	G, S		**1.62E−05**	−12.32	wsnp_Ex_c14462_22457559
3B-5	3B	618	618	95	NaSRC	B, F, G, MM, S	M	**1.07E−08**	−0.85	wsnp_JD_c5643_6802088
					NaSRC	G, MM, S	M	**3.18E−05**	−0.85	wsnp_JD_c5643_6802211
3D-1	3D	44	44	13	LAAdSRC	B, S		1.44E−03	−2.42	wsnp_Ex_c4661_8344663
3D-2	3D	119	119	145	FlProt	B, F, G, S	MM	**4.95E−05**	0.22	wsnp_JD_c12087_12411036
3D-3	3D	600	600	18	NaSRC	B, F, G, MM, S	M	**2.95E−05**	−0.51	wsnp_Ku_c13204_21105694
3D-4	3D	603	604	18	Hght	G, S		2.71E−04	−5.56	wsnp_Ra_c5433_9630495
					Hght	G, S		1.68E−03	4.63	wsnp_Ra_c7174_12417331
					Hght	G, S		7.00E−04	5.25	wsnp_BE444579D_Ta_2_1
					Hght	G, S		1.68E−03	4.63	wsnp_BE444579D_Ta_2_2
					Hght	G, S		2.71E−04	−5.56	wsnp_BE444579D_Ta_2_3
3D-5	3D	611	611	196	HD	B, G	M, MM	4.64E−04	−1.09	wsnp_Ku_c2249_4335279
4A-1	4A	30	30	182	Age	G, S		**8.48E−07**	−21.54	wsnp_CAP8_c1013_646748
4A-2	4A	102	116	151–156	LAAdSRC	G, S	B	**3.77E−05**	4.29	wsnp_Ex_c6044_10590220
					LAAdSRC	G, S		**2.36E−05**	4.59	wsnp_JD_c14769_14413046
					LAAdSRC	G, S		1.55E−04	−5.28	wsnp_Ex_c35839_43909849
					LAAdSRC	G, S		1.55E−04	5.28	wsnp_Ex_c14529_22547438
4A-3	4A	596	596	122	SdClr	B, F, G, M, MM, S		**1.61E−05**	0.14	wsnp_Ex_c21165_30292808
					SdClr	G, M, MM		**4.80E−05**	−0.13	wsnp_Ex_c4068_7351806
					SdClr	G, M, MM		**3.93E−05**	0.14	wsnp_Ex_c17338_26018247
4A-4	4A	604	604	113	ArtAA	B, F	G, M, MM	3.92E−04	0.01	wsnp_Ex_rep_c66324_64493429
					IrrAA	B, F, G, M, MM, S		**1.86E−09**	0.04	wsnp_Ex_rep_c66324_64493429
					IrrFN	B, F, G, M, MM, S		7.74E−05	−13.76	wsnp_Ex_rep_c66324_64493429
4B-1	4B	13	13	15	SdClr	F, G		**1.36E−05**	−0.07	wsnp_Ku_c7180_12403155
4B-2	4B	564	564	72	Age	G, S		5.87E−04	−10.71	wsnp_Ex_c30581_39482788
4B-3	4B	573	573	72	ArtAA	B, F, G, M, MM, S		5.29E−05	0.06	wsnp_Ku_c17721_26864251
					ArtFN	B, F, G, M, MM, S		3.16E−04	−53.00	wsnp_Ku_c17721_26864251
4B-4	4B	645	672	39–125	Hght	F, G		**2.47E−05**	−2.98	wsnp_Ex_c29867_38850724
					Hght	B, G	S	**7.15E−05**	2.92	wsnp_Ku_c5566_9864771
					Hght	G, MM, S	M	1.86E−04	−3.04	wsnp_JD_c9484_10319946
4D-1	4D	2	2	8	NatAA	G, M		2.15E−03	0.11	wsnp_Ex_c23850_33089300
4D-2	4D	455	455	53	ArtAA	B, F, MM	G, M	4.72E−03	0.01	wsnp_BE497160D_Ta_2_1
					HD	G, S	B	3.53E−03	−0.66	wsnp_BE497160D_Ta_2_1
5A-1	5A	52	66	153	SftEqv	G, S		6.21E−05	1.13	wsnp_Ku_c1102_2211433
					SftEqv	G, S		**3.62E−05**	−1.16	wsnp_Ra_rep_c105791_89683548
					SftEqv	G, S		5.19E−05	1.14	wsnp_Ex_rep_c67636_66293429
					SftEqv	G, S		5.19E−05	1.14	wsnp_JD_c7404_8500079
					SftEqv	G, S		**3.62E−05**	−1.16	wsnp_Ex_c23509_32746909
5A-2	5A	104	109	150	SftEqv	B, G, S		**4.70E−06**	−1.29	wsnp_Ex_c130_259533
5A-3	5A	109	109	150	LAAdSRC	F, G		**1.42E−05**	3.21	wsnp_Ra_c18459_27525981
					SftEqv	G, S		**4.70E−06**	1.29	wsnp_Ra_c18459_27525981
5A-4	5A	158	158	140	SftEqv	B, F, G, S		**4.98E−06**	1.30	wsnp_BE444644A_Ta_2_1
5A-5	5A	512	520	99–109	Age	G, S		**3.53E−07**	−14.07	wsnp_Ex_c49211_53875575
					FlProt	G, S		**4.85E−05**	−0.15	wsnp_Ex_c49211_53875575
					Age	B, G, S		**2.57E−09**	19.09	wsnp_Ex_c11322_18287597
					FlProt	B, F, G, S		**4.08E−07**	0.21	wsnp_Ex_c11322_18287597
5A-6	5A	574	574	80	CkDia	B, F, G, S		**8.07E−08**	0.17	wsnp_RFL_Contig3739_3996324
					NaSRC	B, F, G, S		**2.89E−08**	−0.81	wsnp_RFL_Contig3739_3996324
					SucSRC	B, F, G, S		**1.62E−06**	−2.08	wsnp_RFL_Contig3739_3996324
					WatSRC	B, G		**1.63E−07**	−0.69	wsnp_RFL_Contig3739_3996324
5A-7	5A	634	637	46–47	Age	B, MM		1.22E−04	−14.84	wsnp_Ex_c16715_25264080
					Age	B, F, G, M, MM, S		**1.96E−11**	−17.59	wsnp_Ex_c1880_3545329
5A-8	5A	708	708	1	Awns	F, G		6.71E−05	0.12	wsnp_Ex_c2171_4072995
5B-1	5B	21	21	204	TstWt	B, F, G	MM	8.55E−05	0.51	wsnp_BE499835B_Ta_2_5
5D-1	5D	58	58	143	SdClr	G, M, MM		2.45E−04	0.45	wsnp_CAP7_c3386_1586636
5D-2	5D	342	342	126	SdClr	G, M, MM		2.45E−04	0.45	wsnp_Ex_c23358_32602488
					SdClr	G, M, MM		2.94E−04	0.25	wsnp_Ex_c23358_32602315
6A-1	6A	4	4	1	LAAdSRC	G, S		2.17E−04	−8.03	wsnp_RFL_Contig3512_3672726
					LASRC	G, S		**2.09E−05**	−9.82	wsnp_RFL_Contig3512_3672726
6A-2	6A	13	13	25	FlYld	B, G, S	F, M, MM	**3.93E−06**	−0.52	wsnp_Ex_rep_c68165_66935148
					FlYld	G, S	F	**3.31E−05**	0.50	wsnp_Ex_rep_c68165_66935041
					FlYld	G, S	F, MM	**4.79E−05**	−0.51	wsnp_Ex_c3530_6459643
6A-3	6A	105	115	89–90	CkDia	G, S		**2.80E−06**	−0.18	wsnp_Ra_c61979_62214892
					CkTpGr	G, S	F	1.15E−04	−0.29	wsnp_Ra_c61979_62214892
					FlProt	G, S		**2.17E−07**	0.23	wsnp_Ra_c61979_62214892
					Hght	B, F, G, S		**1.99E−06**	4.30	wsnp_Ra_c61979_62214892
					CkDia	B, F, G, S		**2.07E−07**	0.18	wsnp_Ex_c17692_26437459
					FlProt	B, F, G, S		**2.28E−08**	−0.24	wsnp_Ex_c17692_26437459
					Hght	G, S		**3.25E−06**	−4.21	wsnp_Ex_c17692_26437459
					CkDia	G, S		**2.12E−06**	0.18	wsnp_Ra_c16745_25482384
					FlProt	G, S		**6.32E−08**	−0.24	wsnp_Ra_c16745_25482384
					Hght	G, S		**3.25E−06**	−4.21	wsnp_Ra_c16745_25482384
6A-4	6A	448	448	98	FlProt	G, S		**7.54E−06**	0.22	wsnp_BF202329A_Ta_2_2
					FlProt	G, S		**7.54E−06**	0.22	wsnp_BM134512A_Ta_2_2
6A-5	6A	494	494	114	CkDia	G, S		**1.17E−05**	−0.21	wsnp_Ex_c16480_24986490
					FlProt	G, S		**9.45E−06**	0.23	wsnp_Ex_c16480_24986490
					Hght	G, S		6.07E−05	4.24	wsnp_Ex_c16480_24986490
					SucSRC	B, F, G		**2.03E−07**	2.56	wsnp_Ex_c16480_24986490
6B-1	6B	9	9	1	LASRC	G, S		**2.79E−05**	−9.96	wsnp_Ku_c4446_8062906
6B-2	6B	13	13	5	TstWt	B, F, G	MM	2.40E−04	1.12	wsnp_CD453605B_Ta_2_6
6B-3	6B	27	27	22	WatSRC	F, G, S		6.24E−05	0.72	wsnp_Ra_c20409_29673950
6B-4	6B	471	471	82	Age	B, F, G, M, MM, S		**8.18E−08**	21.18	wsnp_Ra_c14498_22667649
					HD	B, G	F, S	**1.03E−06**	1.07	wsnp_Ra_c14498_22667649
6B-5	6B	515	515	83	Age	B, F, G, M, MM, S		**7.68E−13**	−47.17	wsnp_BM134512B_Ta_2_1
6B-6	6B	576	599	95–97	NaSRC	B, F, G, S	MM	**1.35E−10**	−0.99	wsnp_CAP11_c166_172556
					WatSRC	B, G	S	**5.87E−06**	−0.58	wsnp_CAP11_c166_172556
					CkTpGr	B, F, G, S		**1.22E−05**	−0.48	wsnp_BE490147B_Ta_2_1
					FlYld	B, F, G		**1.77E−05**	−0.77	wsnp_BE490147B_Ta_2_1
					WatSRC	G, M, MM		**1.87E−06**	1.15	wsnp_BE490147B_Ta_2_1
					CkTpGr	F, G, S		**3.75E−05**	−0.48	wsnp_BE496986B_Ta_2_2
					FlYld	F, G		**4.31E−05**	−0.77	wsnp_BE496986B_Ta_2_2
					WatSRC	G, M, MM		**1.87E−06**	1.15	wsnp_BE496986B_Ta_2_2
					CkTpGr	F, G, S		**3.75E−05**	−0.48	wsnp_BE497701B_Ta_2_1
					FlYld	F, G		**4.31E−05**	−0.77	wsnp_BE497701B_Ta_2_1
					WatSRC	G, M, MM		**1.87E−06**	1.15	wsnp_BE497701B_Ta_2_1
					CkDia	F, G		**4.70E−05**	−0.24	wsnp_Ex_c23010_32232119
					CkTpGr	G, S	F	1.06E−04	−0.45	wsnp_Ex_c23010_32232119
					WatSRC	G, S	M, MM	**3.47E−06**	1.12	wsnp_Ex_c23010_32232119
6B-7	6B	695	695	100	Awns	B, F, G, M, MM, S		**1.76E−10**	0.26	wsnp_Ex_c8963_14948293
6B-8	6B	709	712	150–151	Age	G, S		**2.77E−05**	−16.64	wsnp_CAP11_c949_571671
					CkDia	B, G		**1.88E−06**	0.20	wsnp_CAP11_c949_571671
					WatSRC	B, G, MM, S	M	**8.64E−09**	−0.89	wsnp_CAP11_c949_571671
					FlProt	G, S		**1.43E−06**	0.22	wsnp_Ex_c54772_57527387
					Hght	G, S		**4.00E−05**	−2.81	wsnp_Ex_c54772_57528275
					FlProt	G, S		**1.63E−06**	0.22	wsnp_Ex_rep_c83634_77351566
					CkTpGr	B, F, G		**4.06E−05**	0.28	wsnp_RFL_Contig3211_3221207
					FlProt	G, S		**5.19E−06**	−0.18	wsnp_RFL_Contig3211_3221207
					Hght	G, S		**2.00E−05**	−3.19	wsnp_RFL_Contig3211_3221207
6D-1	6D	67	67	49	NatAA	B, G, M, S		5.44E−05	0.03	wsnp_Ra_c4330_7871129
					NatFN	B, F, G, M, MM		3.66E−04	−19.81	wsnp_Ra_c4330_7871129
					NatAA	B, F, G, M, MM, S		**6.78E−06**	0.03	wsnp_Ex_c1249_2399894
					NatFN	B, F, G, M, MM		1.26E−04	−21.22	wsnp_Ex_c1249_2399894
6D-2	6D	459	459	10	Age	F, S		6.36E−04	6.38	wsnp_Ex_c30754_39633791
					Awns	F, G, MM, S		**3.71E−05**	−0.15	wsnp_Ex_c30754_39633791
6D-3	6D	469	469	151	HD	G, MM	M	5.50E−04	−1.87	wsnp_CAP7_c1735_859744
7A-1	7A	77	78	45	Awns	G, S		**3.55E−05**	0.11	wsnp_CAP11_c1182_686503
					Awns	B, F, G	M, MM	**2.50E−09**	0.14	wsnp_Ex_c41150_48040078
7A-2	7A	169	169	66	LAAdSRC	B, F		1.49E−04	−5.97	wsnp_BE498209A_Ta_2_1
7A-3	7A	182	211	66	Age	F, G, S		**2.08E−07**	22.58	wsnp_Ex_c21068_30195276
					Age	B, F, G, S		**2.04E−07**	54.82	wsnp_BE591002A_Ta_2_3
7A-4	7A	594	594	100	SftEqv	F, G	S	**3.67E−05**	−1.64	wsnp_Ex_c19214_28132186
7A-5	7A	676	676	134	Age	G, S		6.84E−05	−12.09	wsnp_CAP7_c1321_664478
					Age	F, G, S		1.05E−04	11.76	wsnp_CAP7_c1321_664480
7A-6	7A	717	717	171	Hght	B, G, S		**1.13E−06**	−3.18	wsnp_Ra_c7112_12318340
7B-1	7B	264	264	47	ArtAA	B, F, MM	G, M, S	1.75E−04	0.05	wsnp_RFL_Contig1735_856501
7B-2	7B	471	471	58	SdClr	M, MM	G	2.45E−04	−0.45	wsnp_BE398417B_Ta_2_1
7B-3	7B	570	571	66	Awns	G, M, MM, S		**1.99E−06**	0.22	wsnp_BF483648B_Ta_2_1
					Awns	G, M, MM, S		**1.99E−06**	0.22	wsnp_Ex_c15458_23737002
					Awns	G, M, MM, S		**1.99E−06**	−0.22	wsnp_Ku_c21412_31166369
7B-4	7B	644	644	90	SucSRC	G, S		**2.79E−05**	−1.69	wsnp_Ex_c10500_17163956
					SucSRC	G, S		**2.79E−05**	−1.69	wsnp_Ku_c854_1768346
					SucSRC	B, G	F	**2.75E−05**	−1.58	wsnp_Ex_c3309_6096114
					SucSRC	G, S		**2.79E−05**	−1.69	wsnp_Ku_c854_1768062
7D-1	7D	614	614	3	LASRC	G, S		1.48E−03	8.28	wsnp_JD_c5853_7011562
Un-1	Un	24	24	–	LASRC	B, F, S		**5.24E−06**	−2.84	wsnp_Ex_c1668_3168723
Un-2	Un	57	57	–	Hght	G, M, MM	S	1.00E−03	−13.38	wsnp_BF482269B_Ta_1_1
Un-3	Un	87	87	–	Age	B, F, G, M, MM		**3.03E−08**	−18.99	wsnp_Ex_c758_1488368
Un-4	Un	222	222	–	FlProt	G, S		**2.39E−05**	0.21	wsnp_Ex_c7316_12552186
					Hght	G, S		**1**.31E−04	3.43	wsnp_Ex_c7316_12552186

1: MBP, Mega base pairs.

2: Approximate genetic distances in cM from Wang et al. (2014b) **Table S6**, if available.

3: For descriptions, see **Table 1**.

4: Models significant for Bonferroni threshold by chromosome in **Table S1** for indicated alpha where: B = Blink, F = FarmCPU, G = GLM, M = MLM, MM = MLMM, S = SUPER.

5: Most significant p-value for all models, bold indicates experiment-wise significance considering all markers.

6: Greatest potential change in trait value predicted by GAPIT3 models except SUPER and BLINK, which did not report effect.

Five regions on chromosomes 1A, 5A, 6B, and 6D overlapped Age and agronomic or flour quality traits including Hght, HD, Awns, WatSRC, FlProt, CkDia, and CkTpGr and all but Hght and CkTpGr shared common markers with Age (**Figure 2**, **Table 2**). Quantitative trait nucleotide 1A-1, 80-81 estimated cM region with Age, FlProt, and Hght had *p*-values of 2 × 10^−5^ to 2 × 10^−6^. Quantitative trait nucleotide 5A-5, 99-109 estimated cM region with Age and FlProt had *p*-values of 5 × 10^−5^ to 3 × 10^−9^. Quantitative trait nucleotide 6B-4, 82 estimated cM region with Age and HD had *p*-values of 1 × 10^−6^ to 8 × 10^−8^. Quantitative trait nucleotide 6B-8, 150-151 estimated cM region with Age, CkDia, WatSRC, FlProt, Hght, and CkTpGr had *p*-values of 2 × 10^−5^ to 9 × 10^−9^. Quantitative trait nucleotide 6D-2, 10 estimated cM region with Age and Awns had *p*-values of 6 × 10^−4^ to 4 × 10^−5^. These regions indicated where potential trait changes over time observed by Pearson’s correlation and PCA may have occurred. For identical markers, the FlProt effect was in the same direction as Age three times, while HD and WatSRC, once. Conversely, Awns and CkDia effects were the opposite of Age where increased Age indicated older breeding lines (**Table 2**).

Regions of PHS were separate from other traits except for co-located or closely located groups to SdClr on chromosomes 2A and 4A or HD and ArtAA on chromosome 4D. Seed color is QTN 2A-1 at an estimated 83–115 cM and a *p*-value of 3 × 10^−7^ while SdClr and NatAA are QTN 2A-2 at an estimated 115 cM and *p*-values of 1 × 10^−5^ to 2 × 10^−6^. Quantitative trait nucleotide 4A-3 is at an estimated 122 cM and *p*-values of 2 × 10^−5^ to 5 × 10^−5^ while QTN 4A-4 is ArtAA, IrrAA, and IrrFN at an estimated 113 cM with *p*-values of 4 × 10^−4^ to 2 × 10^−9^. Quantitative trait nucleotide 4D-4 is at an estimated 53 cM that includes ArtAA and HD with *p*-values of 4 × 10^−3^ to 5 × 10^−3^. Multiple measures of PHS co-located on chromosomes 1A, 2D, 4A, 4B, and 6D. Quantitative trait nucleotide 1A-4 is at an estimated 76 cM for IrrAA and NatAA with *p*-values of 5 × 10^−9^ to 8 × 10^−10^. Quantitative trait nucleotide 2D-3 is at an estimated 90 cM for ArtAA and ArtFN with *p*-values of 2 × 10^−3^ to 6 × 10^−4^. Quantitative trait nucleotide 4A-4 is described above. Quantitative trait nucleotide 4B-3 is at an estimated 72 cM for ArtAA and ArtFN with *p*-values of 3 × 10^−4^ to 5 × 10^−5^. Quantitative trait nucleotide 6D-1 is at an estimated 49 cM for NatAA and NatFN with *p*-values of 1 × 10^−4^ to 7 × 10^−7^. Only one region on chromosome 4A (QTN 4A-4, described above) had an overlap between ArtAA and irrigated PHS measures while irrigated and natural PHS measures were more often co-located. Multiple flour quality traits co-located on chromosomes 1A, 1B, 1D, 2A, 2D, 3A, 5A, 6A, and 6B. Quantitative trait nucleotide 1A-5 was described above. Quantitative trait nucleotide 1A-8 is at an estimated 117 cM including WatSRC and NaSRC with *p*-values from 3 × 10^−4^ to 4 × 10^−5^. Quantitative trait nucleotide 1B-1 is at an estimated 24–27 cM including LASRC and LAAdSRC with *p*-values from 2 × 10^−4^ to 7 × 10^−12^. Quantitative trait nucleotide 1B-2 is at an estimated 30 cM including CkTpGr, FlYld, and SucSRC with *p*-values of 1 × 10^−4^ to 7 × 10^−7^. Quantitative trait nucleotide 1D-1 is at an estimated 14-21 cM including LASRC, LAAdSRC, and SucSRC with *p*-values of 1 × 10^−4^ to 8 × 10^−5^. Quantitative trait nucleotide 1D-2 is at an estimated 89 cM and includes LASRC and LAdSRC with *p*-values of 1 × 10^−6^ to 5 × 10^−11^. Quantitative trait nucleotide 2A-3 is at an estimated 170 cM and includes NaSRC and SucSRC with *p*-values of 8 × 10^−5^ to 6 × 10^−6^. Quantitative trait nucleotide 2D-1 is at an estimated 12 cM and includes FlYld and WatSRC with *p*-values of 1 × 10^−3^. Quantitative trait nucleotide 3A-2 is at an estimated 63–64 cM and includes CkTpGr, CkDia, and FlProt with *p*-values of 2 × 10^−4^ to 8 × 10^−6^. Quantitative trait nucleotide 3A-6 is at an estimated 123 cM including LAAdSRC, LASRC, and SdClr with *p*-values of 1 × 10^−4^ to 5 × 10^−9^. Quantitative trait nucleotide 5A-3 is at an estimated 150 cM and includes LAAdSRC and SftEqv with *p*-values of 1 × 10^−5^ to 5 × 10^−6^. Quantitative trait nucleotide 5A-6 is at an estimated 80 cM and includes CkDia, NaSRC, SucSRC, and WatSRC with *p*-values of 2 × 10^−6^ to 8 × 10^−8^. Quantitative trait nucleotide 6A-3 is at an estimated 89–90 cM and includes CkDia, CkTpGr, FlProt, and Hght with *p*-values of 1 × 10^−4^ to 6 × 10^−8^. Quantitative trait nucleotide 6A-5 is at an estimated 114 cM and includes CkDia, FlProt, Hght, and SucSRC with *p*-values of 1 × 10^−5^ to 9 × 10^−6^. Quantitative trait nucleotide 6B-6 is at an estimated 95–97 cM and includes NaSRC, WatSRC, CkTpGr, CkDia, and FlYld with *p*-values of 1 × 10^−4^ to 1 × 10^−10^. Quantitative trait nucleotide 6B-8 is described above with overlap in Age (**Figure 2**, **Table 2**).

## 4 Discussion

The 188-member population of historically diverse soft winter wheat breeding lines was chosen for increased marker and trait diversity compared to elite breeding lines to maximize trait differences in the population. It has a rich history of genetic diversity, which could contain useful genes for PHS and flour quality trait enhancement that may have been bred out of elite lines selected only for limited agronomic traits such as yield and a few resistance genes. Population structure is often quite evident in wheat including the present population, which was previously compared to a larger elite wheat breeding population. Using Structure v 2.2, Cabrera et al. (2014) found five subpopulations of the historic diversity lines compared to six in a larger set of 449 elite parent lines. Three of the 5 historic diversity sub-populations separated mostly by Age, reported as release year. Wright’s fixation index between sub-groups for the historic diversity lines ranged from 0.178 to 0.485 while they ranged from 0.212 to 0.408 for the elite population (Cabrera et al., 2014). Diversity between the historic and much larger elite line set was found to be similar, and the historic diversity population had moderate structure due to recent and historic breeding history as well as red versus white seed color (Cabrera et al., 2014).

PHS is most often measured by FN or AA (Vetch et al., 2019; Patwa and Penning, 2020). The developed micro assay for alpha amylase activity showed strong correlation to the original method in a subset of 20 samples tested in three separate years. Both AA and FN were used in this study although more QTNs were obtained using AA (11) than FN (4). PHS only occurs when conditions such as high humidity or extensive rainfall occur at spike maturity (Vetch et al., 2019; Patwa and Penning, 2020). In addition to waiting for the correct conditions to occur in the field, NatAA and NatFN, spikes were removed and subjected to the correct conditions in a growth chamber, ArtAA and ArtFN, or once all plants reached maturity, they were subjected to overhead water sprinklers to simulate rainfall, IrrAA and IrrFN. Sixty-five potential regions affecting PHS resistance and flour quality were found including a highly significant region for PHS resistance on chromosome 4A and multiple regions for flour quality on chromosomes 6A and 6B (**Figure 2**, **Table 2**). Another goal was to observe if selective breeding of a few traits might affect unselected traits in the same chromosomal region. PHS measures showed no correlation to Age by Pearson’s correlation, PCA, or GWAS overlapping only with HD on chromosome 4D at 455 MBP. Thus, PHS resistance does not appear selected over time and could be improved with minimal impact to agronomic traits while providing resistance to an abiotic stress related to climate change. Some PHS resistance has been tied to dormancy genes or areas, so it is not unusual for PHS measures and HD, which can be affected by dormancy, to be associated (Nakamura et al., 2011; Martinez et al., 2016; Torada et al., 2016). Shorter et al. (2005) found that maturity could impact PHS depending on when a rain event occurred. The relationship between seed dormancy, HD, and PHS resistance is complicated. The rice gene, *Ghd7*, has been linked to pleotropic effects including seed dormancy and HD as well as other effects through the ratio of gibberellins and abscisic acid (Hu et al., 2021). A previous study in rice found a short locus on chromosome 3 for HD, and seed dormancy could be separated into two loci: one for HD, *Hd8*, and one for seed dormancy, *Sdr1*, through fine mapping of recombination events within the region (Takeuchi et al., 2003). In barley, two different *HvNCED* genes that catalyze a key step in abscisic acid synthesis were found to be differentially expressed based on seed developmental age or water imbibition (Chono et al., 2006). Seed dormancy has been shown to have a large effect on PHS resistance but is not the only factor involved (DePauw and McCaig, 1991). Although the literature suggests and PCA indicated some association between HD and AA measures of PHS, only ArtAA was correlated significantly with HD by Pearson’s correlation and had a single co-localization in GWAS. Age was positively correlated with Hght, FlProt, and HD. Examination of trait positive or negative effects at individual markers in the GWAS indicated older breeding lines headed later and had higher flour protein content and greater water absorption but were less likely to have awns and had a smaller CkDia. Quantitative trait nucleotides 1A-5 and 6B-8 had Age, FlProt, and Hght co-localizations while 5A-5 had Age and FlProt co-localizations and 6B-4 had Age and HD co-localizations indicating regions that may have had two agronomic and one flour quality trait selected for over time. Quantitative trait nucleotide 6B-8 also had co-localizations of Age with WatSRC, CkDia, and CkTpGr, indicating the potential impact of other flour quality traits over time. Using **Supplementary Data** from Wang et al. (2014b), approximate regions in cM were calculated to compare with other studies. Five Hght QTN regions, 2B-1 (Ppd-B1), 3A-3 (qRht.3A), 4B-4 (Rht-B1), and 6A-3 and 6A-5 (Rht24), were 0.7–8.8 cM from other quantitative trait loci (QTL) studies of 410 European winter wheat varieties, a fine mapping set of 110 European winter wheat varieties, and four F2 mapping populations (Würschum et al., 2015; Zhou et al., 2016; Würschum et al., 2017). Markers are listed in **Table 3**. However, no regions were found on 1D, 2A, 2D, 4D, 5A, 5B, and 7D but additional QTN regions were found on 1A-5, 3D-4, 6B-8, 7A-6, and two unlinked markers. Differences could be attributed to population differences between US and European wheat varieties.

**Table 3:** Matches to QTL regions previously found in other studies.

Trait	QTN Marker	Chrom	MBP	cM^1^	Gene/QTL	cM	Reference
PHS	wsnp_JD_c2638_3555755	1A	442	NA^#^	QPHS.wsu-1A.1	82	Martinez et al., 2018
PHS	wsnp_Ra_c2895_5488879*	1A	462	76	QPHS.wsu-1A.1	82	Martinez et al., 2018
PHS	wsnp_Ex_c30805_39678077	1B	382	39.4	QPHS.wsu-1B.1	31	Martinez et al., 2018
PHS	wsnp_Ex_c30805_39678077	1B	382	39.4	wPt-666564	33.5	Kulwal et al., 2012
PHS	wsnp_Ex_rep_c66324_64493429	4A	604	113	wPt-730913	93.1	Kulwal et al., 2012
PHS	wsnp_Ku_c17721_26864251	4B	573	72	QPHS.wsu-4B.2	73	Martinez et al., 2018
PHS	wsnp_Ra_c4330_7871129	6D	67	49	cfd37.208	60	Kulwal et al., 2012
PHS	wsnp_RFL_Contig1735_856501	7B	264	NA	wPt-8283	46.5	Kulwal et al., 2012
PHS	wsnp_CAP11_rep_c6622_3044459*	7B	328	46.8	wPt-8283	46.5	Kulwal et al., 2012
Height	wsnp_Ex_c2388_4476302	2B	24.8	NA	Ppd-B1	18	Zhou et al., 2016
Height	wsnp_Ex_c10961_17803258*	2B	8.5	16	Ppd-B1	18	Zhou et al., 2016
Height	wsnp_Ex_c12850_20377830	3A	508	75.2	qRht.3A	75.9	Würschum et al., 2017
Height	wsnp_Ku_c5566_9864771	4B	645	38.6	Rht-B1	47.8	Würschum et al., 2017
Height	wsnp_Ra_c61979_62214892	6A	105	89.3	Rht24	93.7	Würschum et al., 2017
Height	wsnp_Ex_c17692_26437459	6A	113	89.7	Rht24	93.7	Würschum et al., 2017
Height	wsnp_Ra_c16745_25482384	6A	115	89.7	Rht24	93.7	Würschum et al., 2017
Height	wsnp_Ex_c16480_24986490	6A	494	114	Rht24	93.7	Würschum et al., 2017
SftEqv	wsnp_CAP11_c710_458019	1A	8	10.2	wPt-7541	2.5–7.9	Cabrera et al., 2015
LASRC	wsnp_Ku_c4911_8795151	1B	60	23.7	wPt-7094	9.0-27	Cabrera et al., 2015
LASRC	wsnp_Ku_c16938_25916279	1B	109	NA	wPt-1684	35	Cabrera et al., 2015
LASRC	wsnp_Ex_c20975_30093113	1B	118	NA	wPt-1684	35	Cabrera et al., 2015
LASRC	wsnp_BE399980B_Ta_2_1*	1B	143	30	wPt-1684	35	Cabrera et al., 2015
NaSRC	wsnp_JD_c2636_3554874	1B	563	NA	wPt-2526	87.9–88.3	Cabrera et al., 2015
NaSRC	wsnp_Ex_c22439_31632880*	1B	563	77.7	wPt-2526	87.9–88.3	Cabrera et al., 2015
FlProt	wsnp_Ex_rep_c66509_64775661*	3B	236	72.5	wPt-1940	68.6	Cabrera et al., 2015
FlProt	wsnp_BE591466B_Ta_2_1	3B	259	NA	wPt-1940	68.6	Cabrera et al., 2015
NaSRC	wsnp_JD_c5643_6802088	3B	618	95.5	wPt-6785	85.9–87.4	Cabrera et al., 2015
NaSRC	wsnp_JD_c5643_6802211	3B	618	96.7	wPt-6785	85.9–87.4	Cabrera et al., 2015
FlYld	wsnp_Ex_rep_c68165_66935148	6A	13	25.5	wPt-8266	16	Cabrera et al., 2015
FlYld	wsnp_Ex_rep_c68165_66935041	6A	13	25.5	wPt-8266	16	Cabrera et al., 2015
FlYld	wsnp_Ex_c3530_6459643	6A	13	25.5	wPt-8266	16	Cabrera et al., 2015
WatSRC	wsnp_BE497701B_Ta_2_1	6B	595	99.5	wPt-5176	112-120	Cabrera et al., 2015

1: Calculated from Wang et al. (2014b).

#: The significant marker was not available in Wang et al. (2014b).

*: Closest marker in Wang et al. (2014b) to calculate cM distance.

FN negatively correlated with AA by Pearson’s correlation and were at opposite quadrants in PCA. This was expected as higher alpha amylase activity breaks down more starch, reducing hot flour paste thickness and thus FN (Patwa and Penning, 2020). The correlation of TstWt and PHS resistance was observed in both PCA and Pearson’s tests. A near-significant TstWt at the same marker for GWAS models, MLM, MLMM, and GLM as PHS resistance on chromosome 1B at 382 million base pairs (MBPs) was observed (data not shown). Statistical significance has been reported between TstWt and PHS in another population (Ji et al., 2018). Seed color was strongly correlated with PHS measures and PCA indicated a tight positive link between FN and SdClr. Red seed color has been widely reported as significant to PHS resistance and a gene has been located (Lin et al., 2016; Ji et al., 2018; Patwa and Penning, 2020). No QTN for PHS and SdClr on chromosome 3A near *MFT* was found, but the study had few mostly related white seed color varieties. Detection would be difficult due to the low minor allele frequency and a strong population structure. A highly significant QTN for PHS was observed on chromosome 4A in the region of a known PHS resistance gene, *MKK3* (Torada et al., 2016). A total of 36 QTNs at 25 regions related to PHS resistance were observed. The majority of PHS QTN regions were field events, both natural weathering (Nat) and by overhead irrigation (Irr), that were co-located and offer potential new targets to improve PHS including a highly significant QTN on chromosome 1A. None of the PHS regions overlapped with flour quality traits and only one overlapped with HD; thus, improvement of PHS may be obtained without impacting crop or baking performance. Artificial spike wetting (Art) had fewer QTNs and only one overlap on chromosome 4A at 604 MBP with field-treated samples (Irr or Nat). Converting regions to approximate cM, no exact marker matches were found in the literature, but five QTN regions were within 1 to 8 cM. 1A-4, 1B-3, and 4B-3 were compared to a GWAS of 469 club and soft white wheat germplasm from the Pacific Northwest (Martinez et al., 2018). Four QTN regions were observed within 0.3 to 11 cM of a GWAS with 198 elite Eastern US soft white winter wheat lines 1B3, 4A-4, 6D-1, and 7B-1 out of 11 regions (Kulwal et al., 2012). All close matches are listed in **Table 3**. Thus, population, method of association, and/or environment appeared to play a key role in locating PHS resistance, although some consistent regions were found.

High genetic variation in a number of flour quality traits including FlYld, FlProt, multiple SRCs, SftEqv, and measures of cookie quality has been previously suggested for this population, but these soft wheat lines have maintained relatively stable quality standards without large improvements (Souza et al., 2012). This study has revealed potential changes of flour protein over time, but it remains possible to improve many other flour quality traits utilizing this diverse population of historic breeding lines by finding and understanding the genes underlying flour quality traits. Soft wheat flour is used mostly for baked confections such as cookies and cakes. Depending on the end use of soft wheat flour, a higher or a lower FlProt may be desired. For baking powder biscuits and pound cake, a slightly higher FlProt can increase height (Wilderjans et al., 2008; Ma and Baik, 2018). Higher protein can increase the volume and reduce collapse in pound cake but decreases CkDia and cookie spread factor (Wilderjans et al., 2008; Souza et al., 2011). Co-located flour quality QTNs had expected positive and negative values when considering individual markers. Lower FlProt or gluten strength measured by LASRC and LAAdSRC is considered a positive trait for most soft winter wheat applications except crackers (Souza et al., 2012; Deng et al., 2021). Flour quality traits showed strong overlap among themselves by Pearson’s correlation, PCA, and GWAS, indicating high potential genetic overlap affecting these traits. Solvent retention capacity measures for flour quality were tightly grouped by PCA and were strongly positively correlated with co-localizations on chromosomes 1A, 1D, 2A, 5A, and 6B. This is not surprising considering the close associations of SRCs with overall water absorption (WatSRC) in baking performance (Souza et al., 2012). Similarly, SRCs, SftEqv, and FlProt were correlated, positively or negatively with CkDia and CkTpGr. Negative correlations of SRCs (WatSRC, NaSRC, SucSRC, and LASRC) with FlYld and cookie quality (CkDia and CkTpGr) have long been known but the underlying genes are not yet discovered (Guttieri and Souza, 2003). Multiple flour quality co-localizations occurred on chromosomes 1B, 2D, 3A, 5A, 6A, and 6B. Using a completely different marker set and different GWAS models for the population, overlap with eight QTL reported by Cabrera et al. (2015) was observed (QTN 1A-2, 1B-1, 1B-2, 1B-5, 3B-2, 3B-5, 6A-2, and 6B-6) and additional QTNs were found on 17 of 21 chromosomes (**Tables 2**, **3**). These regions deserve closer investigation with additional populations, marker sets, and/or experiments to eventually isolate genes influencing these traits.

The A and B genomes were well covered with an average of 128 markers per chromosome except chromosome 4B with 55 markers where only one QTN was identified. The D genome had fewer markers per chromosome, averaging 24. Reduced sequence diversity may have been a factor. Single-nucleotide polymorphism markers placed on the D genome were about 20% of those on the A and B genomes for the iSelect SNP array due to lack of sequence differences (Cavanagh et al., 2013). While chromosome-level cutoffs were used as in Hill et al. (2022), most of the QTNs on the A and B genomes were significant at the much more restrictive experiment-wise Bonferroni cutoff while most on the D genome were not. Additional markers from single-nucleotide polymorphisms will be needed to populate the D genome and test available diversity, which should improve chromosome-wise significant regions to experiment-wise significance. Increased markers on the D genome would reduce false positives while potentially improving QTN detection with stronger *p*-values by having markers closer to the responsible region. This was observed in the A and B genomes at regions 1B-1, 3A-2, 3A-6, 3A-7, 4A-2, 5A-1, 6A-2, and 6B-2 where a more significant peak for a trait was detected along with a less significant outer marker for that same trait often with SUPER and GLM models forming the tail. However, it is impossible in a population of this size to definitively differentiate a less significant tail from two closely linked genes.

GAPIT3 allowed testing of multiple GWAS models. Nine traits were normally distributed by the Shapiro–Wilk test; however, the majority had near-normal-appearing bell curves by histogram and *W* test statistics > 0.9, indicating that most values for each trait fit in standard normal quartiles, except ArtAA and NatAA with a limited range of values; Age, which is heavily skewed to newer lines; and Awns and SdClr, which were either/or classifications and, thus, binary. The six models tested did not appear to show any abnormal calling of significant locations for SdClr, which, being binary (red or white) with the lowest *W* statistic and fewer white varieties, would have been the most susceptible to false positives. The detection of Age-related QTN across all models with the exception of MLM was also robust regardless of it being skewed more towards more recent breeding lines (less than 60 years since release) as fewer very old breeding lines are available. Single-locus tests such GLM, a naïve model without population correction, and MLM using VanRaden kinship were tested along with more recently released models. The GLM can be particularly prone to false positives as illustrated by the QQ plot for Age but false positives appear less likely with the QQ plot for IrrAA. The MLMM performs multiple locus tests and is an extension of MLM with the most significant marker fitted stepwise as a co-variate (Wang and Zhang, 2021). The FarmCPU model iterates back and forth with two models, an MLM with VanRaden kinship and a GLM without kinship, reported to be faster and provide higher statistical power (Wang and Zhang, 2021). The BLINK model replaces MLM in FarmCPU with Bayesian-information content (Wang and Zhang, 2021). The SUPER model assesses marker effects like GLM, optimizes a kinship model using maximum likelihood, and tests a third time with a kinship derived from markers not in linkage disequilibrium with the tested marker (Wang et al., 2014a; Wang and Zhang, 2021). The GLM as a naïve model lacking population structure correction overestimated QTN as has been reported previously (Larsson et al., 2013). The SUPER model also reported increased numbers of QTN but often near other QTNs for the same traits significant for multiple models. The MLM and MLMM gave very similar results but appeared to underestimate QTN. Similarly, BLINK, FarmCPU, and SUPER often located the same QTN but a different set from MLM/MLMM. Since QTN differences were found based on model selection, requiring agreement of at least two models boosts confidence in reported QTN. A much stronger case for significance can be made where all models agree. However, owing to the computational intensity of testing six models as more markers or individuals are added, selecting those with the widest differences may prove more efficient. In this population, MLMM, FarmCPU, and BLINK were the most obvious choices as they were the least computationally impactful and covered the most QTN differences with less likelihood for overestimation. The BLINK model was chosen over SUPER as it does not assume causal genes are distributed evenly, provides more diversity to the chosen models, and leads to less overestimation in this population (Wang and Zhang, 2021).

## 5 Conclusions

Breeding over time appeared to be a factor in some traits, most notably flour protein content, Hght, and HD. Since PHS-related traits had little overlap with others, several new regions were uncovered to increase resistance in wheat under adverse weather conditions brought about by climate change without impacting agronomic performance or flour quality. Flour quality traits showed much greater overlap both among themselves and with agronomic traits making improvement more challenging. Future research to increase marker density should better resolve QTN and improve detection in areas lacking sufficient coverage utilizing a smaller set of GWAS models.

## Data availability statement

All data used to generate results during this study are available as **Supplementary Information** files to this article.

## Author contributions

NP performed some of the experiments, wrote and edited some text. BP designed and supervised the experiments, analyzed the data, wrote and edited text. All authors contributed to the article and approved the submitted version.

## Supplementary material

File S1 Trait data set used to calculate BLUPs. Includes name, accession number, release year and all calculated values of traits including the means, standard deviations, and number of reps for each soft winter wheat variety used to calculate the BLUPs.

File S2 R Scripts for statistical analyses and GWAS. Includes all R scripts with notes used to generate the Pearson’s correlations, principal components analysis, genome-wide association study, and **Figure 2**.

File S3 Trait data as BLUPs used in GAPIT for GWAS. Twenty-two sets of the trait data as BLUPs for 188 soft winter wheat varieties used to perform the genome-wide association, myY in the GWAS R script.

File S4 Marker data in HAPMAP format used in GAPIT for GWAS. Twenty-two sets of 1978 markers in HAPMAP format for the genotypes used to perform the GWAS, myG in the GWAS R script.

File S5 Histograms, Manhattan and Q-Q Plots for all traits and models of the GWAS. Twenty-two sets of histograms of BLUPs. Twenty Q-Q plots and stacked Manhattan plots with six models each for all traits except Age and IrrAA in **Figures 3**, **4**.

## Glossary

**Table:**

AA	Alpha amylase activity
AACC	American Association of Cereal Chemists
Age	Age of breeding line
Art	Artificial
ArtAA	Artificial alpha amylase activity
ArtFN	Artificial Falling Number
Awns	Awnless or bearded
BLINK	Bayesian-information and linkage-disequilibrium iteratively nested keyway
BLUP	Best Linear Unbiased Predictors
CkDia	Cookie diameter
CkTpGr	Cookie top grade
cM	centiMorgans
*ERA8*	*ENHANCED RESPONSE TO ABA8*
FarmCPU	fixed and random model circulating probability unification
FlProt	Flour protein
FlYld	Flour yield
FN	Falling Number
GLM	general linear model
GWAS	genome-wide association study
HD	heading date
*HvCYP707A1*	*ABA 8’-hydroxylase*
*HvNCED1*	*9-cis-epoxycarotenoid dioxygenase*
Hght	plant height
Irr	Irrigated
IrrAA	Irrigated alpha amylase activity
IrrFN	Irrigated Falling Number
LAAdSRC	Adjusted lactic acid solvent retention capacity
LASRC	Lactic acid solvent retention capacity
MBP	million base pairs
*MFT*	*TaPHS1/Mother of Flowering Time*
*MKK3*	*Map Kinase Kinase 3*
MLM	mixed linear model
MLMM	multiple loci mixed model
NaSRC	Sodium carbonate solvent retention capacity
Nat	Natural weathering
NatAA	Natural alpha amylase activity
NatFN	Natural Falling Number
PHS	pre-harvest sprouting
PC	principal components
PCA	principal components analysis
QQ	quartile–quartile
QTL	quantitative trait loci
QTN	quantitative trait nucleotides
SdClr	seed color
SNP	single-nucleotide polymorphism
SftEqv	Softness Equivalence
SRC	solvent retention capacity
SucSRC	Sucrose solvent retention capacity
SUPER	settlement of MLM under progressively exclusive relationship
TstWt	test weight
*VP1*	*VIVIPAROUS 1*
WatSRC	Water solvent retention capacity.
