# Supplementary material for: Genetics of a diverse soft winter wheat population for pre-harvest sprouting, agronomic, and flour quality traits
Source: Front Plant Sci. 2023 Jun 6;14:1137808. doi: 10.3389/fpls.2023.1137808 (PMC10280069; doi:10.3389/fpls.2023.1137808)
Supplement: File S1 — Trait data set used to calculate BLUPs. Includes name, accession number, release year and all calculated values of traits including the means, standard deviations, and number of reps for each soft winter wheat variety used to calculate the BLUPs. [file DataSheet_1.zip › File S2.DOCX]

**# R scripts for Wheat HD panel Frontiers article**

**# Note > indicates the start of a line of code and + indicates a**

**# continued line of code on a new line (remove + before execution in R)**

**# R script example to calculate BLUP**

**# Load Libraries and data**

> library(metafor)

> datMN_Raw <- read.csv (

+ "FinalFrontierDataSet_ForBLUP_Raw_5_13_21.csv")

**# Example data analysis**

**# Note The dataframe datMN_Raw contained headers corresponding to**

**# a mean (trait_Mean) or mi, standard deviation (trait_StdDev) or**

**# sdi and number of samples (trait_No) or ni for each trait of**

**# each sample, see Supplemental File 1**

> datMN_R_ArtAA <- escalc(measure = "MN", mi=ArtAA_Mean,

+ sdi=ArtAA_StdDev, ni=ArtAA_No, data=datMN_Raw)

**# Note the above generates values for yi and vi used below**

> res_ArtAA_R <- rma(yi, vi, data=datMN_R_ArtAA, control =

+ list(stepadj=0.5, maxiter=1000))

**# Note the BLUP value is generated and stored as pred in the**

**# res_trait frame and pulled into an Excel readable file below**

> blups_ArtAA_R <- blup(res_ArtAA_R)$pred

> write.csv (blups_ArtAA_R,

+ "PHS_ArtAA_Raw_blups_frac_MN_5_14_21.csv")

**# R Script for GAPIT**

**# Note Only one example script shown with info for dataset**

**# The Files from GAPIT were downloaded from**

**# http://www.zzlab.net/GAPIT/ on 5/27/2021**

**#**

**# Load GAPIT files and Datasets**

> setwd("D:\\TASSEL\\PHS_FrontierData\\GAPIT_RunPHS")

> source("GAPIT.library.R.txt")

> source("gapit_functions.txt")

> myY <- read.table(

+ "FinalFrontierDataSet_BLUPs_forGAPIT.txt",

+ head = TRUE)

> myG <- read.delim("HD_HAPMAP_V1_0_GAPIT_Format.txt",

+ head = FALSE, sep = "\t")

**# Example of GAPIT test for one trait**

> myGAPIT_MLM <- GAPIT(

+ Y=myY[,c(1,2)], #first column variety, second ArtAA_BLUPraw

+ G=myG,

+ PCA.total=3,

+ model=c("GLM", "MLM", "MMLM", "FarmCPU", "Blink", "SUPER"),

+ Multiple_analysis=TRUE)

**# Note the second number in myY was incremented for the next**

**# trait for example c(1,3) for UntAA trait in the next GWAS**

**# until all traits in the trait file were run**

**# Unique header names pulled from the trait data file were added**

**# to the result files after the model name such as:**

**# GLM.UntAA_BLUP.GWAS.Results.csv by GAPIT automatically**

**# R script of PCA on BLUP**

**# Load Libraries and read in file of BLUP data**

> library(factoextra)

> library(pcaMethods)

> PHSdata <- read.csv(

+ "FinalFrontierDataSet_Raw_Physical_BLUPS_6_7_21.csv", header =

+ TRUE)

**# Show amount of missing data**

> tPHSdata <- PHSdata

> sum(is.na(tPHSdata))

[1] 0

**# Impute any missing data**

> pcPHS <- pca(tPHSdata, nPcs=3, method = "ppca")

> imputedPHSdata <- completeObs(pcPHS)

> res.PHS <- prcomp(imputedPHSdata, scale = TRUE)

**# Calculate Eigan values and graph them internally**

> fviz_eig(res.PHS)

**# Show the Cos2 individuals graph for PCs 1 & 2**

> fviz_pca_ind(res.PHS, col.ind = "cos2", gradient.cols = c(

+ "#00AFBB", "#E7B800", "#FC4E07"), repel = TRUE)

**# Show the vectored contributions Graph of traits for PCs 1 & 2**

> fviz_pca_var(res.PHS, col.var = "contrib", gradient.cols = c(

+ "#00AFBB", "#E7B800", "#FC4E07"), repel = TRUE)

**# Show the biplot Graph with vectors of trait contributions and**

**# individuals overlayed for PCs 1 & 2**

> fviz_pca_biplot(res.PHS, repel = TRUE, col.var = "#2E9FDF",

+ col.ind = "#696969")

**# Pull all PCA information into Excel format to perform own**

**# graphs**

**# Get Eigan values and put in CSV file**

> eig.val <- get_eigenvalue(res.PHS)

> write.csv(eig.val, "PCA_PHS_RAW_BLUPs_eigval_7_8_21.csv")

**# Get results for variables and put in CSV files**

> res.var <- get_pca_var(res.PHS)

> write.csv(res.var$coord,

+ "PCA_PHS_RAW_BLUPs_VarCoord_7_8_21.csv")

> write.csv(res.var$contrib,

+ "PCA_PHS_RAW_BLUPs_VarContrib_7_8_21.csv")

> write.csv(res.var$cos2, "PCA_PHS_RAW_BLUPs_VarCos2_7_8_21.csv")

**# Get results for individuals and put in CSV files**

> res.ind <- get_pca_ind(res.PHS)

> write.csv(res.ind$coord,

+ "PCA_PHS_RAW_BLUPs_IndCoord_7_8_21.csv")

> write.csv(res.ind$contrib,

+ "PCA_PHS_RAW_BLUPs_IndContrib_7_8_21.csv")

> write.csv(res.ind$cos2, "PCA_PHS_RAW_BLUPs_IndCos2_7_8_21.csv")

**# Calculate PCs for variables**

> var_coord_func <- function(loadings, comp.sdev){

+ loadings*comp.sdev}

**# Compute Variables Coordinates and put in CSV file**

> loadings <- res.PHS$rotation

> sdev <- res.PHS$sdev

> var.coord <- t(apply(loadings, 1, var_coord_func, sdev))

> write.csv(var.coord,

+ "PCA_PHS_RAW_BLUPs_PCA_Var_Coord2_7_8_21.csv")

**# Compute Variables Cos2 and put in CSV file**

> var.cos2 <- var.coord^2

> write.csv(var.cos2,

+ "PCA_PHS_RAW_BLUPs_PCA_Var_Cos2_7_8_26_21.csv")

**# Compute Variables Contributions and put in CSV file**

> comp.cos2 <- apply(var.cos2, 2, sum)

> contrib <- function(var.cos2, comp.cos2)

+ {var.cos2*100/comp.cos2}

> var.contrib <- t(apply(var.cos2,1, contrib, comp.cos2))

> write.csv(var.contrib,

+ "PCA_PHS_RAW_BLUPs_PCA_Var_Contirb_2_7_8_21.csv")

**# Calculate PCs for Individuals**

**# Compute Individuals Coordinates and put in CSV file**

> ind.coord <- res.PHS$x

> write.csv(ind.coord,

+ "PCA_PHS_RAW_BLUPs_PCA_Ind_Coord_2_7_8_21.csv")

**# Compute Individuals Cos2 and put in CSV file**

> center <- res.PHS$center

> scale<- res.PHS$scale

> getdistance <- function(ind_row, center, scale){

+ return(sum(((ind_row-center)/scale)^2))

+ }

>

> d2 <- apply(imputedPHSdata,1,getdistance, center, scale)

> cos2 <- function(ind.coord, d2){return(ind.coord^2/d2)}

> ind.cos2 <- apply(ind.coord, 2, cos2, d2)

> write.csv(ind.coord,

+ "PCA_PHS_RAW_BLUPs_PCA_Ind_Cos2_2_7_8_21.csv")

**# Compute Individuals Contributions and put in CSV file**

> contrib <- function(ind.coord, comp.sdev, n.ind){

+ 100*(1/n.ind)*ind.coord^2/comp.sdev^2

+ }

>

> ind.contrib <- t(apply(ind.coord, 1, contrib,

+ res.PHS$sdev, nrow(ind.coord)))

> write.csv(ind.contrib,

+ "PCA_PHS_RAW_BLUPs_PCA_Ind_Contrib_2_7_8_21.csv")

**# R script example to calculate Pearson’s Correlations**

> # Correlation Coefficients in R

> setwd("D:\\TASSEL\\PHS_FrontierData")

> PHSdata <- read.csv("FinalFrontierDataSet_Raw_Physical_BLUPS_6_7_21.csv", header = TRUE)

> install.packages("Hmisc")

> library(Hmisc)

> PHScorr <- PHSdata[, c(2,3,4,5,6,7,8,9,10,11,12,13,14,15,16,17,18,19,20,21,22,23,24,25)]

> PearCorr <- rcorr(as.matrix(PHScorr))

> write.csv (PearCorr$r, "RAW_BLUPs_PearCorr_R_8_18_21.csv")

> write.csv (PearCorr$P, "RAW_BLUPs_PearCorr_pvalue_R_8_18_21.csv")

**# R script for the Chromosome Plot Figure 2**

> setwd("~/FY2022/WorkTest/FinalPaperFiles")

> library(chromPlot)

> Ta_gap <- read.table("chromPlot_ChromosomeFile.txt", sep="\t", header=TRUE, stringsAsFactors=FALSE)

> head(Ta_gap)

Chrom Start End

1 1A 1 594102056

2 1B 1 689851870

3 1D 1 495453186

4 2A 1 780798557

5 2B 1 801256715

6 2D 1 651852609

> refGeneTa <- read.table("chromPlot_Bands_1935_LongQTLgroupedCloseMarkers_Only05sig.txt", sep="\t", header=TRUE)

> head(refGeneTa)

Chrom Start End Name Colors

1 1A 2886274 3886275 Age1A1 black

2 1A 7157488 8157489 SftEqv1A1 springgreen3

3 1A 300630580 301630581 Age1A2 black

4 1A 442113597 443113598 IrrAA1A1 red

5 1A 442113599 443113600 NatAA1A1 orange

6 1A 477048517 492123959 FlProt1A1 green3

> qtlGeneTa2 <- read.table("chromPlot_QTLgroupedCloseMarkers_1935_only05sig_11_22_22.txt", sep="\t", header=TRUE)

> head(qtlGeneTa2)

Chrom Start End Name Group Group2

1 1A 2886274 3886275 Age1A1 Age physical

2 1A 7157488 8157489 SftEqv1A1 SftEqv quality

3 1A 300630580 301630581 Age1A2 Age physical

4 1A 442113597 443113598 IrrAA1A1 IrrAA PHS

5 1A 442113599 443113600 NatAA1A1 NatAA PHS

6 1A 477048517 492123959 FlProt1A1 FlProt quality

> chromPlot(gaps= Ta_gap, bands=refGeneTa, segment=qtlGeneTa2, noHist=FALSE, stack = TRUE, figCols = 11, colSegments = c("black","yellow","gold3","grey56", "darkorchid", "darkmagenta","green3","green4","brown4","aquamarine3","red","red4","darkblue","dodgerblue","cyan2","orange","orangered","chocolate","springgreen3","darkcyan","grey28","blue"), legChrom = "5B")
