# Supplementary material for: Genetics of a diverse soft winter wheat population for pre-harvest sprouting, agronomic, and flour quality traits
Source: Front Plant Sci. 2023 Jun 6;14:1137808. doi: 10.3389/fpls.2023.1137808 (PMC10280069; doi:10.3389/fpls.2023.1137808)
Supplement: File S1 — Trait data set used to calculate BLUPs. Includes name, accession number, release year and all calculated values of traits including the means, standard deviations, and number of reps for each soft winter wheat variety used to calculate the BLUPs. [file DataSheet_1.zip › File S5.PDF]

Supplementary File S5. Histograms, Manhattan and QQ plots for all traits.

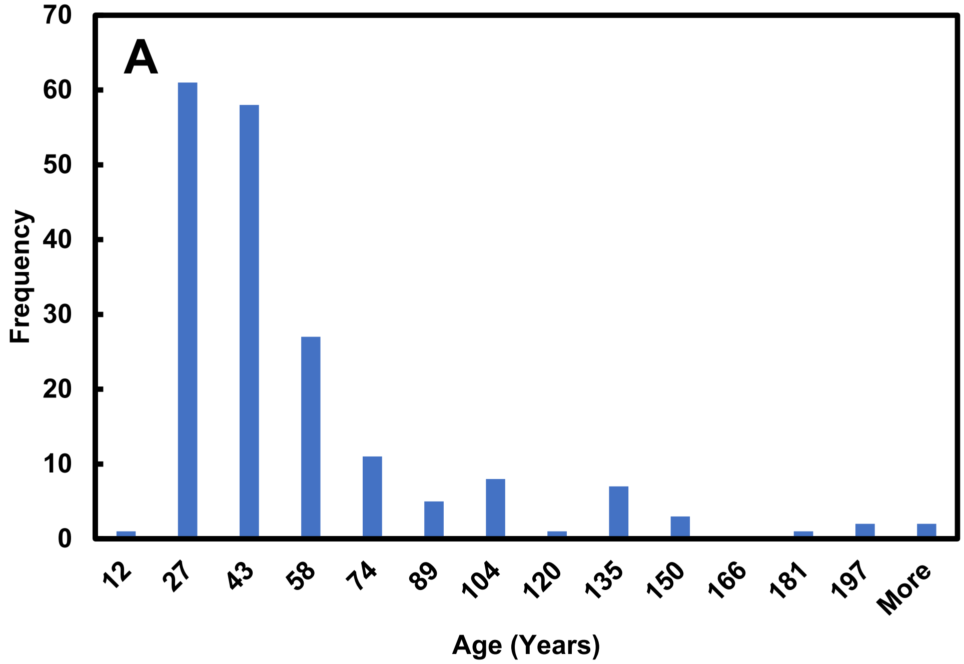

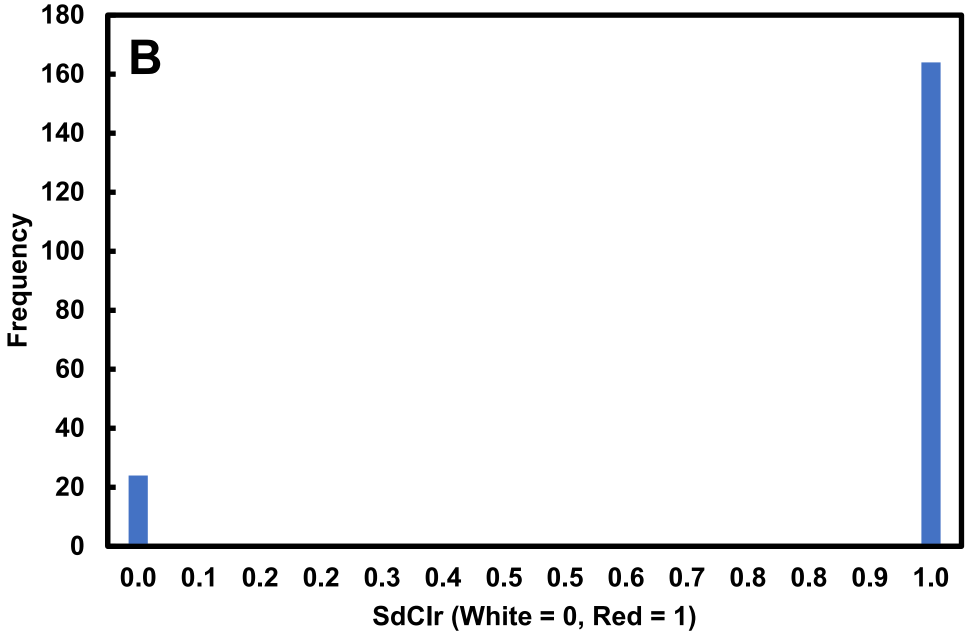

Fig. 1. A. Histogram of Age of breeding line (Age). Calculated as year of release subtracted from 2020. Non-normal as majority of varieties released in the last sixty years cause skew to younger varieties. Shapiro-Wilk W statistic = 0.74 **B.** Histogram of Seed Color (SdClr). White are 0 and red are 1. Majority of population is red, binary distribution. Shapiro-Wilk W statistic = 0.39

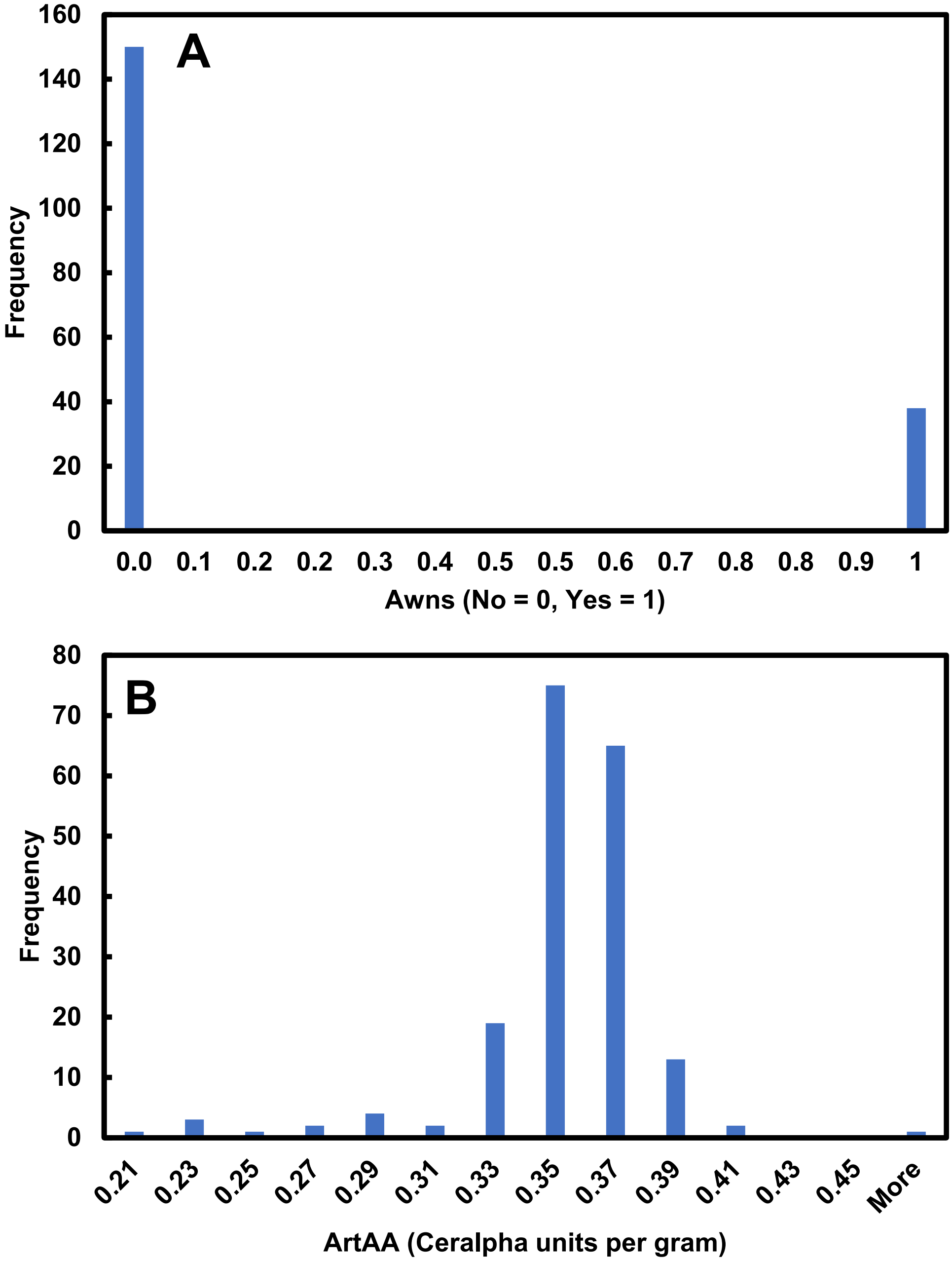

Fig. 2. A. Histogram of Presence of Awns (Awns). No awns are 0 and with awns are 1. Majority of population is not awned, binary distribution. Shapiro-Wilk W statistic = 0.49. **B.** Histogram of Artificial Alpha Amylase Activity (ArtAA). Measurement of PHS. Activity measured in Ceralpha units per gram as per manufacturer's protocol. Majority of population has moderate activity. Non-normal due to limited difference in values. Shapiro-Wilk W statistic = 0.80.

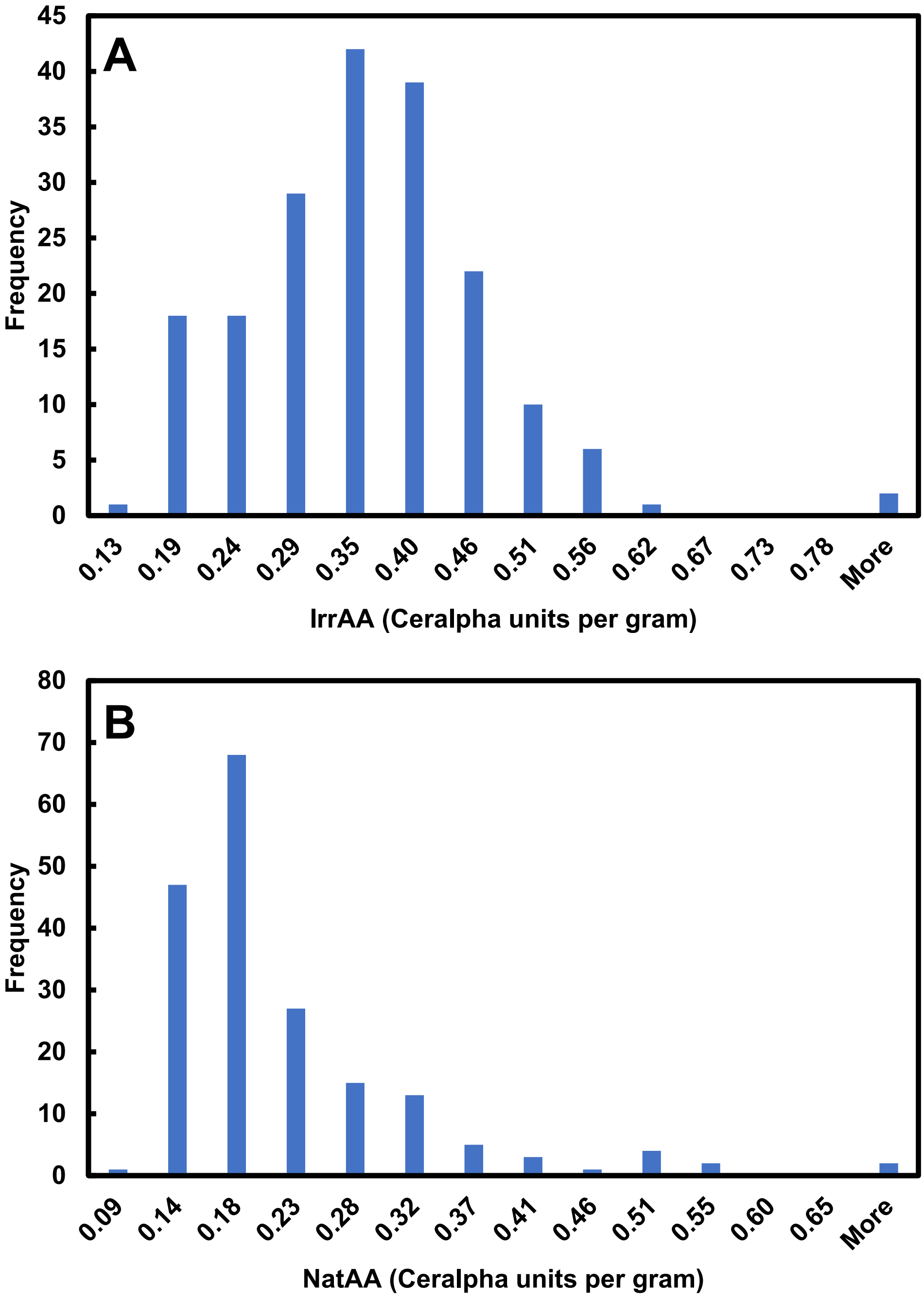

Fig. 3. A. Histogram of Irrigated Alpha Amylase Activity (IrrAA). Measurement of PHS. Activity measured in Ceralpha units per gram as per manufacturer's protocol. Fairly normal distribution, slightly skewed to lower end of activity. Shapiro-Wilk W statistic = 0.95. **B.** Histogram of Natural Weathering Alpha Amylase Activity (NatAA). Measurement of PHS. Activity measured in Ceralpha units per gram as per manufacturer's protocol. Skewed to lower end of activity. Non-normal. Shapiro-Wilk W statistic = 0.77.

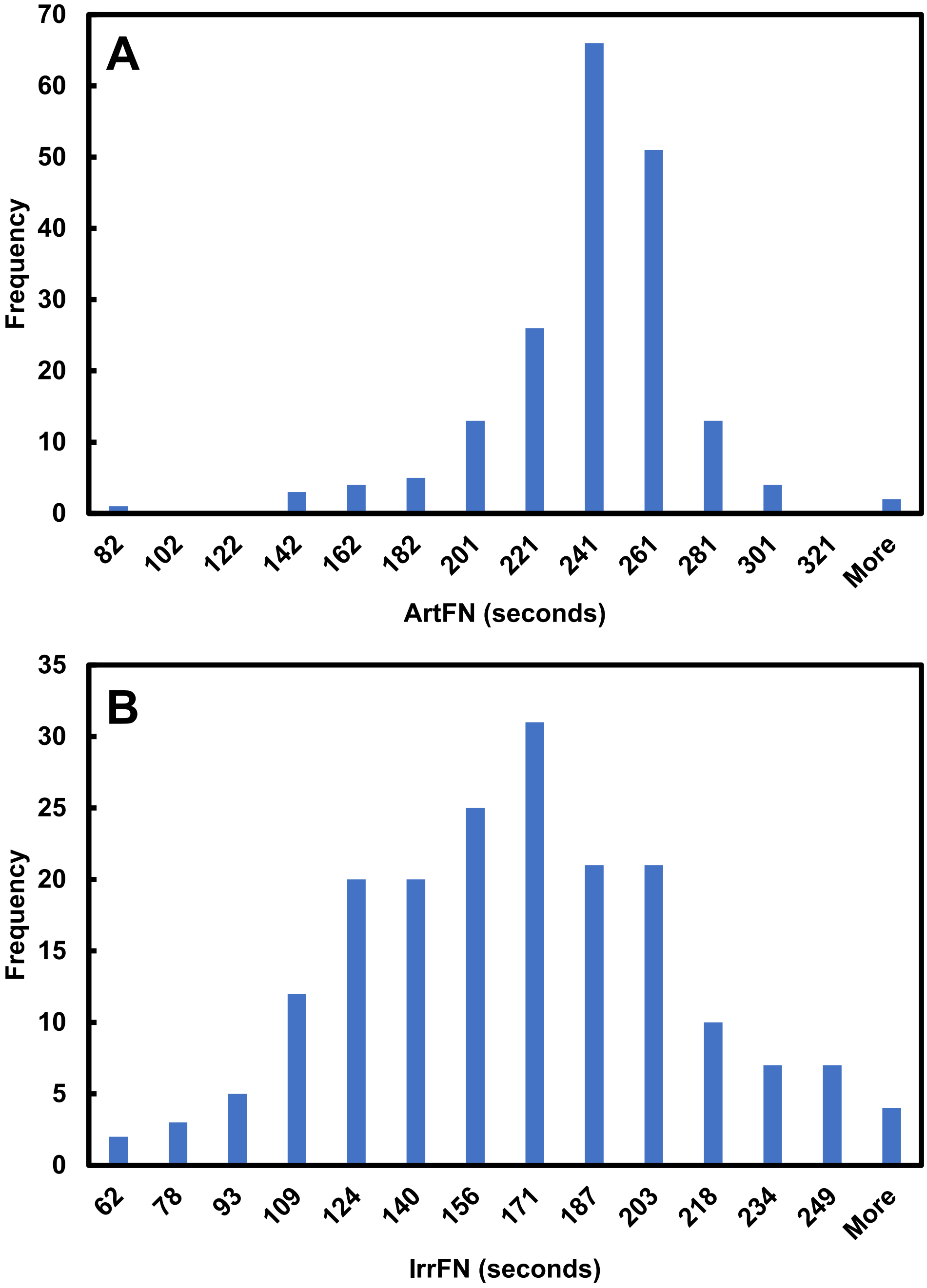

Fig. 4. A. Histogram of Artificial Falling Number (ArtFN). Measurement of PHS. Measured as seconds for plunger to fall through hot paste as per manufacturer's protocol. Majority of population moderate PHS. Near-normal but compressed values, Shapiro-Wilk W statistic = 0.91. **B.** Histogram of Irrigated Falling Number (IrrFN). Measurement of PHS. Measured as seconds for plunger to fall through hot paste as per manufacturer's protocol. Normal distribution, Shapiro-Wilk W statistic = 0.99.

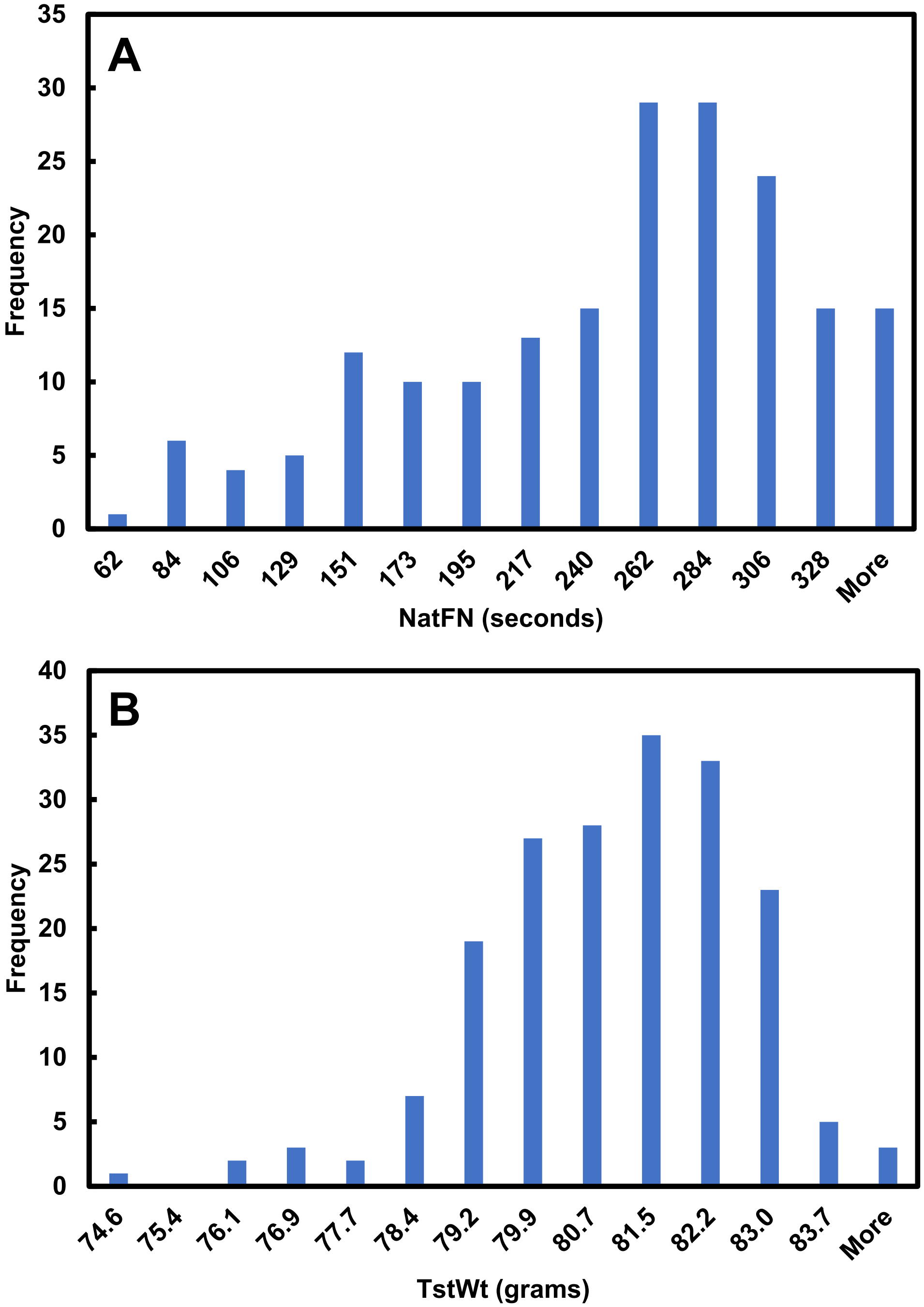

Fig. 5. A. Histogram of Natural Weathering Falling Number (NatFN). Measurement of PHS. Measured as seconds for plunger to fall through hot paste as per manufacturer's protocol. Slightly skewed to high values, less PHS. Shapiro-Wilk W statistic = 0.95. **B.** Histogram of Test Weight (TstWt). Measurement of flour quality. Measured as grams per thousand kernels. Fairly normal skewed towards acceptable weight values. Shapiro-Wilk W statistic = 0.97.

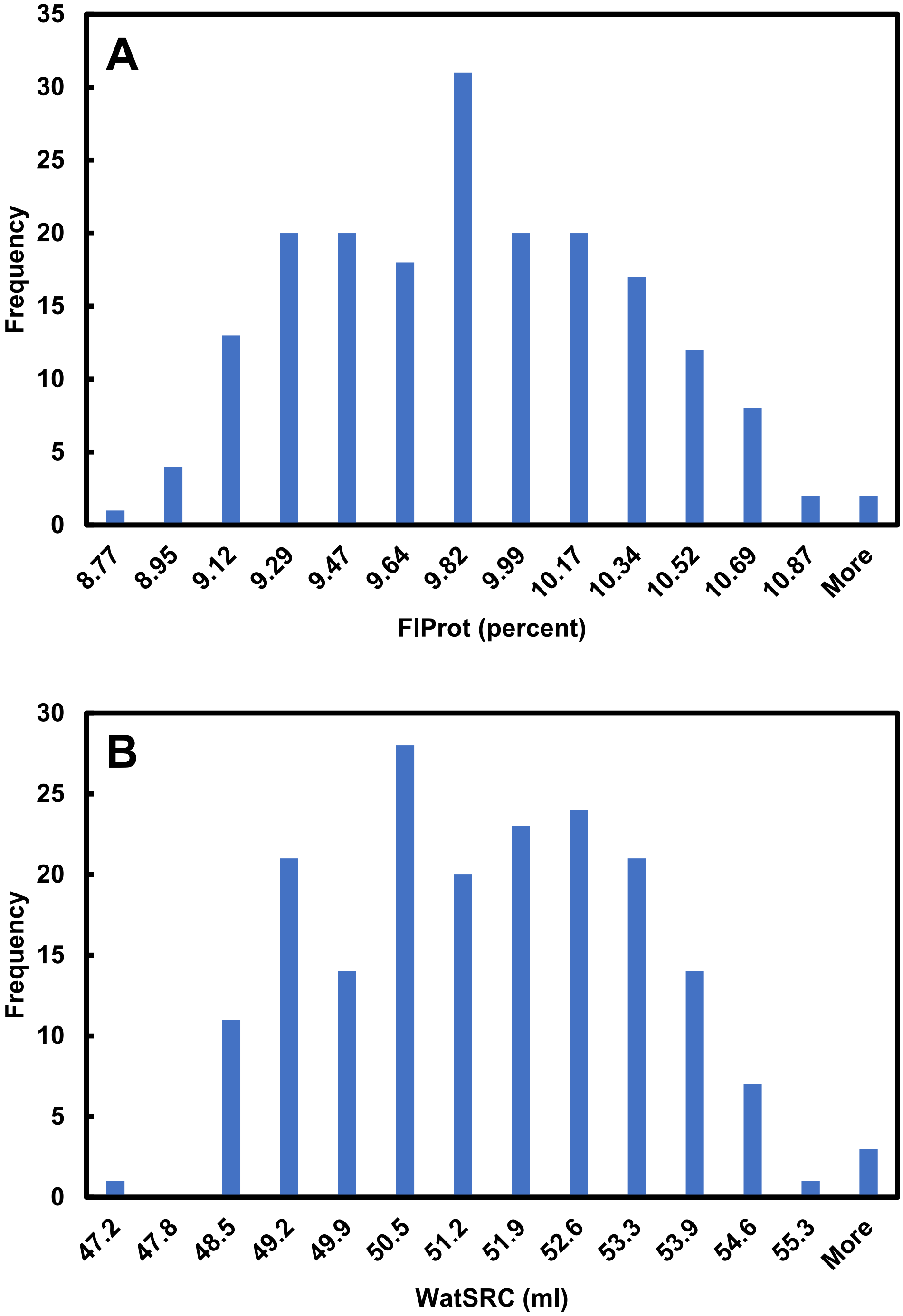

Fig. 6. A. Histogram of Flour Protein (FlProt). Measurement of flour quality. Measured as percent of kernels. Fairly normal around slightly high for good quality. Normal distribution with Shapiro-Wilk W test statistic = 0.99. **B.** Histogram of Water Solvent Retention Capacity (WatSRC). Measurement of flour quality, total absorption capacity. Measured as ml of solvent displaced. Normal distribution with Shapiro-Wilk W test statistic = 0.99.

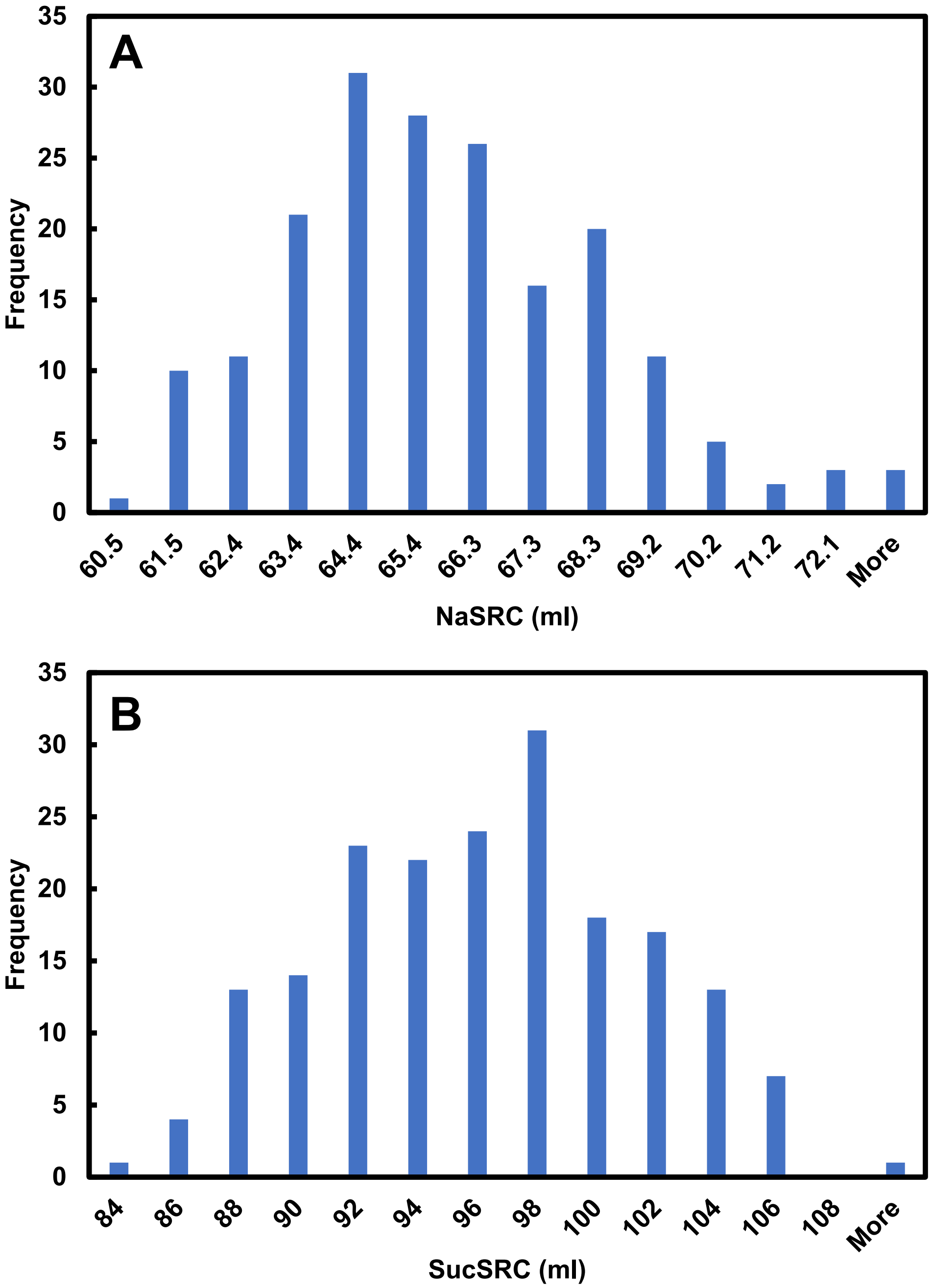

Fig. 7. A. Histogram of Sodium Carbonate Solvent Retention Capacity (NaSRC). Measurement of flour quality, starch damage from milling. Measured as ml of solvent displaced. Fairly normal distribution slightly skewed to higher quality. Shapiro-Wilk W statistic = 0.98. **B.** Histogram of Sucrose Solvent Retention Capacity (SucSRC). Measurement of flour quality, pentosan (arabinoxylan) content. Measured as ml of solvent displaced. Normal distribution with Shapiro-Wilk W statistic = 0.99.

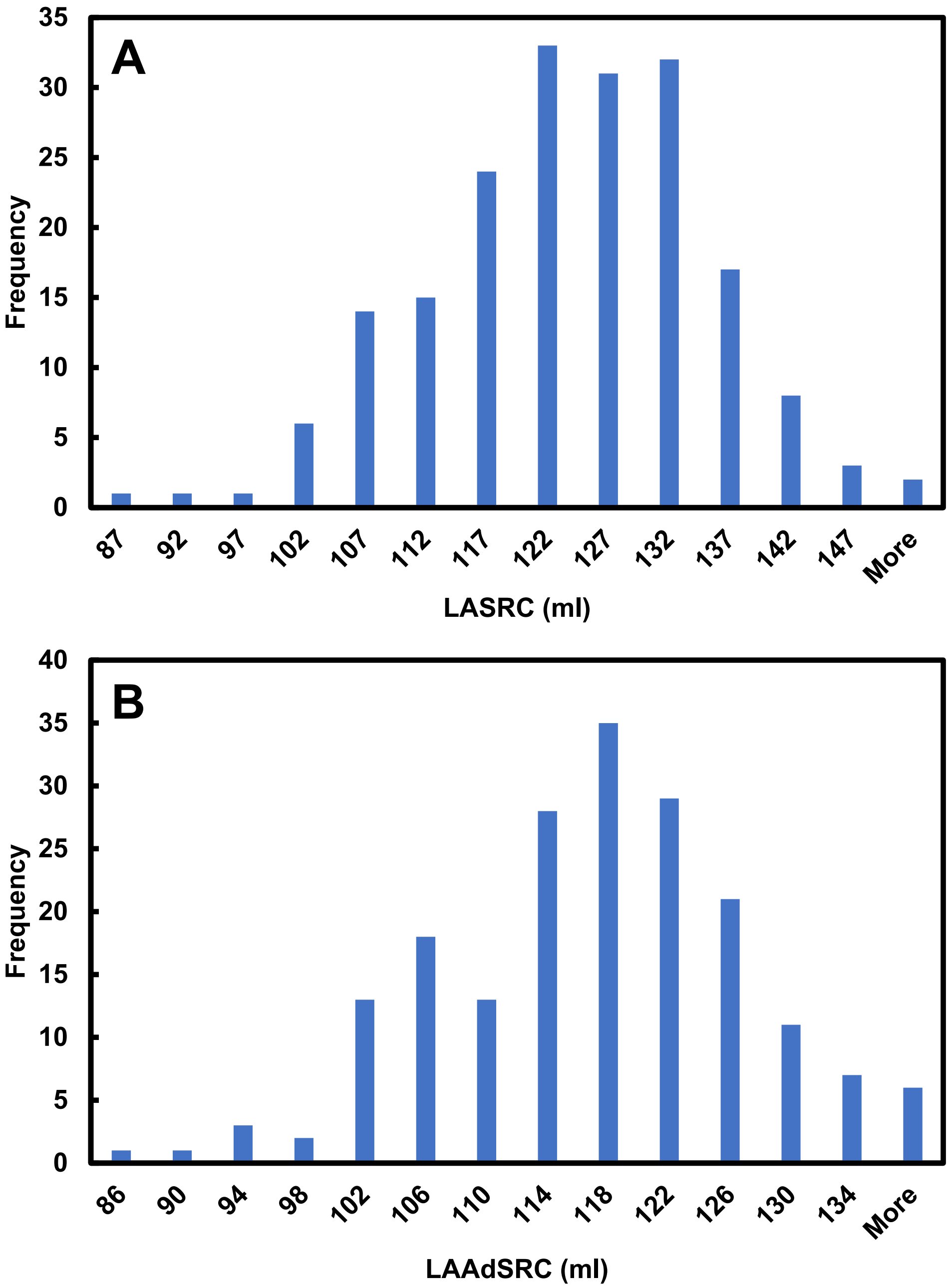

Fig. 8. A. Histogram of Lactic Acid Solvent Retention Capacity (LASRC). Measurement of flour quality, protein strength. Measured as ml of solvent displaced. Normal distribution with Shapiro-Wilk W statistic = 0.99. **B.** Histogram of Adjusted Lactic Acid Solvent Retention Capacity (LASRC). Measurement of flour quality, protein strength adjusted for protein content. Measured as ml of solvent displaced. Normal distribution with Shapiro-Wilk W statistic = 0.99.

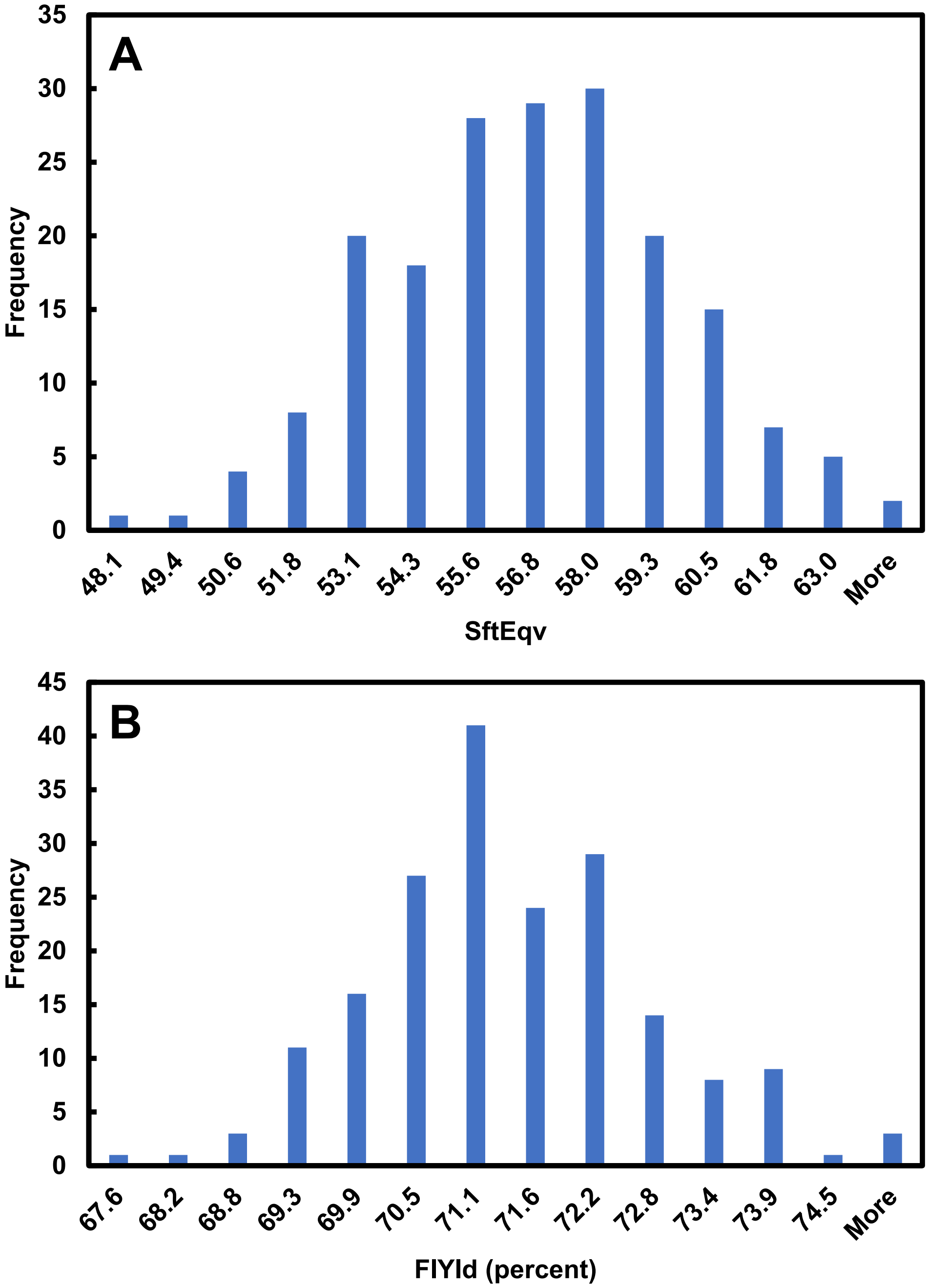

Fig. 9. A. Histogram of Softness Equivalence (SftEqv). Measurement of flour quality, how soft the kernel is, how easy to mill. No Units. Normal distribution with Shapiro-Wilk W statistic = 1.0, slightly high for soft wheat. **B.** Histogram of Flour Yield (FlYld). Measurement of flour quality, how much flour from milling. Percent of kernel. Normal distribution with Shapiro-Wilk W statistic = 0.99.

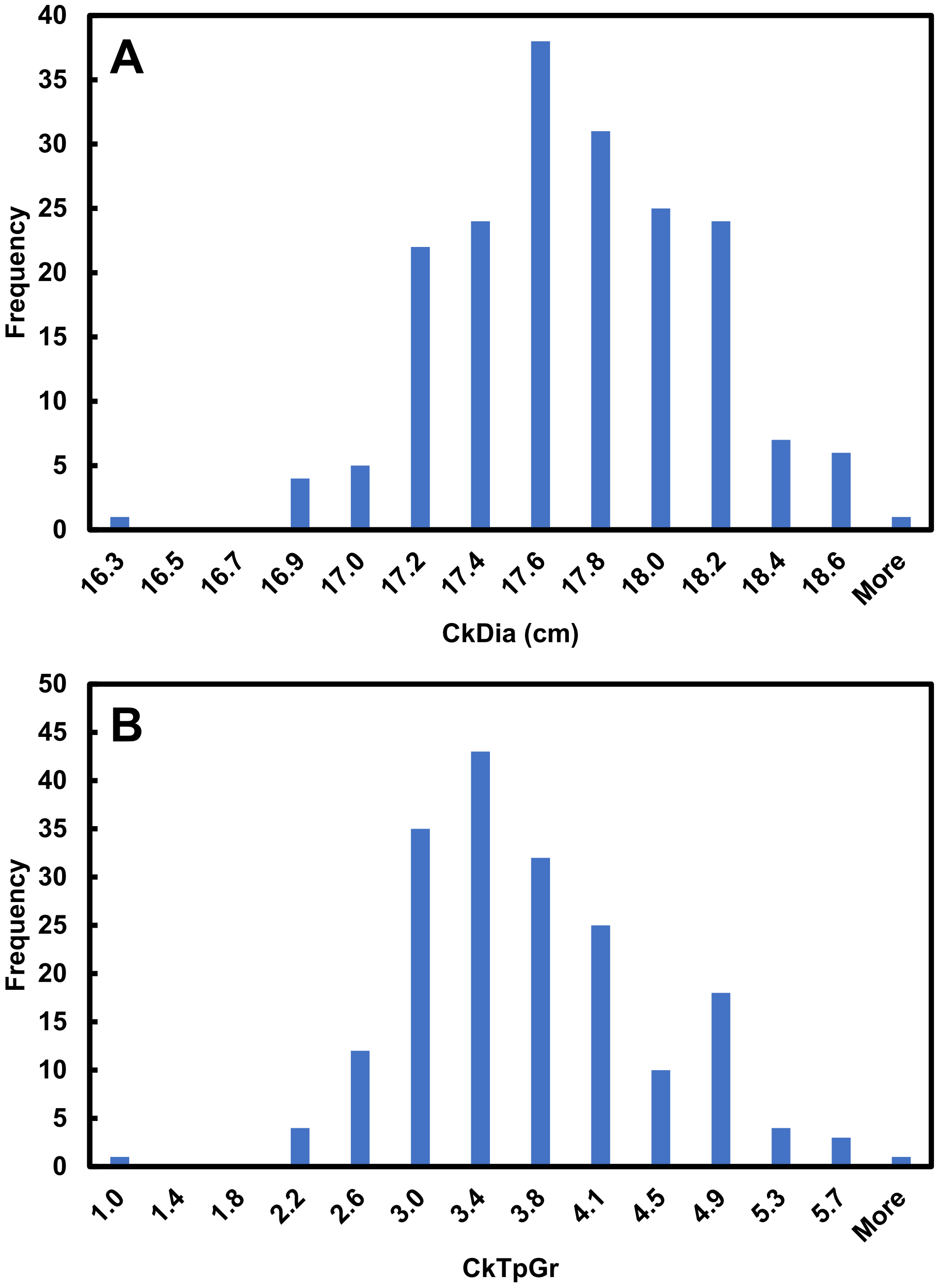

Fig. 10. A. Histogram of Cookie Diameter (CkDia). Measurement of flour quality, how much the cookie spreads when baked in cm. Normal distribution with Shapiro-Wilk W statistic = 1.0, about average for soft winter wheat. **B.** Histogram of Cookie Top Grade (CkTpGr). Measurement of flour quality, how crinkled the cookie looks when baked. No Units, subjective test. Fairly normal distribution slightly low for soft winter wheat. Shapiro-Wilk W statistic = 0.98.

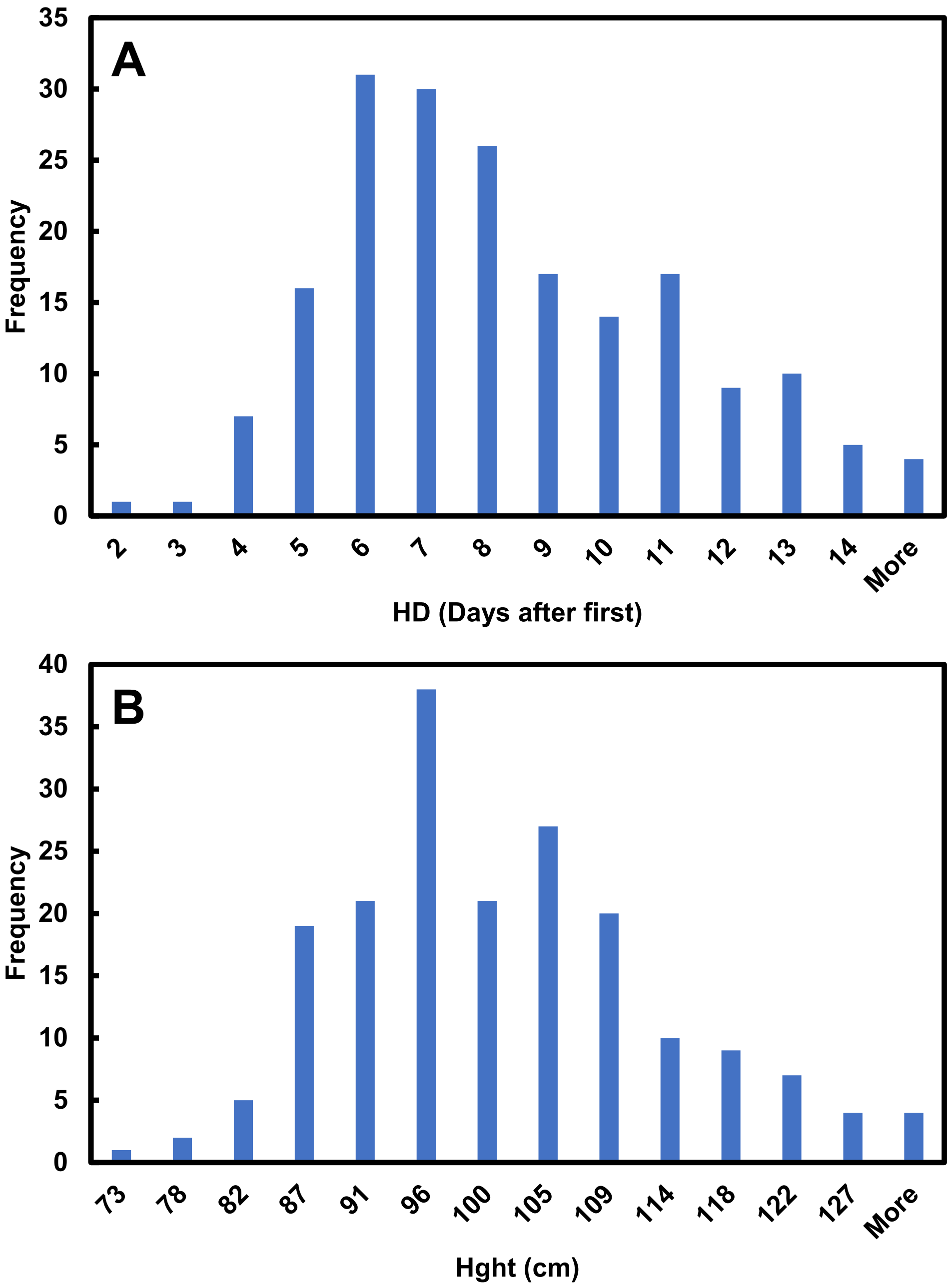

Fig. 11. A. Histogram of Heading Days after first variety heads (HD). Measurement of maturity time. How long it takes for the spike to fully emerge from the boot jack in days. Fairly normal distribution slightly skewed to later maturity. Shapiro-Wilk W statistic = 0.97. **B.** Histogram of Plant Height (Hght). Measurement of plant height in cm. Fairly normal distribution around average for soft winter wheat. Shapiro-Wilk W statistic = 0.97.

A

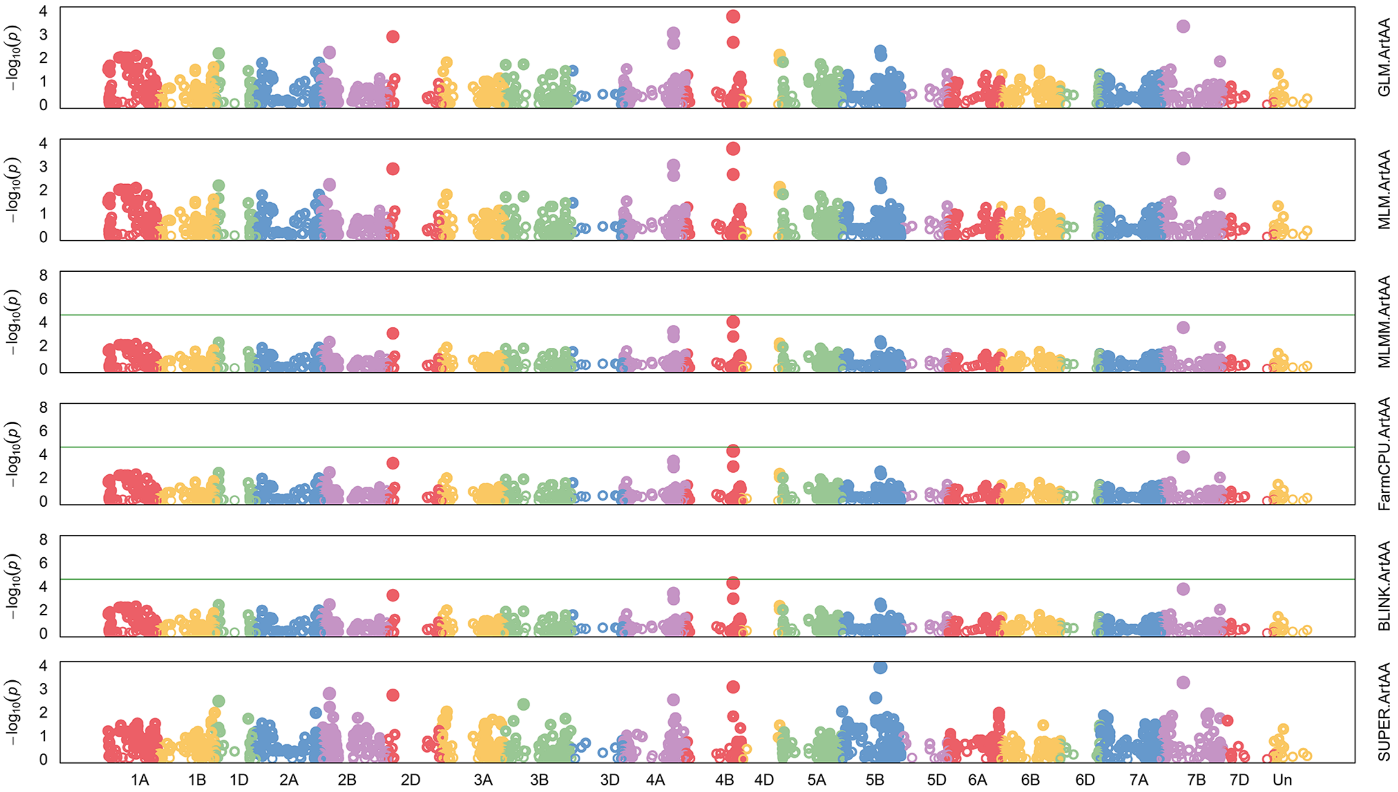

B

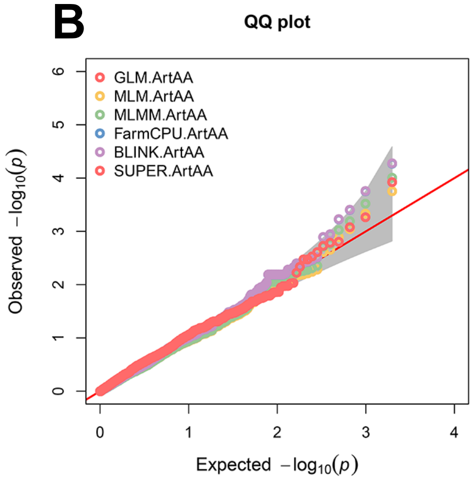

Fig. 12. Genome-wide association for Artificial Alpha Amylase Activity (ArtAA). **A.** Manhattan plots for all six models, GLM, MLM, MLMM, FarmCPU, BLINK, and SUPER (Shown Right). Negative log 10 of p-value for each marker on a chromosome indicated by colored dots. Red horizontal line is the default, more stringent experiment-wise Bonferroni significance threshold in GAPIT3 of alpha = 0.01. Dashed grey vertical lines indicate two models significant for the same marker and solid grey vertical lines, three or more. **B.** QQ Plots for the genome-wide association. Colored circles represent the six different models tested for the trait. Red diagonal line indicates where observed and expected results would match. Grey shaded region is confidence interval and colored circles significantly above the line represent deviations that may be significantly associated with phenotype.

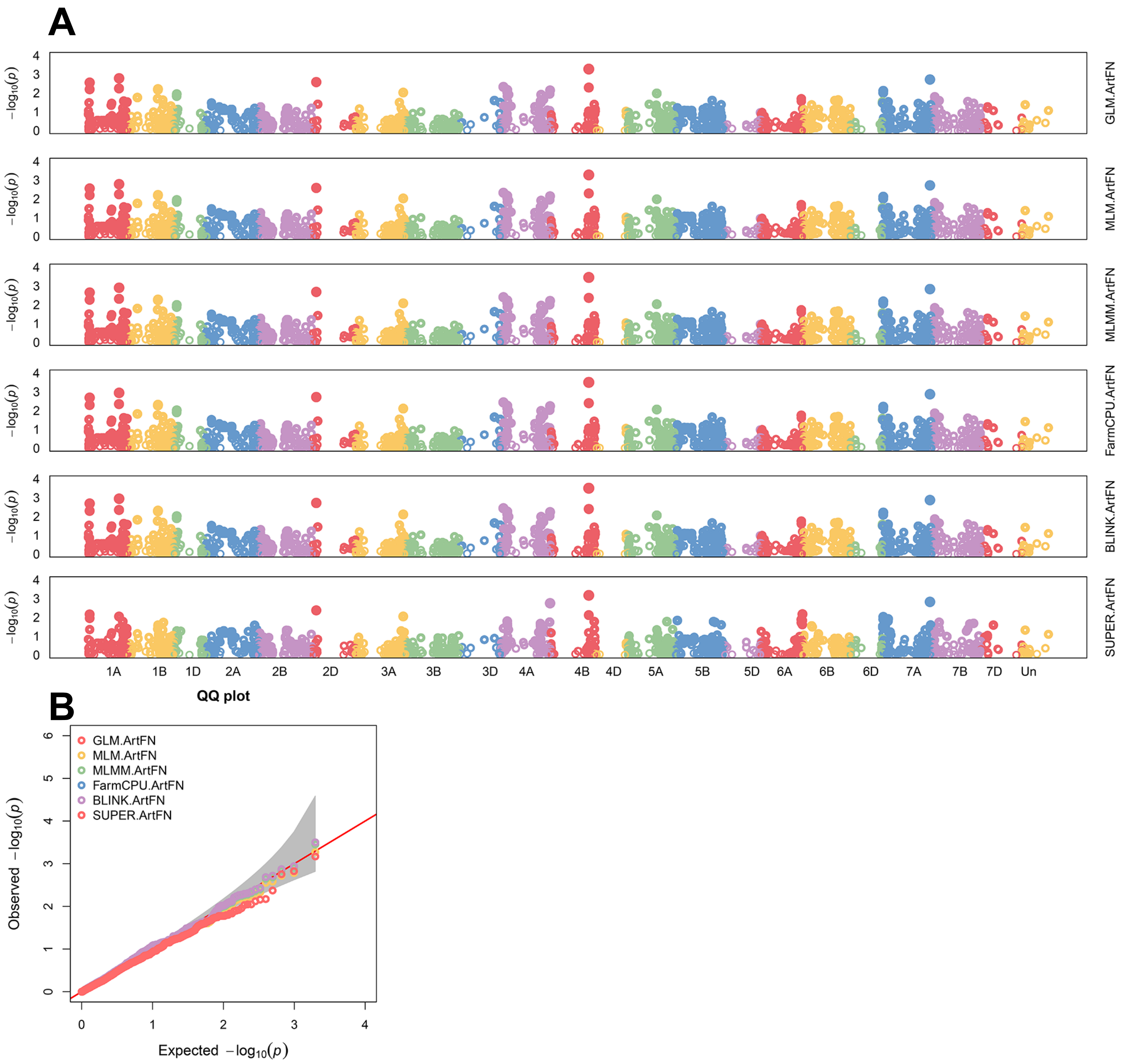

Fig. 13. Genome-wide association for Artificial Falling Number (ArtFN). **A.** Manhattan plots for all six models, GLM, MLM, MLMM, FarmCPU, BLINK, and SUPER (Shown Right). Negative log 10 of p-value for each marker on a chromosome indicated by colored dots. Red horizontal line is the default, more stringent experiment-wise Bonferroni significance threshold in GAPIT3 of alpha = 0.01. Dashed grey vertical lines indicate two models significant for the same marker and solid grey vertical lines, three or more. **B.** QQ Plots for the genome-wide association. Colored circles represent the six different models tested for the trait. Red diagonal line indicates where observed and expected results would match. Grey shaded region is confidence interval and colored circles significantly above the line represent deviations that may be significantly associated with phenotype.

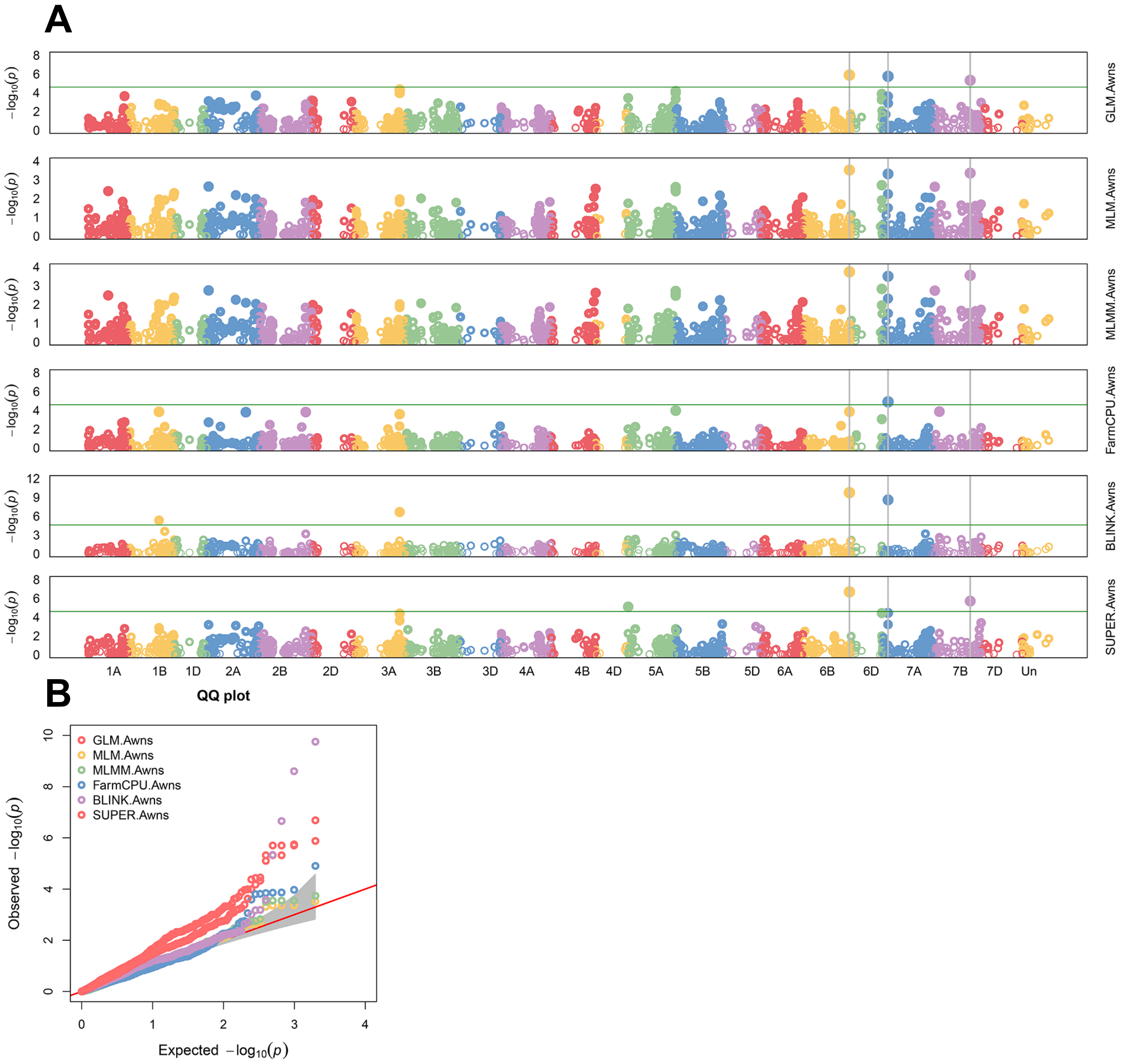

Fig. 14. Genome-wide association for Presence/Absence of Awns. **A.** Manhattan plots for all six models, GLM, MLM, MLMM, FarmCPU, BLINK, and SUPER (Shown Right). Negative log 10 of p-value for each marker on a chromosome indicated by colored dots. Red horizontal line is the default, more stringent experiment-wise Bonferroni significance threshold in GAPIT3 of alpha = 0.01. Dashed grey vertical lines indicate two models significant for the same marker and solid grey vertical lines, three or more. **B.** QQ Plots for the genome-wide association. Colored circles represent the six different models tested for the trait. Red diagonal line indicates where observed and expected results would match. Grey shaded region is confidence interval and colored circles significantly above the line represent deviations that may be significantly associated with phenotype.

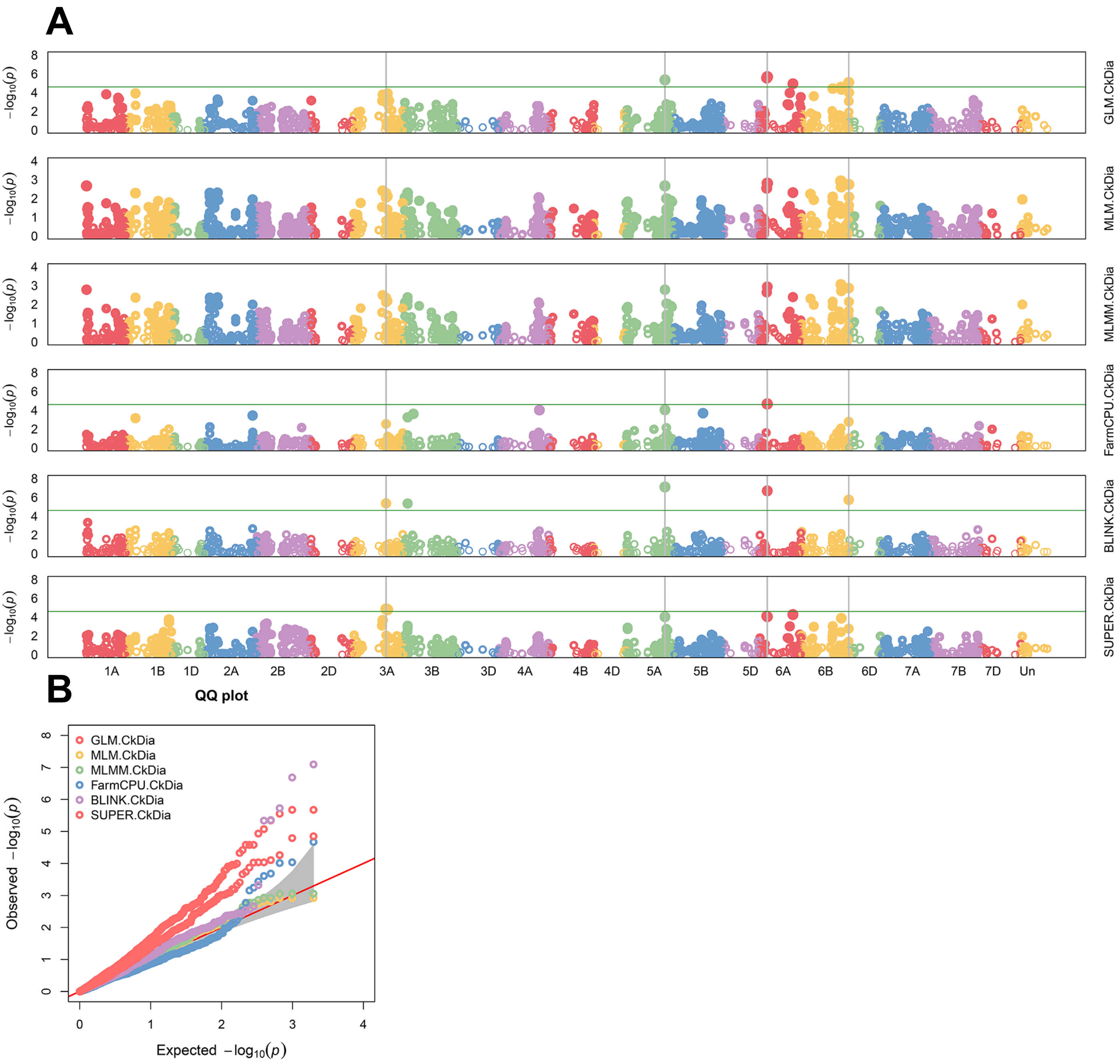

Fig. 15. Genome-wide association for Cookie Diameter (CkDia). **A.** Manhattan plots for all six models, GLM, MLM, MLMM, FarmCPU, BLINK, and SUPER (Shown Right). Negative log 10 of p-value for each marker on a chromosome indicated by colored dots. Red horizontal line is the default, more stringent experiment-wise Bonferroni significance threshold in GAPIT3 of alpha = 0.01. Dashed grey vertical lines indicate two models significant for the same marker and solid grey vertical lines, three or more. **B.** QQ Plots for the genome-wide association. Colored circles represent the six different models tested for the trait. Red diagonal line indicates where observed and expected results would match. Grey shaded region is confidence interval and colored circles significantly above the line represent deviations that may be significantly associated with phenotype.

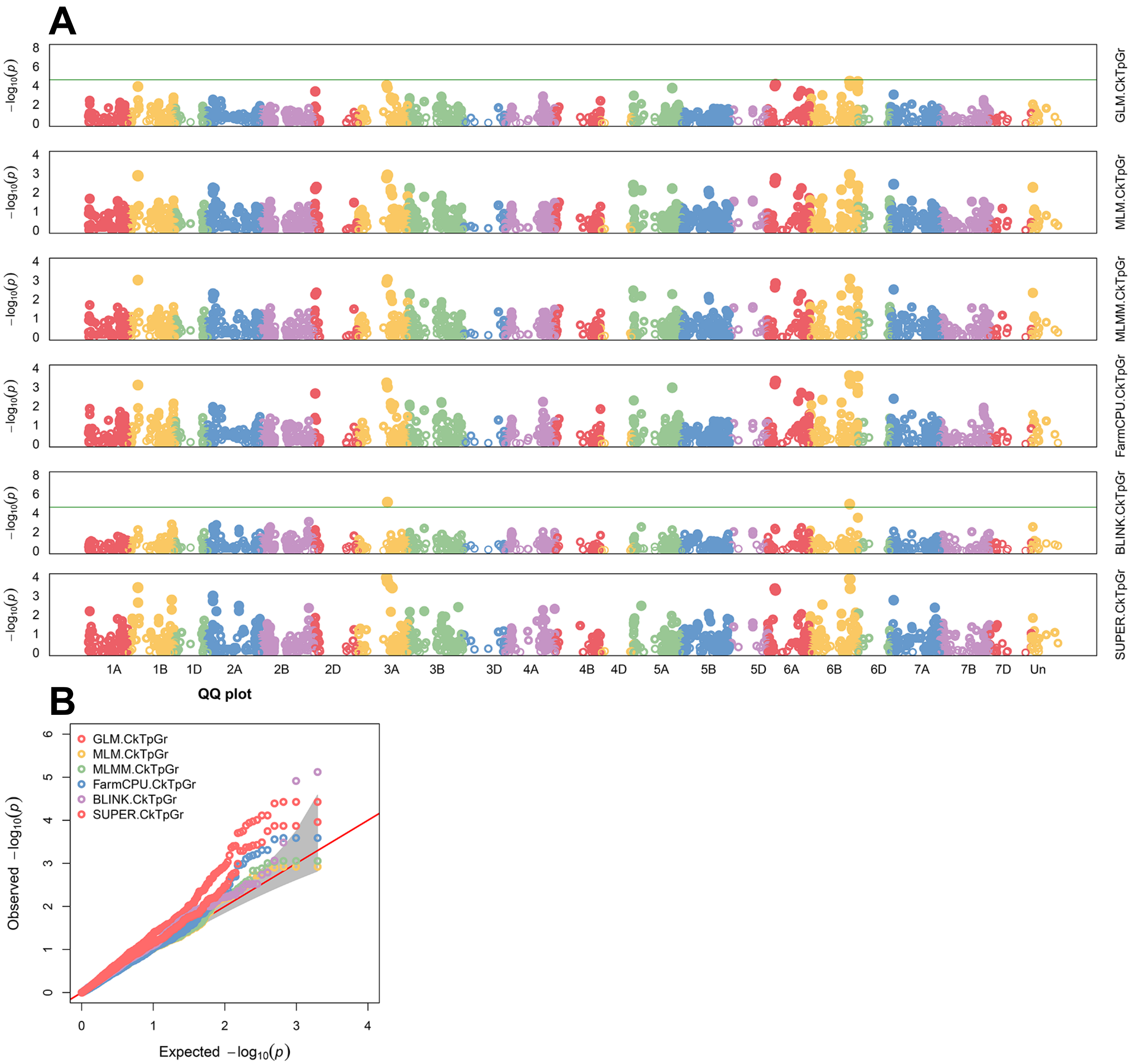

Fig. 16. Genome-wide association for Cookie Top Grade (CkTpGr). **A.** Manhattan plots for all six models, GLM, MLM, MLMM, FarmCPU, BLINK, and SUPER (Shown Right). Negative log 10 of p-value for each marker on a chromosome indicated by colored dots. Red horizontal line is the default, more stringent experiment-wise Bonferroni significance threshold in GAPIT3 of alpha = 0.01. Dashed grey vertical lines indicate two models significant for the same marker and solid grey vertical lines, three or more. **B.** QQ Plots for the genome-wide association. Colored circles represent the six different models tested for the trait. Red diagonal line indicates where observed and expected results would match. Grey shaded region is confidence interval and colored circles significantly above the line represent deviations that may be significantly associated with phenotype.

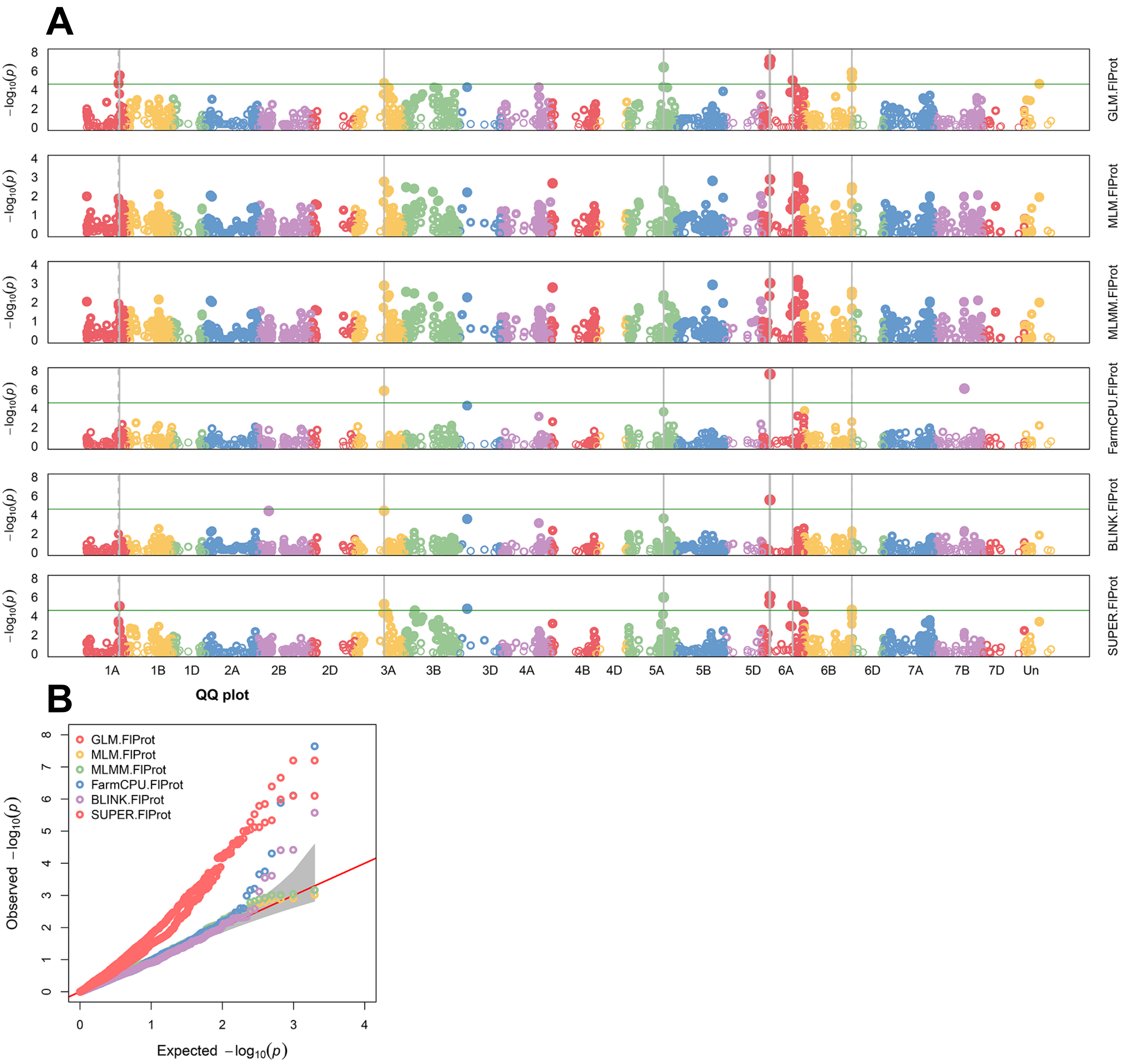

Fig. 17. Genome-wide association for Flour Protein (FlProt). **A.** Manhattan plots for all six models, GLM, MLM, MLMM, FarmCPU, BLINK, and SUPER (Shown Right). Negative log 10 of p-value for each marker on a chromosome indicated by colored dots. Red horizontal line is the default, more stringent experiment-wise Bonferroni significance threshold in GAPIT3 of alpha = 0.01. Dashed grey vertical lines indicate two models significant for the same marker and solid grey vertical lines, three or more. **B.** QQ Plots for the genome-wide association. Colored circles represent the six different models tested for the trait. Red diagonal line indicates where observed and expected results would match. Grey shaded region is confidence interval and colored circles significantly above the line represent deviations that may be significantly associated with phenotype.

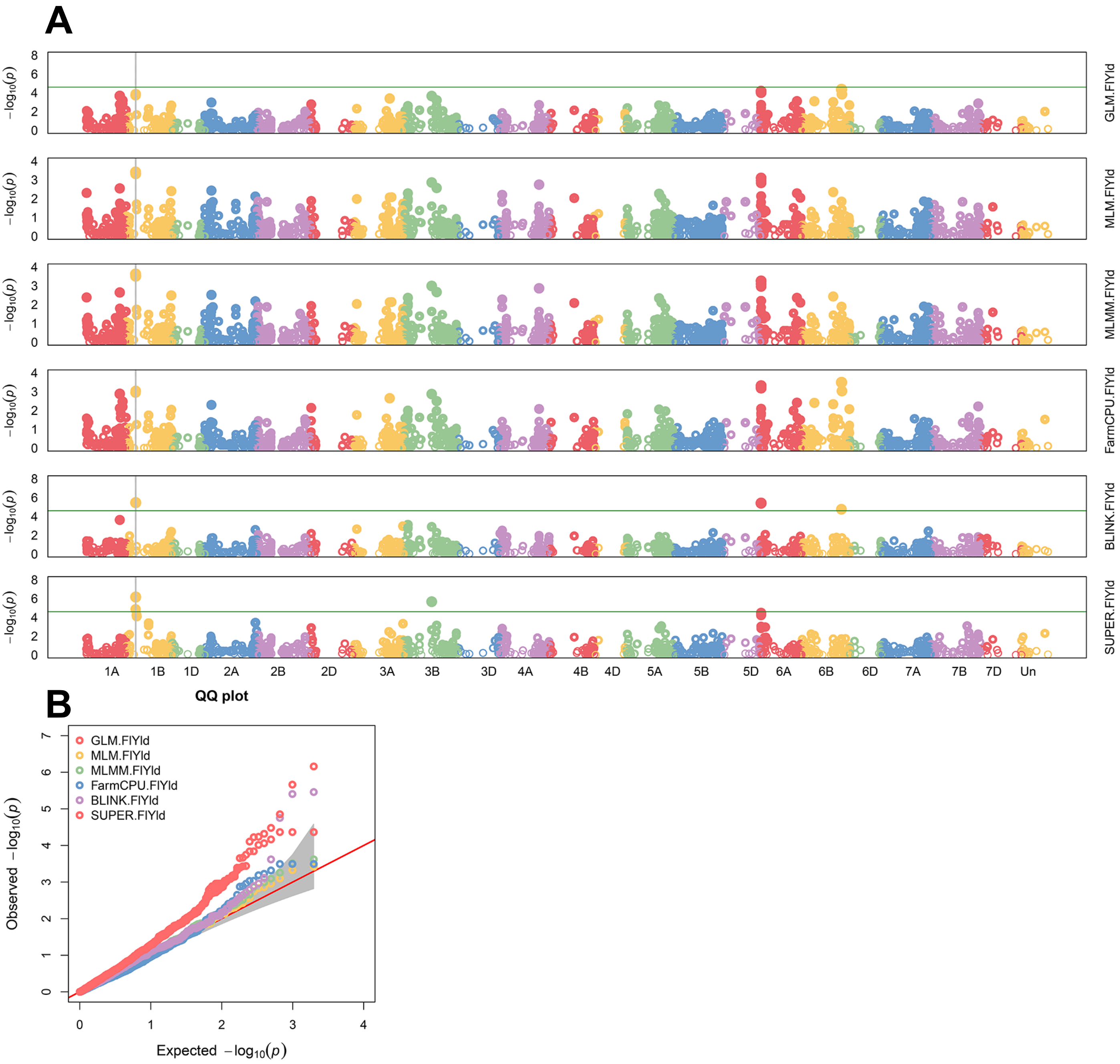

Fig. 18. Genome-wide association for Flour Yield (FlYld). **A.** Manhattan plots for all six models, GLM, MLM, MLMM, FarmCPU, BLINK, and SUPER (Shown Right). Negative log 10 of p-value for each marker on a chromosome indicated by colored dots. Red horizontal line is the default, more stringent experiment-wise Bonferroni significance threshold in GAPIT3 of alpha = 0.01. Dashed grey vertical lines indicate two models significant for the same marker and solid grey vertical lines, three or more. **B.** QQ Plots for the genome-wide association. Colored circles represent the six different models tested for the trait. Red diagonal line indicates where observed and expected results would match. Grey shaded region is confidence interval and colored circles significantly above the line represent deviations that may be significantly associated with phenotype.

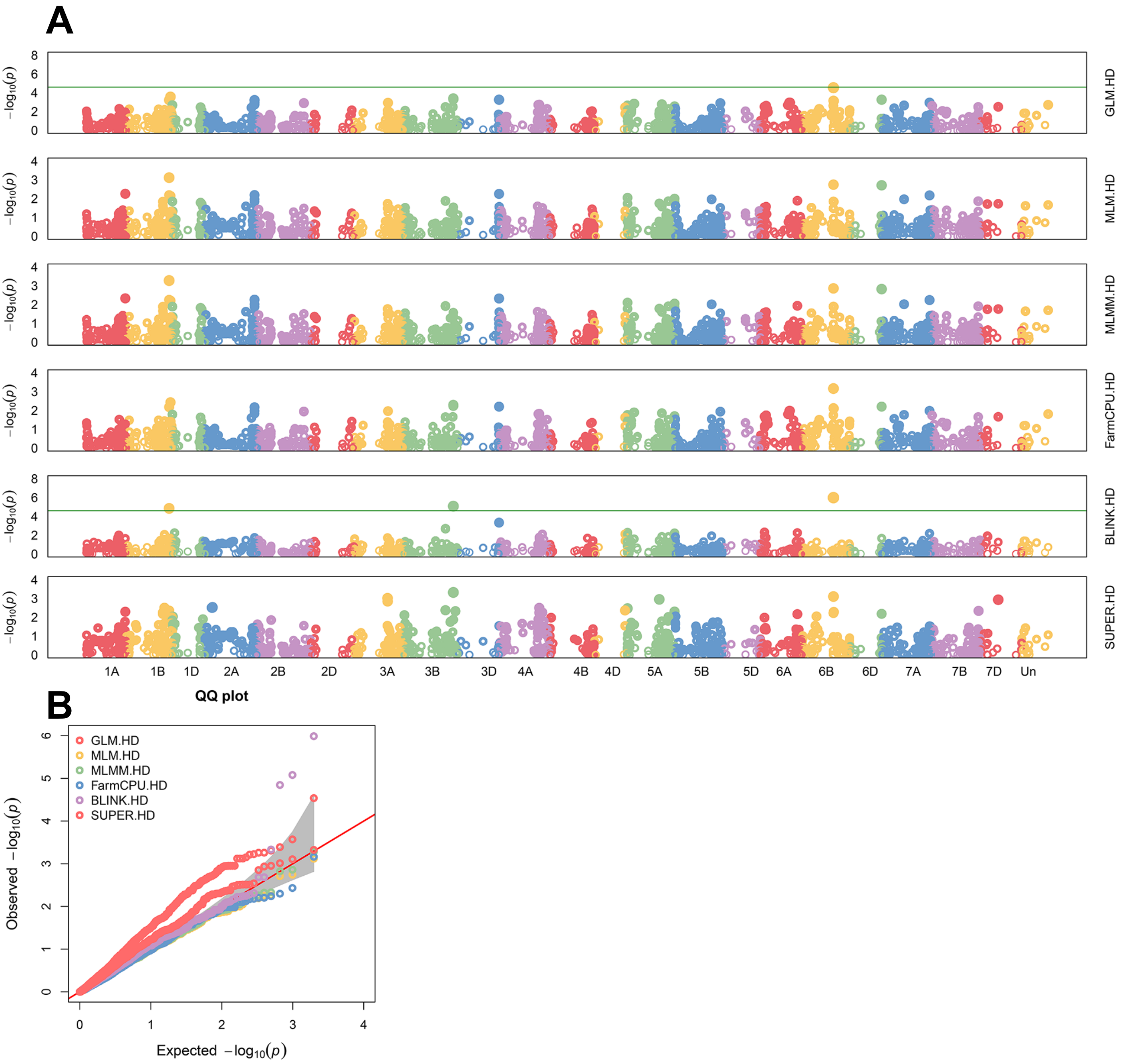

Fig. 19. Genome-wide association for Heading Days (HD). **A.** Manhattan plots for all six models, GLM, MLM, MLMM, FarmCPU, BLINK, and SUPER (Shown Right). Negative log 10 of p-value for each marker on a chromosome indicated by colored dots. Red horizontal line is the default, more stringent experiment-wise Bonferroni significance threshold in GAPIT3 of alpha = 0.01. Dashed grey vertical lines indicate two models significant for the same marker and solid grey vertical lines, three or more. **B.** QQ Plots for the genome-wide association. Colored circles represent the six different models tested for the trait. Red diagonal line indicates where observed and expected results would match. Grey shaded region is confidence interval and colored circles significantly above the line represent deviations that may be significantly associated with phenotype.

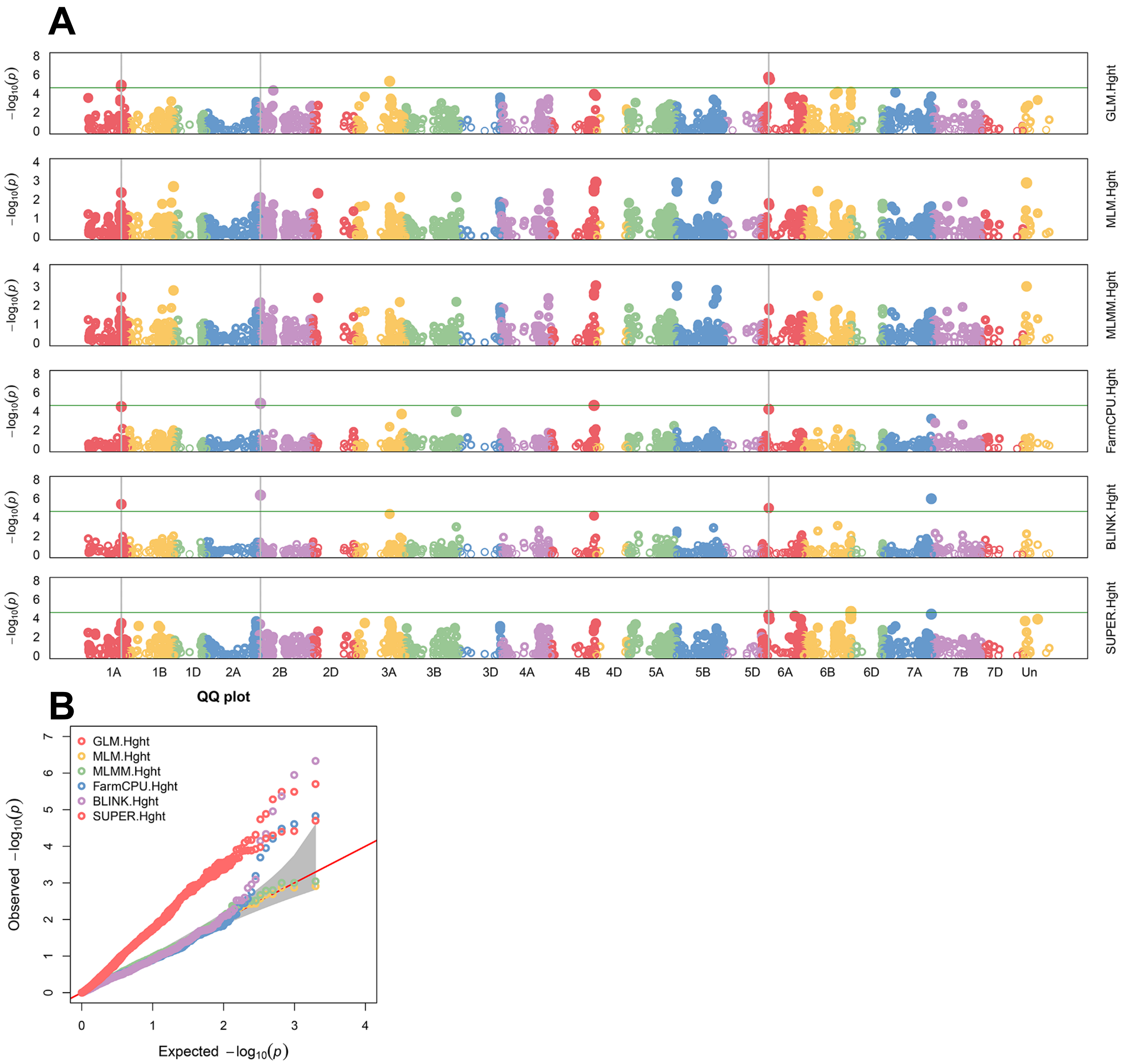

Fig. 20. Genome-wide association for Plant Height (Hght). **A.** Manhattan plots for all six models, GLM, MLM, MLMM, FarmCPU, BLINK, and SUPER (Shown Right). Negative log 10 of p-value for each marker on a chromosome indicated by colored dots. Red horizontal line is the default, more stringent experiment-wise Bonferroni significance threshold in GAPIT3 of alpha = 0.01. Dashed grey vertical lines indicate two models significant for the same marker and solid grey vertical lines, three or more. **B.** QQ Plots for the genome-wide association. Colored circles represent the six different models tested for the trait. Red diagonal line indicates where observed and expected results would match. Grey shaded region is confidence interval and colored circles significantly above the line represent deviations that may be significantly associated with phenotype.

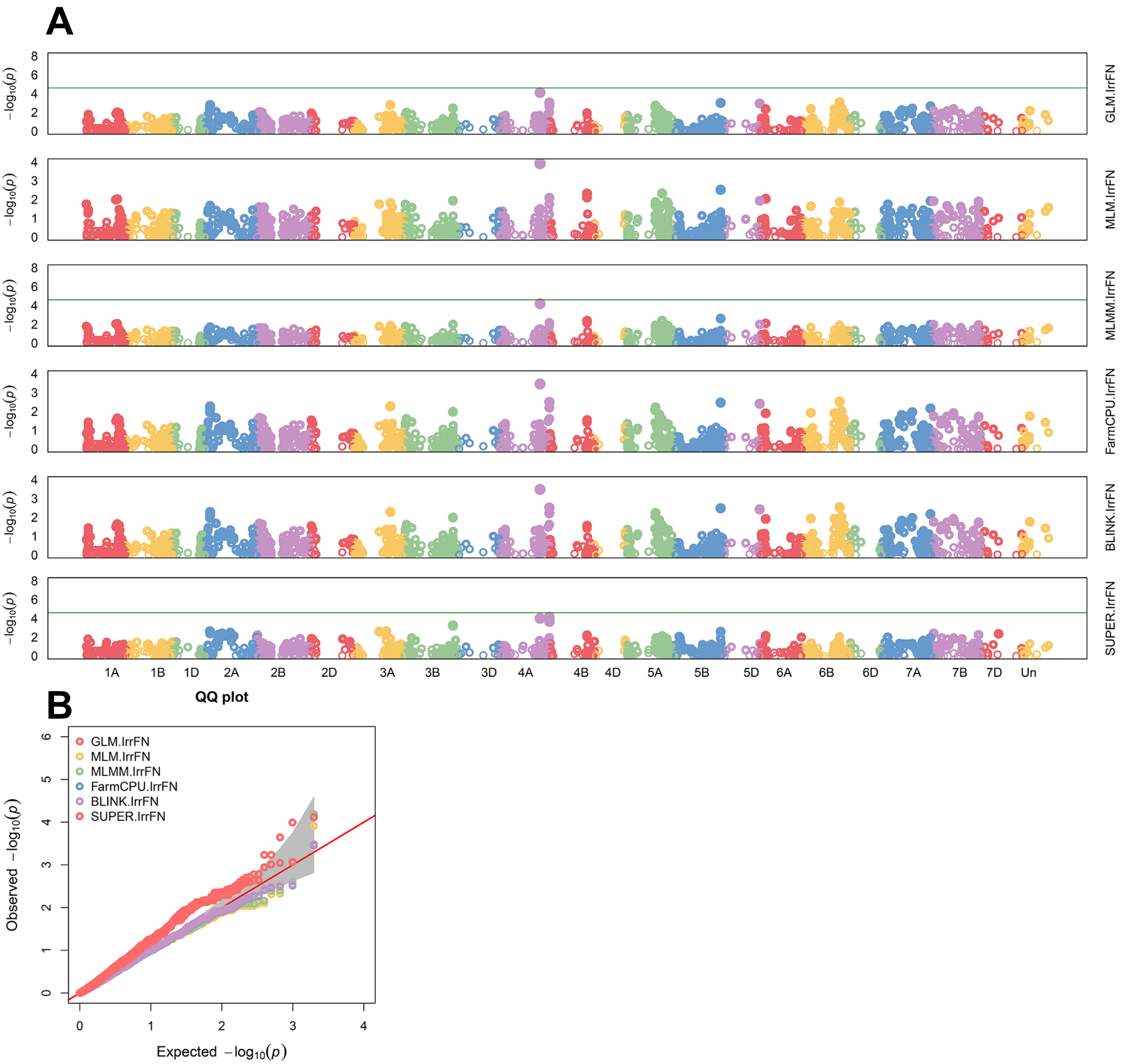

Fig. 21. Genome-wide association for Irrigated Falling Number (IrrFN). **A.** Manhattan plots for all six models, GLM, MLM, MLMM, FarmCPU, BLINK, and SUPER (Shown Right). Negative log 10 of p-value for each marker on a chromosome indicated by colored dots. Red horizontal line is the default, more stringent experiment-wise Bonferroni significance threshold in GAPIT3 of alpha = 0.01. Dashed grey vertical lines indicate two models significant for the same marker and solid grey vertical lines, three or more. **B.** QQ Plots for the genome-wide association. Colored circles represent the six different models tested for the trait. Red diagonal line indicates where observed and expected results would match. Grey shaded region is confidence interval and colored circles significantly above the line represent deviations that may be significantly associated with phenotype.

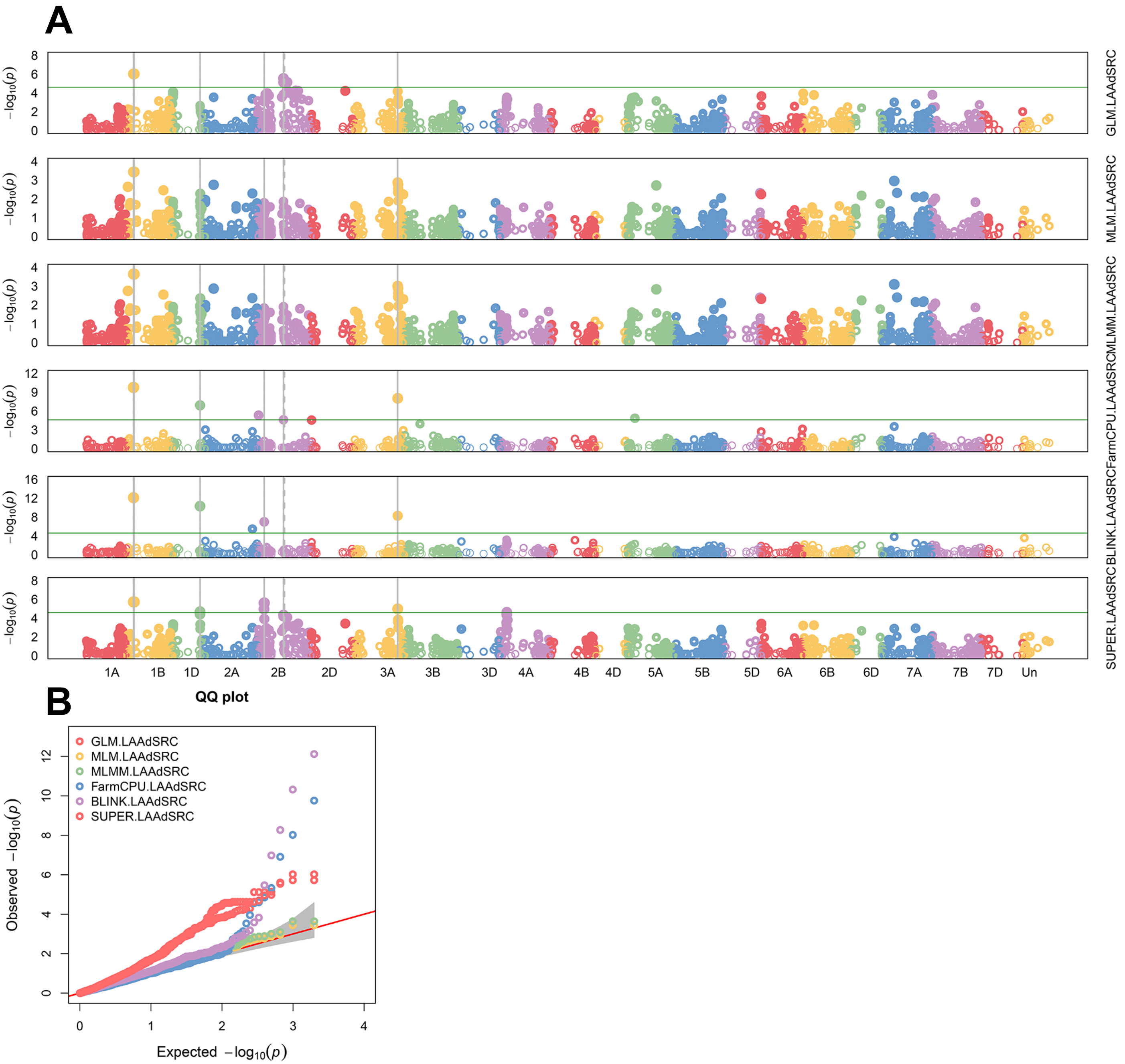

Fig. 22. Genome-wide association for Adjusted Lactic Acid Solvent Retention Capacity (LAAdSRC). **A.** Manhattan plots for all six models, GLM, MLM, MLMM, FarmCPU, BLINK, and SUPER (Shown Right). Negative log 10 of p-value for each marker on a chromosome indicated by colored dots. Red horizontal line is the default, more stringent experiment-wise Bonferroni significance threshold in GAPIT3 of alpha = 0.01. Dashed grey vertical lines indicate two models significant for the same marker and solid grey vertical lines, three or more. **B.** QQ Plots for the genome-wide association. Colored circles represent the six different models tested for the trait. Red diagonal line indicates where observed and expected results would match. Grey shaded region is confidence interval and colored circles significantly above the line represent deviations that may be significantly associated with phenotype.

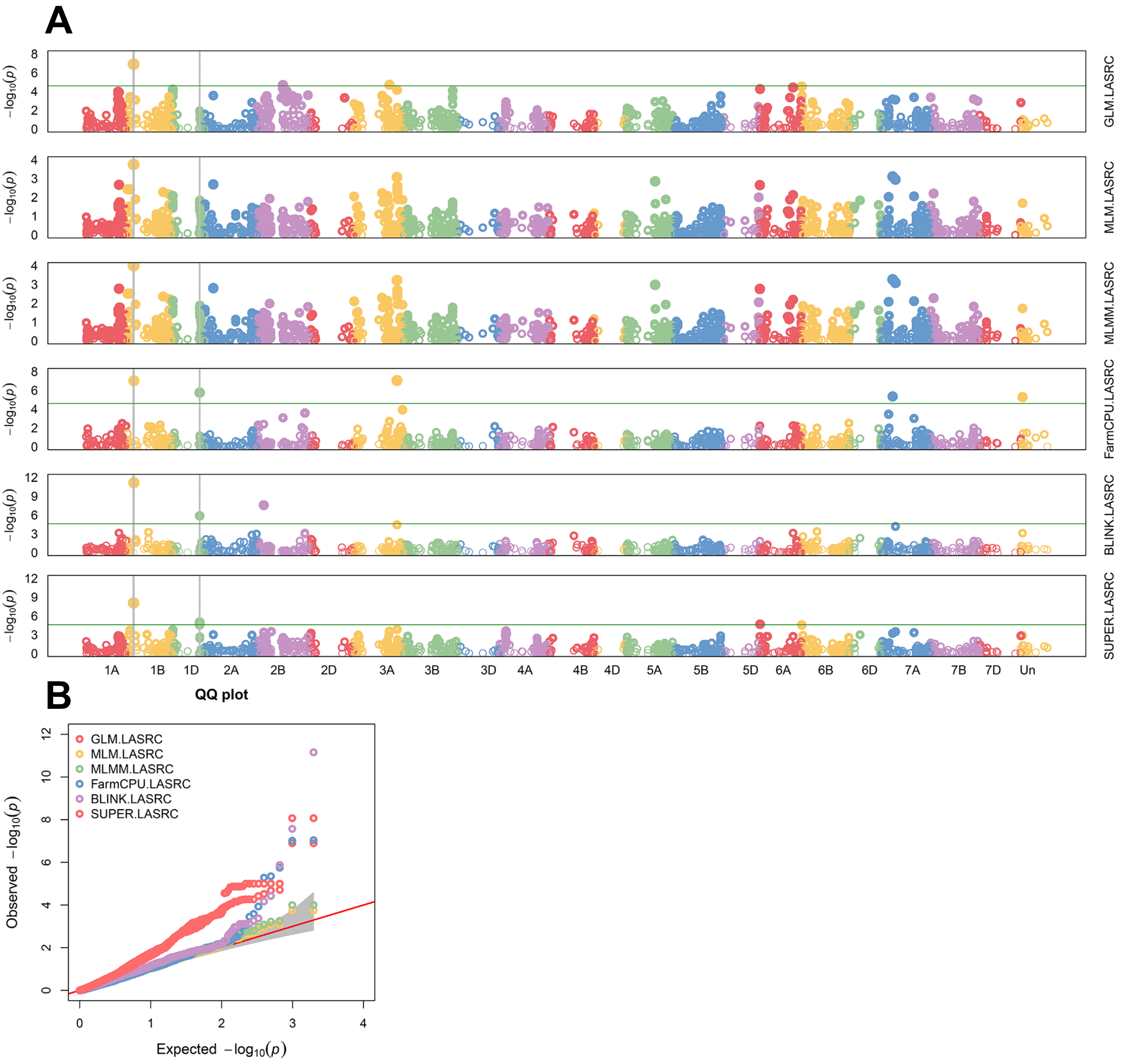

Fig. 23. Genome-wide association for Lactic Acid Solvent Retention Capacity (LASRC). **A.** Manhattan plots for all six models, GLM, MLM, MLMM, FarmCPU, BLINK, and SUPER (Shown Right). Negative log 10 of p-value for each marker on a chromosome indicated by colored dots. Red horizontal line is the default, more stringent experiment-wise Bonferroni significance threshold in GAPIT3 of alpha = 0.01. Dashed grey vertical lines indicate two models significant for the same marker and solid grey vertical lines, three or more. **B.** QQ Plots for the genome-wide association. Colored circles represent the six different models tested for the trait. Red diagonal line indicates where observed and expected results would match. Grey shaded region is confidence interval and colored circles significantly above the line represent deviations that may be significantly associated with phenotype.

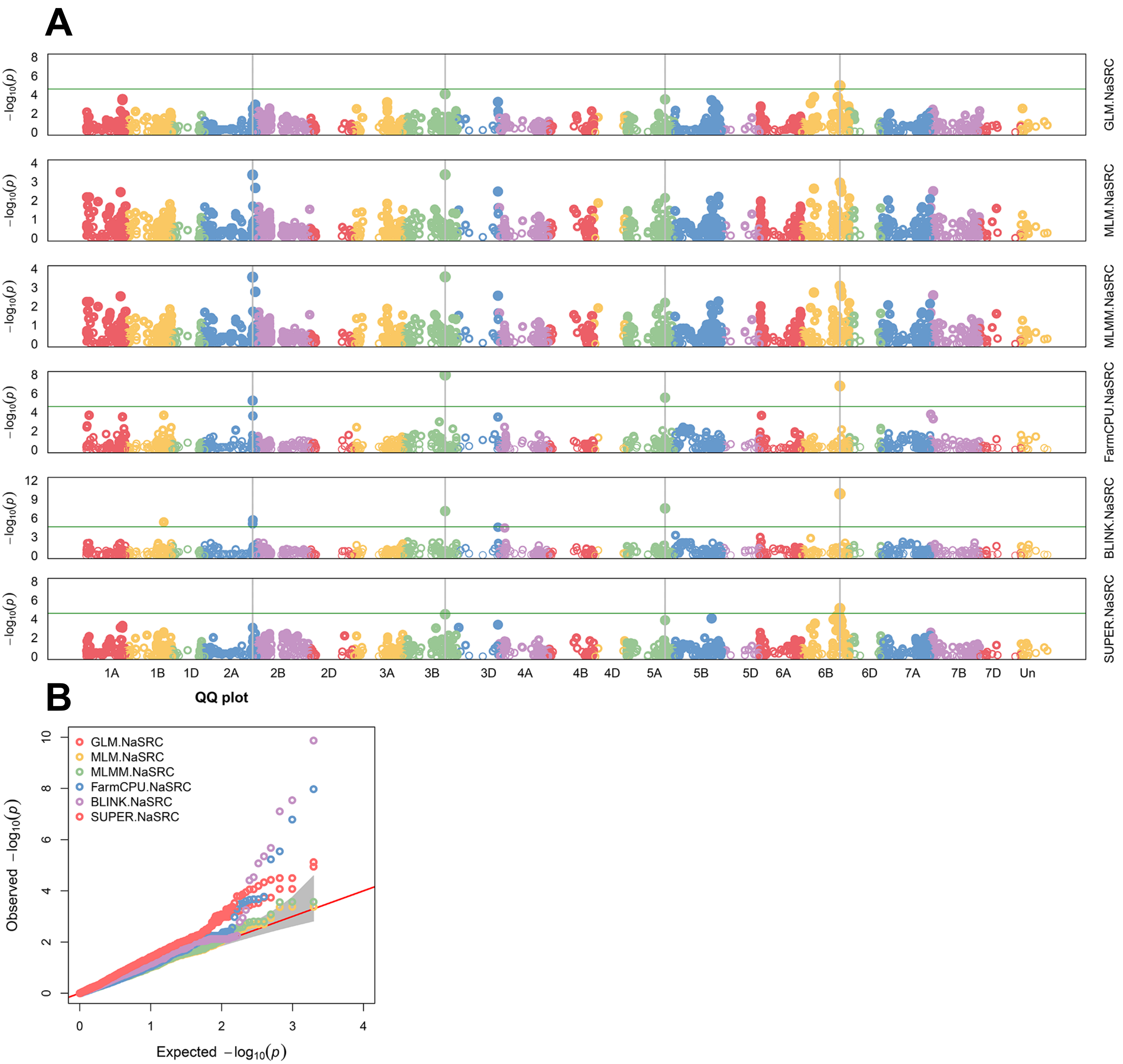

Fig. 24. Genome-wide association for Sodium Carbonate Solvent Retention Capacity (NaSRC). **A.** Manhattan plots for all six models, GLM, MLM, MLMM, FarmCPU, BLINK, and SUPER (Shown Right). Negative log 10 of p-value for each marker on a chromosome indicated by colored dots. Red horizontal line is the default, more stringent experiment-wise Bonferroni significance threshold in GAPIT3 of alpha = 0.01. Dashed grey vertical lines indicate two models significant for the same marker and solid grey vertical lines, three or more. **B.** QQ Plots for the genome-wide association. Colored circles represent the six different models tested for the trait. Red diagonal line indicates where observed and expected results would match. Grey shaded region is confidence interval and colored circles significantly above the line represent deviations that may be significantly associated with phenotype.

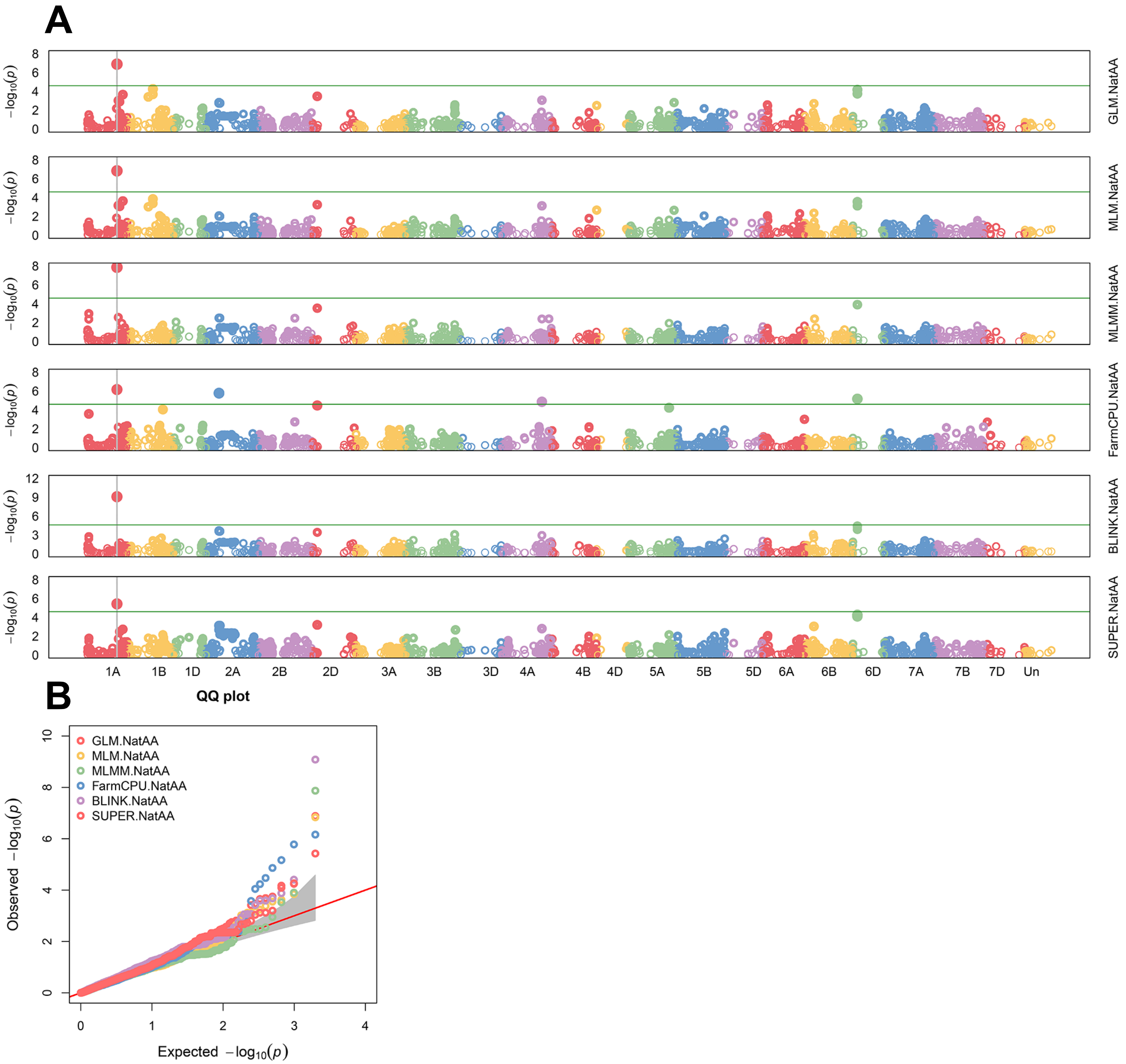

Fig. 25. Genome-wide association for Natural Weathering alpha amylase activity (NatAA). **A.** Manhattan plots for all six models, GLM, MLM, MLMM, FarmCPU, BLINK, and SUPER (Shown Right). Negative log 10 of p-value for each marker on a chromosome indicated by colored dots. Red horizontal line is the default, more stringent experiment-wise Bonferroni significance threshold in GAPIT3 of alpha = 0.01. Dashed grey vertical lines indicate two models significant for the same marker and solid grey vertical lines, three or more. **B.** QQ Plots for the genome-wide association. Colored circles represent the six different models tested for the trait. Red diagonal line indicates where observed and expected results would match. Grey shaded region is confidence interval and colored circles significantly above the line represent deviations that may be significantly associated with phenotype.

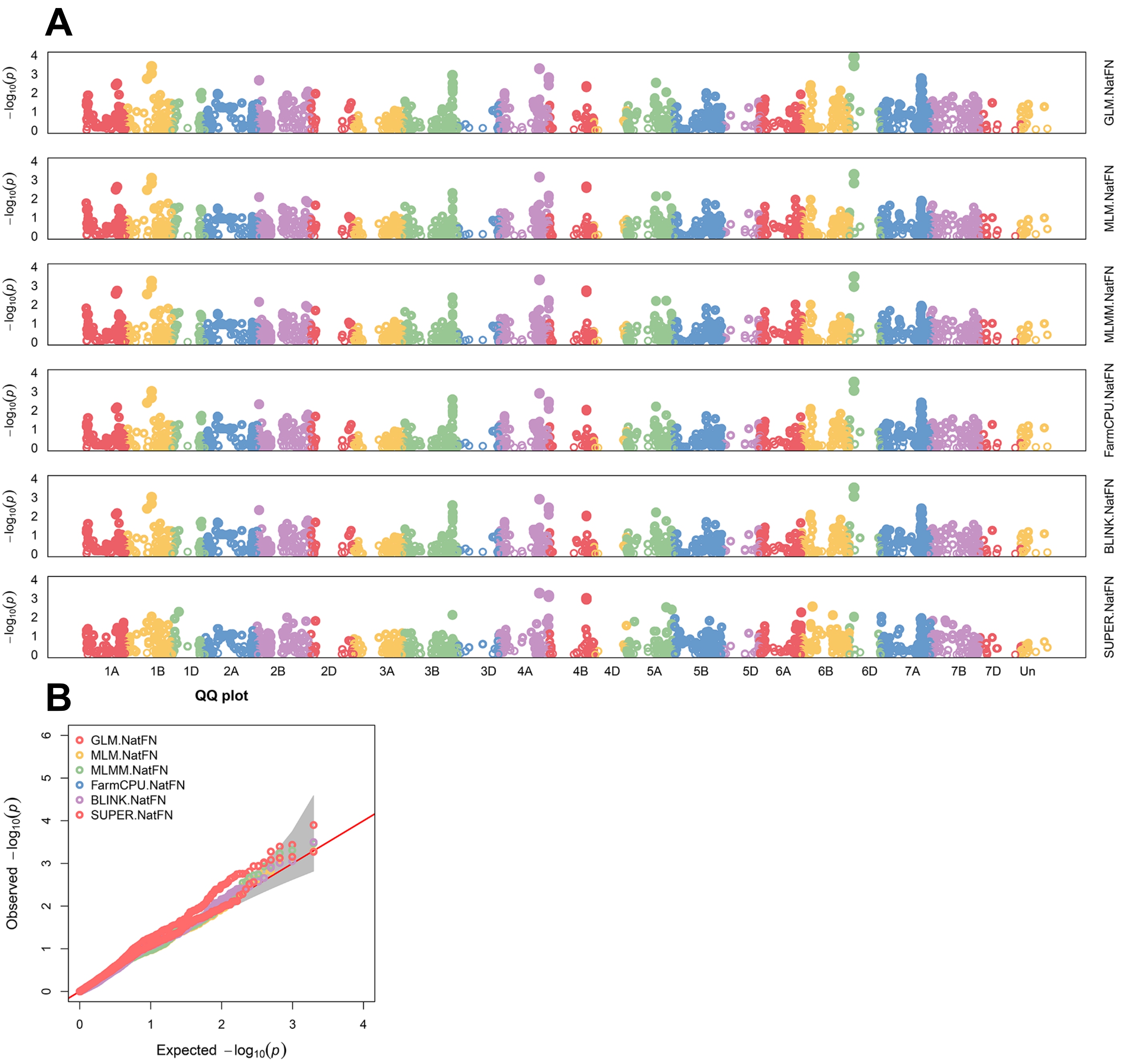

Fig. 26. Genome-wide association for Natural Weathering Falling Number (NatFN). **A.** Manhattan plots for all six models, GLM, MLM, MLMM, FarmCPU, BLINK, and SUPER (Shown Right). Negative log 10 of p-value for each marker on a chromosome indicated by colored dots. Red horizontal line is the default, more stringent experiment-wise Bonferroni significance threshold in GAPIT3 of alpha = 0.01. Dashed grey vertical lines indicate two models significant for the same marker and solid grey vertical lines, three or more. **B.** QQ Plots for the genome-wide association. Colored circles represent the six different models tested for the trait. Red diagonal line indicates where observed and expected results would match. Grey shaded region is confidence interval and colored circles significantly above the line represent deviations that may be significantly associated with phenotype.

A

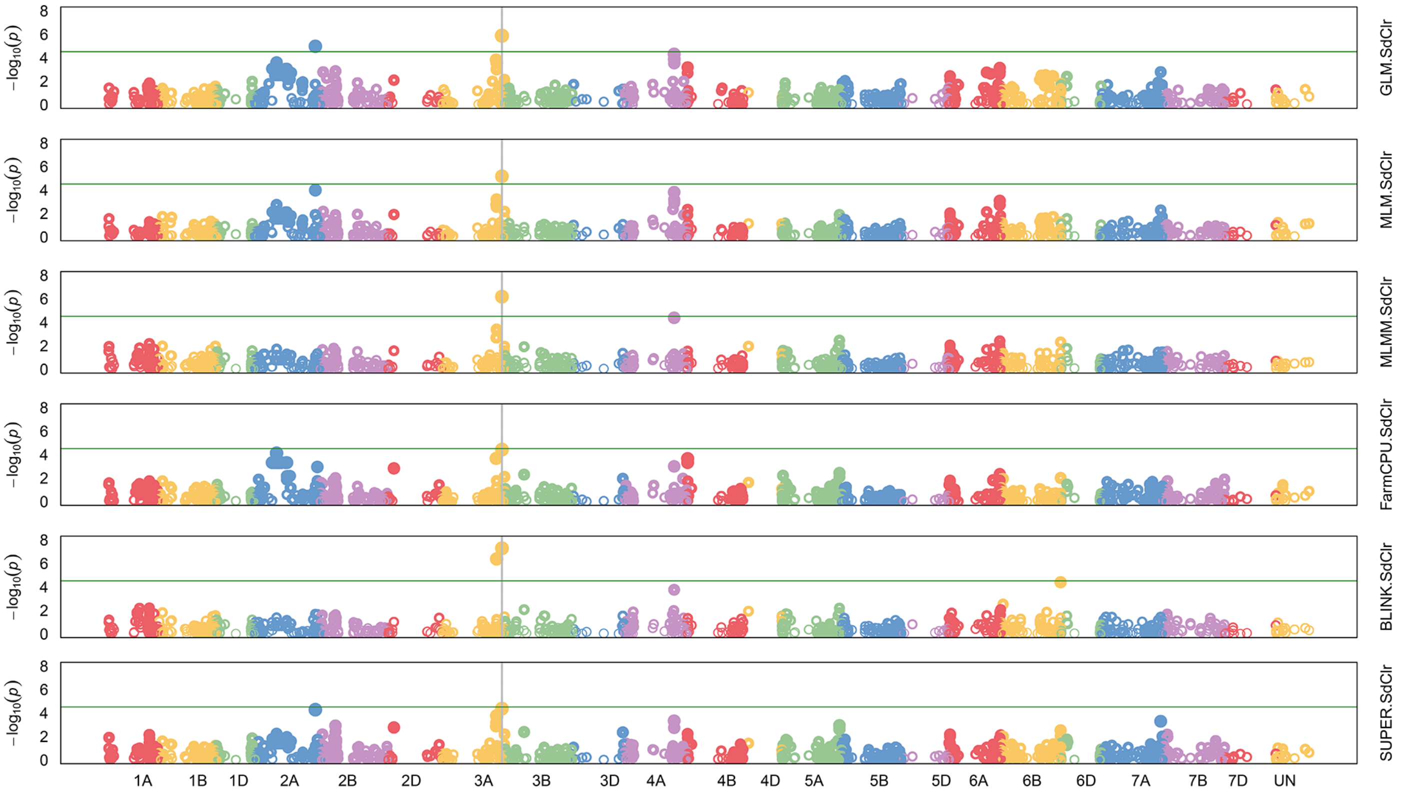

B

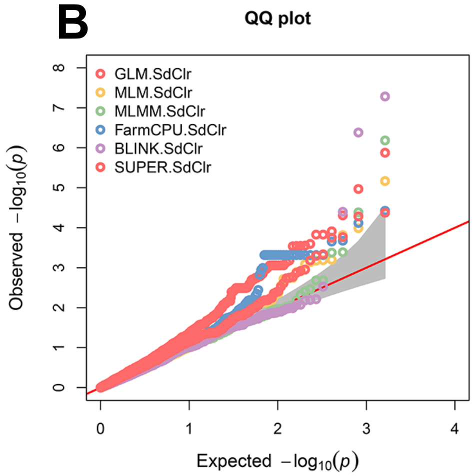

Fig. 27. Genome-wide association for Seed Color (SdClr). **A.** Manhattan plots for all six models, GLM, MLM, MLMM, FarmCPU, BLINK, and SUPER (Shown Right). Negative log 10 of p-value for each marker on a chromosome indicated by colored dots. Red horizontal line is the default, more stringent experiment-wise Bonferroni significance threshold in GAPIT3 of alpha = 0.01. Dashed grey vertical lines indicate two models significant for the same marker and solid grey vertical lines, three or more. **B.** QQ Plots for the genome-wide association. Colored circles represent the six different models tested for the trait. Red diagonal line indicates where observed and expected results would match. Grey shaded region is confidence interval and colored circles significantly above the line represent deviations that may be significantly associated with phenotype.

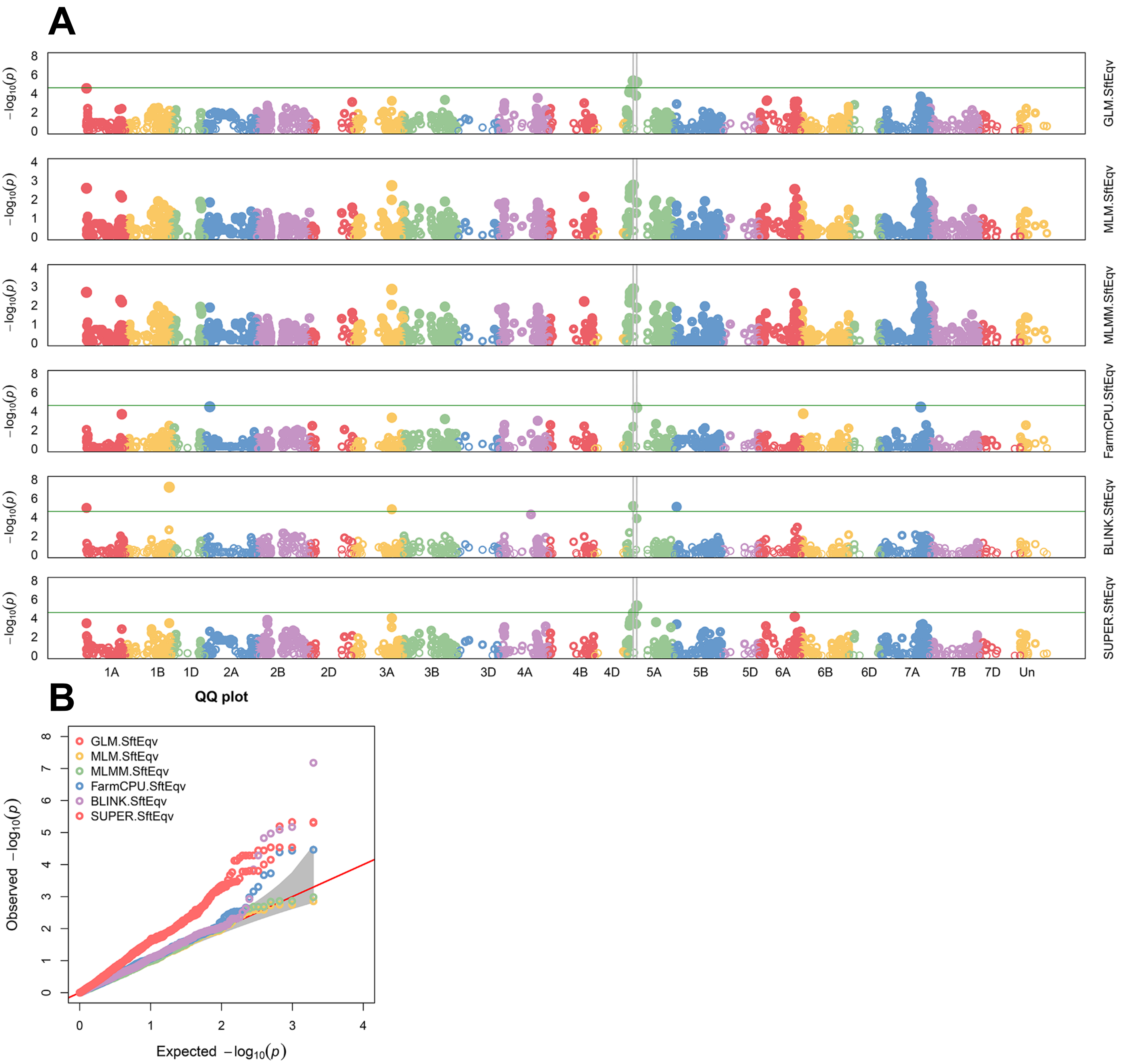

Fig. 28. Genome-wide association for Softness Equivalence (SftEqv). **A.** Manhattan plots for all six models, GLM, MLM, MLMM, FarmCPU, BLINK, and SUPER (Shown Right). Negative log 10 of p-value for each marker on a chromosome indicated by colored dots. Red horizontal line is the default, more stringent experiment-wise Bonferroni significance threshold in GAPIT3 of alpha = 0.01. Dashed grey vertical lines indicate two models significant for the same marker and solid grey vertical lines, three or more. **B.** QQ Plots for the genome-wide association. Colored circles represent the six different models tested for the trait. Red diagonal line indicates where observed and expected results would match. Grey shaded region is confidence interval and colored circles significantly above the line represent deviations that may be significantly associated with phenotype.

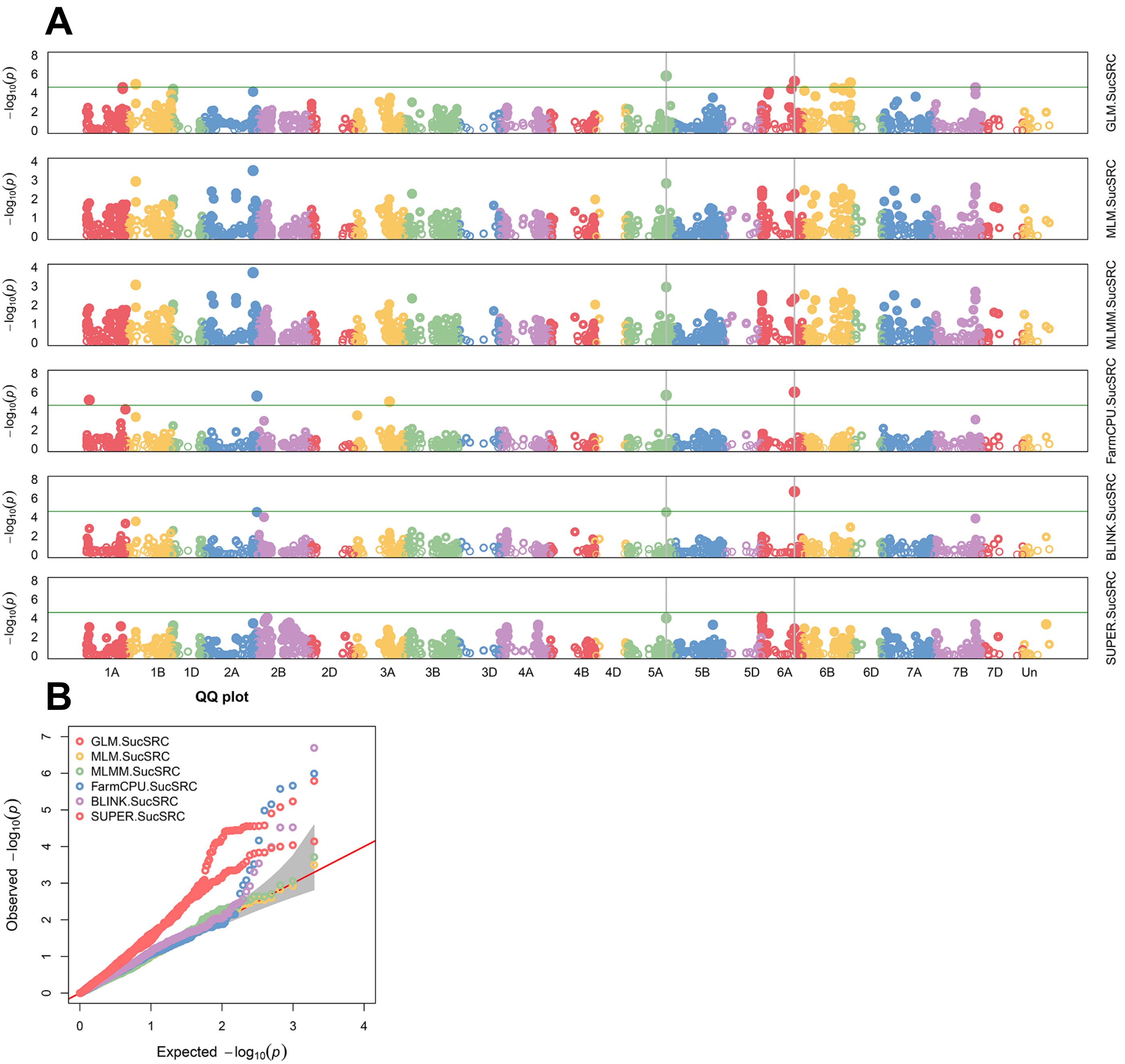

Fig. 29. Genome-wide association for Sucrose Solvent Retention Capacity (SucSRC). **A.** Manhattan plots for all six models, GLM, MLM, MLMM, FarmCPU, BLINK, and SUPER (Shown Right). Negative log 10 of p-value for each marker on a chromosome indicated by colored dots. Red horizontal line is the default, more stringent experiment-wise Bonferroni significance threshold in GAPIT3 of alpha = 0.01. Dashed grey vertical lines indicate two models significant for the same marker and solid grey vertical lines, three or more. **B.** QQ Plots for the genome-wide association. Colored circles represent the six different models tested for the trait. Red diagonal line indicates where observed and expected results would match. Grey shaded region is confidence interval and colored circles significantly above the line represent deviations that may be significantly associated with phenotype.

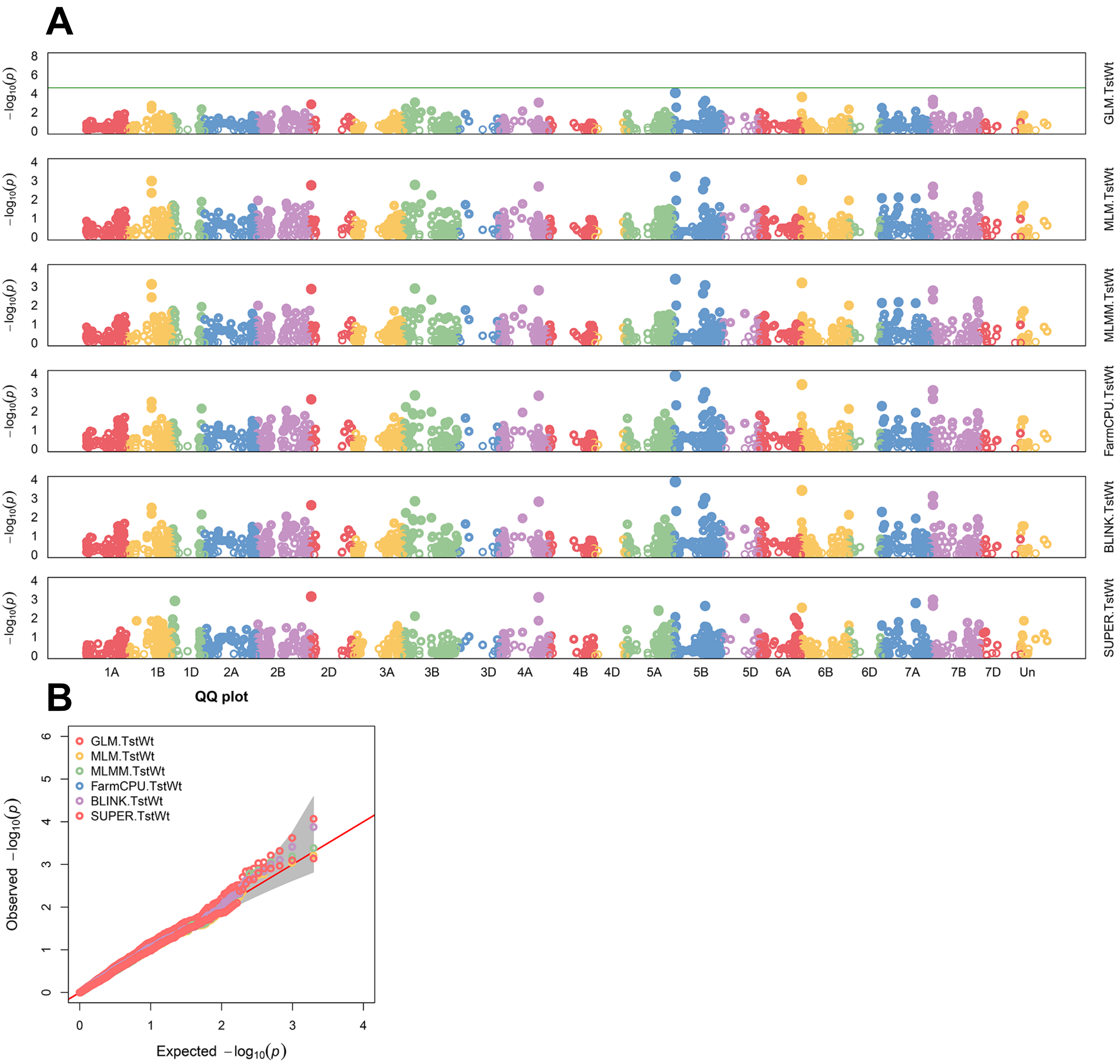

Fig. 30. Genome-wide association for Test Weight (TstWt). **A.** Manhattan plots for all six models, GLM, MLM, MLMM, FarmCPU, BLINK, and SUPER (Shown Right). Negative log 10 of p-value for each marker on a chromosome indicated by colored dots. Red horizontal line is the default, more stringent experiment-wise Bonferroni significance threshold in GAPIT3 of alpha = 0.01. Dashed grey vertical lines indicate two models significant for the same marker and solid grey vertical lines, three or more. **B.** QQ Plots for the genome-wide association. Colored circles represent the six different models tested for the trait. Red diagonal line indicates where observed and expected results would match. Grey shaded region is confidence interval and colored circles significantly above the line represent deviations that may be significantly associated with phenotype.

A

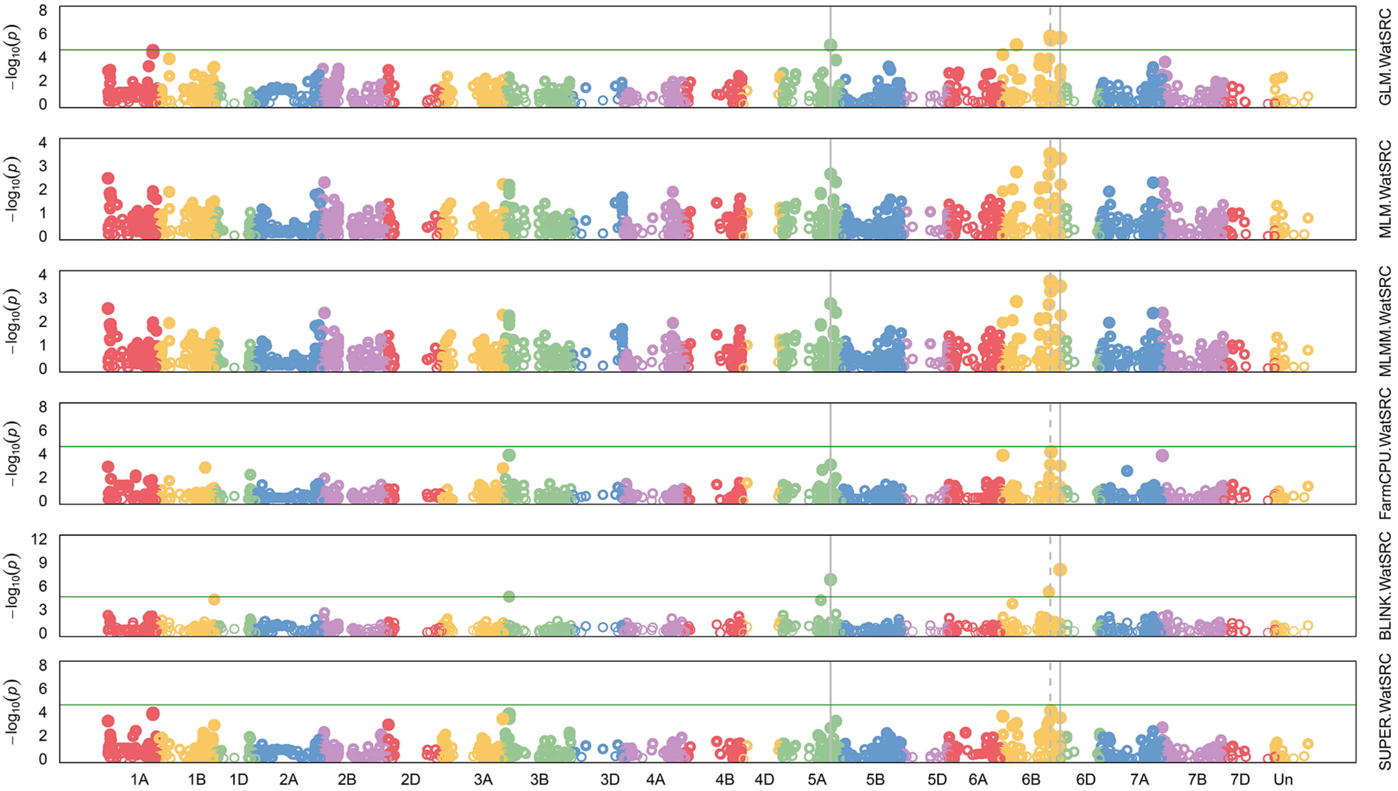

B

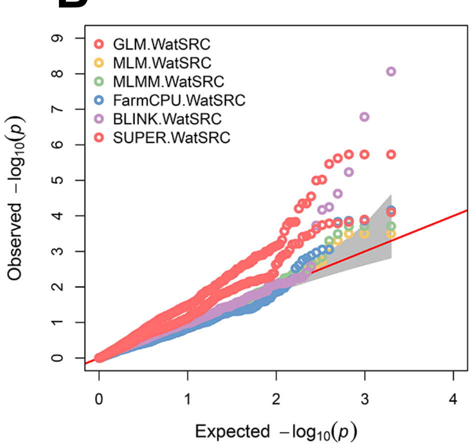

Fig. 31. Genome-wide association for Water Solvent Retention Capacity (WatSRC). **A.** Manhattan plots for all six models, GLM, MLM, MLMM, FarmCPU, BLINK, and SUPER (Shown Right). Negative log 10 of p-value for each marker on a chromosome indicated by colored dots. Red horizontal line is the default, more stringent experiment-wise Bonferroni significance threshold in GAPIT3 of alpha = 0.01. Dashed grey vertical lines indicate two models significant for the same marker and solid grey vertical lines, three or more. **B.** QQ Plots for the genome-wide association. Colored circles represent the six different models tested for the trait. Red diagonal line indicates where observed and expected results would match. Grey shaded region is confidence interval and colored circles significantly above the line represent deviations that may be significantly associated with phenotype.
