# Supplementary material for: Genetics of a diverse soft winter wheat population for pre-harvest sprouting, agronomic, and flour quality traits
Source: Front Plant Sci. 2023 Jun 6;14:1137808. doi: 10.3389/fpls.2023.1137808 (PMC10280069; doi:10.3389/fpls.2023.1137808)
Supplement: File S1 — Trait data set used to calculate BLUPs. Includes name, accession number, release year and all calculated values of traits including the means, standard deviations, and number of reps for each soft winter wheat variety used to calculate the BLUPs. [file DataSheet_1.zip › Supplementary Table S2.DOCX]

**Supplemental Table S2. Markers per chromosome, Bonferroni thresholds, number of significant QTN regions and trait-markers.**

| **Chrom** | **Number Markers** | **Significant 0.05^1^** | **QTN Regions** | **Significant Trait-Markers^2^** |
| --- | --- | --- | --- | --- |
| 1A | 137 | 3.65E-04 | 8 | 15 |
| 1B | 134 | 3.73E-04 | 6 | 13 |
| 1D | 55 | 9.09E-04 | 2 | 12 |
| 2A | 135 | 3.70E-04 | 5 | 8 |
| 2B | 165 | 3.03E-04 | 5 | 9 |
| 2D | 25 | 2.00E-03 | 6 | 8 |
| 3A | 112 | 4.46E-04 | 8 | 19 |
| 3B | 130 | 3.85E-04 | 5 | 6 |
| 3D | 17 | 2.94E-03 | 5 | 9 |
| 4A | 101 | 4.95E-04 | 4 | 11 |
| 4B | 55 | 9.09E-04 | 4 | 7 |
| 4D | 7 | 7.14E-03 | 2 | 3 |
| 5A | 142 | 3.52E-04 | 8 | 20 |
| 5B | 166 | 3.01E-04 | 1 | 1 |
| 5D | 22 | 2.27E-03 | 2 | 3 |
| 6A | 117 | 4.27E-04 | 5 | 21 |
| 6B | 118 | 4.24E-04 | 8 | 30 |
| 6D | 26 | 1.92E-03 | 3 | 7 |
| 7A | 168 | 2.98E-04 | 6 | 9 |
| 7B | 110 | 4.55E-04 | 4 | 9 |
| 7D | 16 | 3.13E-03 | 1 | 1 |
| Unlinked | 20 | 2.50E-03 | 4 | 5 |
| **Total** | **1978** | **2.53E-05** | **102** | **226** |

1: largest p-value where Bonferroni test is significant at alpha = 0.05.

2: Number of trait-markers, each trait added separately in case of multiple traits at same marker.
