# Supplementary figures and images for: Genetics of a diverse soft winter wheat population for pre-harvest sprouting, agronomic, and flour quality traits
Source: Front Plant Sci. 2023 Jun 6;14:1137808. doi: 10.3389/fpls.2023.1137808 (PMC10280069; doi:10.3389/fpls.2023.1137808)

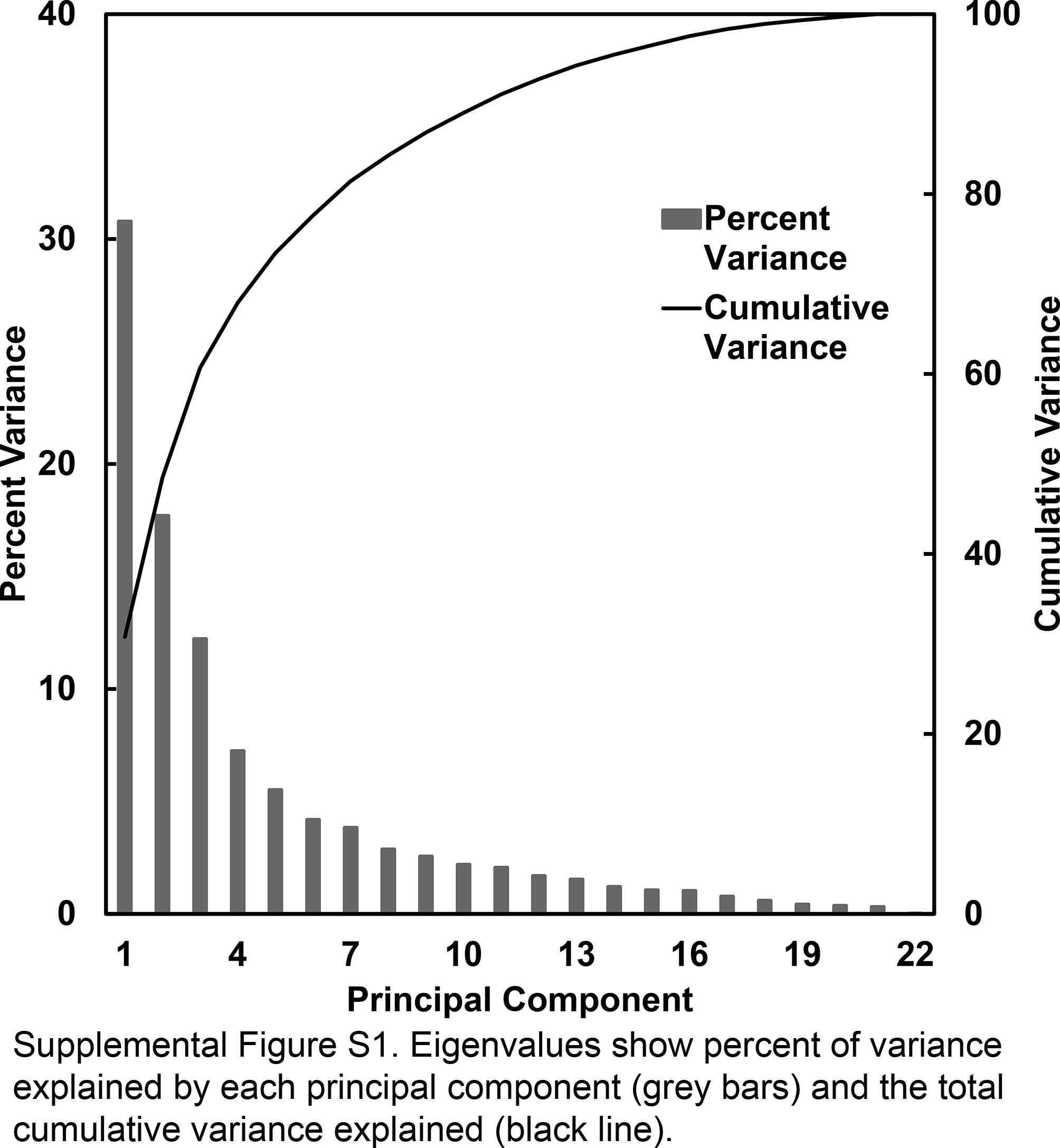

Supplement: File S1 — Trait data set used to calculate BLUPs. Includes name, accession number, release year and all calculated values of traits including the means, standard deviations, and number of reps for each soft winter wheat variety used to calculate the BLUPs. [file DataSheet_1.zip › Supplementary Figure S1.TIF]
